# Synthesis of functionalized spiro[indoline-3,4’-pyridines] and spiro[indoline-3,4’-pyridinones] via one-pot four-component reactions

**DOI:** 10.3762/bjoc.9.97

**Published:** 2013-05-02

**Authors:** Li-Juan Zhang, Qun Wu, Jing Sun, Chao-Guo Yan

**Affiliations:** 1 College of Chemistry & Chemical Engineering, Yangzhou University, Yangzhou 225002, China

**Keywords:** 1,4-dihydropyridine, electron-deficient alkyne, four-component reaction, isatin, one-pot reaction, spiro compound

## Abstract

In the presence of triethylamine as catalyst, the one-pot four-component reactions of arylamines, methyl propiolate, isatin and malononitrile afforded the functionalized spiro[indoline-3,4’-pyridine] derivatives in good yields. Similar reactions with ethyl cyanoacetate successfully afforded the functionalized spiro[indoline-3,4’-pyridines] and spiro[indoline-3,4’-pyridinones] as the main products according to the structures of the arylamines and other primary amines.

## Introduction

β-Enaminones and β-enamino esters represent important synthetic building blocks for the development of versatile carbon–carbon bond-formation reactions and heterocyclic constructions [1–5]. In recent years, the in situ generated β*-*enamino esters, which could be easily obtained from the addition of aliphatic or aromatic primary amines to the activated alkynes, have been widely recognized as practical synthons for the synthesis of a wide variety of heterocycles and pharmaceutical compounds [6–11]. Many domino reactions have been developed by trapping this kind of β*-*enamino ester with sequential adding of nucleophilic or electrophilic reagents to give versatile nitrogen-containing compounds and *N,O*-heterocycles [12–23]. Recently, Perumal and we have both developed an efficient synthetic procedure for functionalized spiro[indoline-3,4’-pyridines] by domino reactions of in situ generated β-enamino esters, isatin and malononitrile with triethylamine as the base catalyst [24–25]. A literature survey indicated that there has been an explosion of activity around the synthesis of spirooxindoles in the past years [26–31]. Encouraged by these results, and hunting for new synthetic methods for functionalized spirooxindoles, we investigated the domino reactions of arylamines, methyl propiolate, aromatic aldehydes and malononitrile (ethyl cyanoacetate) and successfully developed a facile synthetic procedure for functionalized spiro[indoline-3,4’-pyridines] and spiro[indoline-3,4’-pyridinones].

## Results and Discussion

**Figure 1:**
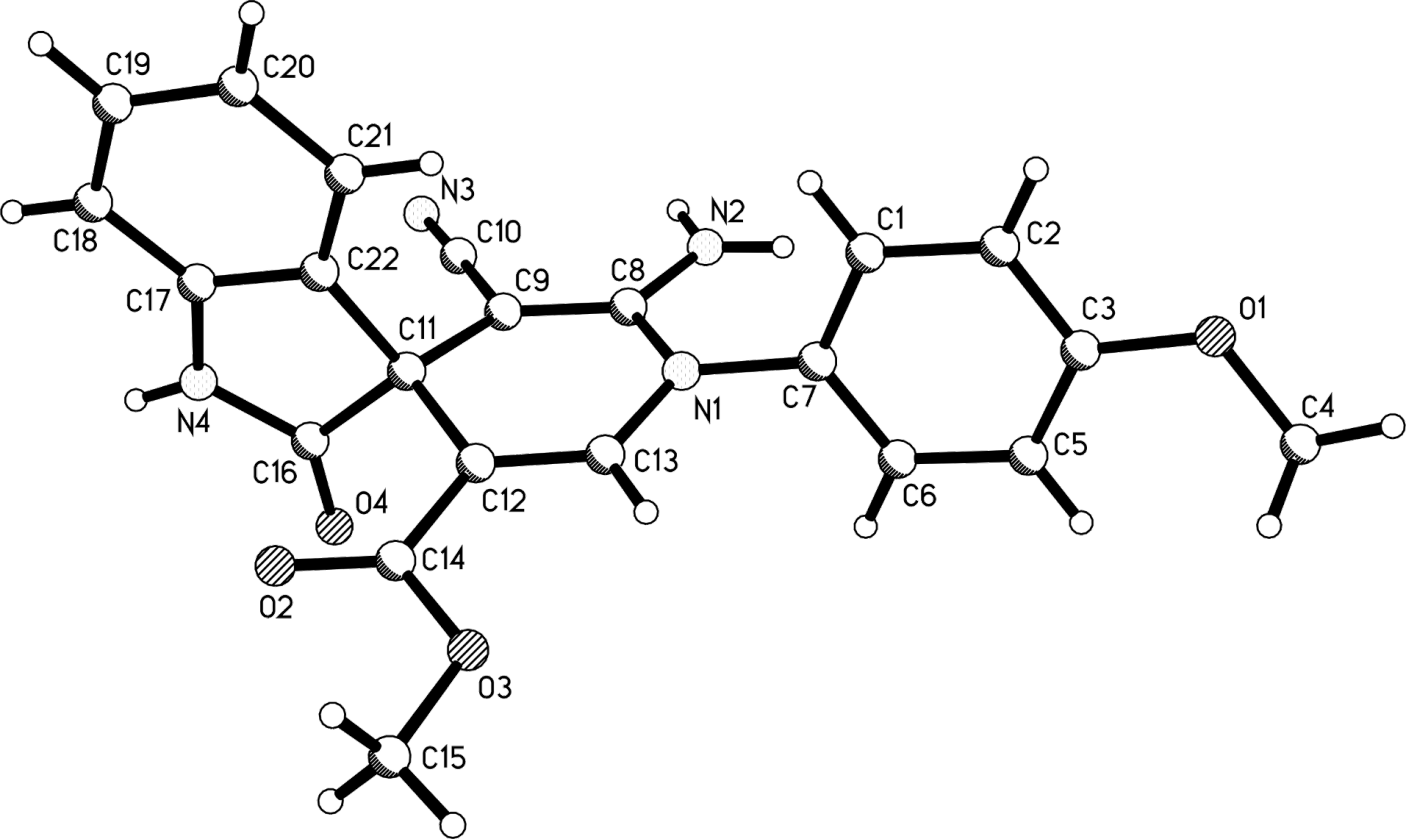
Molecular structure of the spiro compound **1c**.

**Figure 2:**
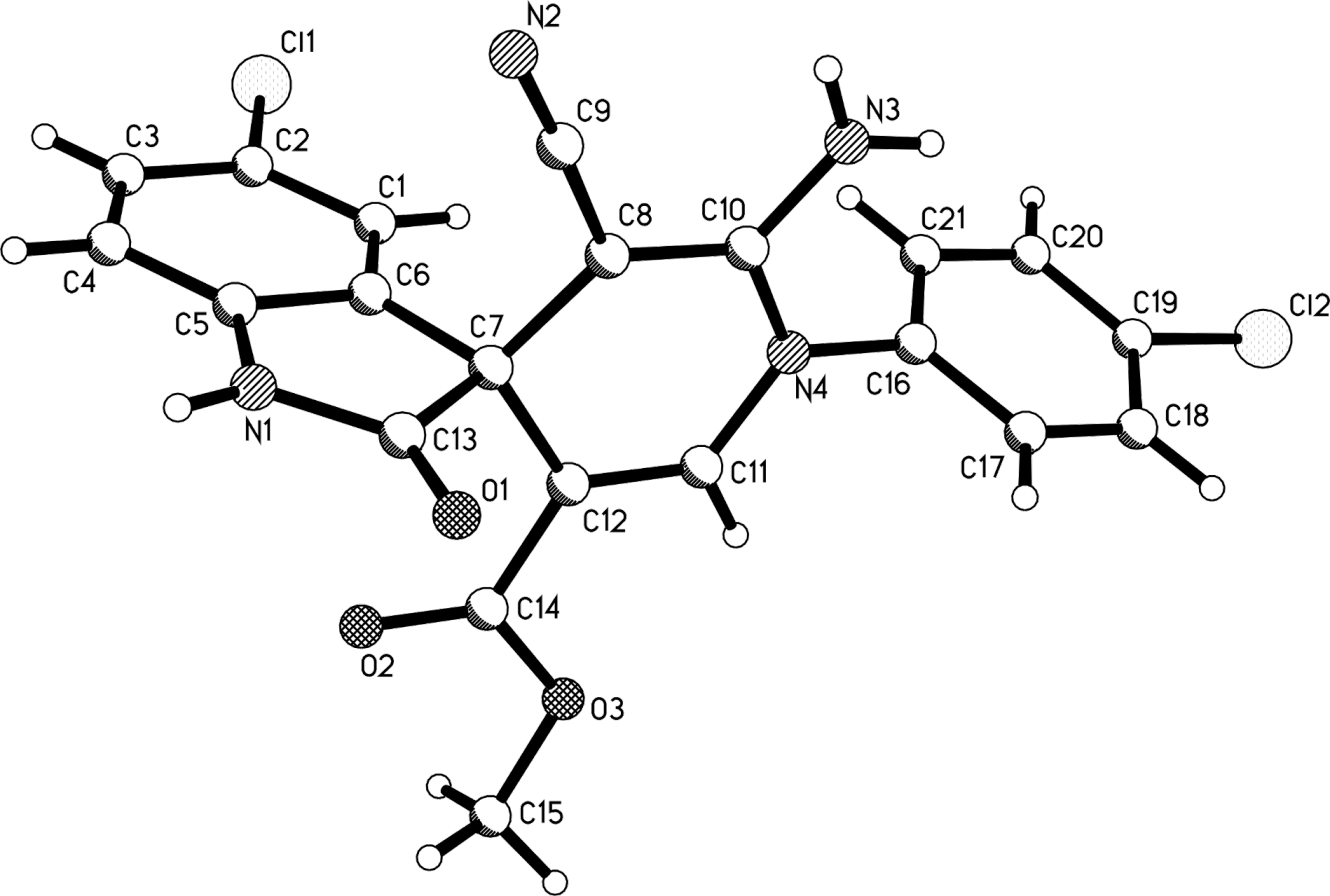
Molecular structure of the spiro compound **1h**.

The efficient formation of functionalized spiro[indole-3,4’-pyridines] via the four-component reaction prompted us to study the reaction scope further [25]. Another widely used electron-deficient alkyne reagent, methyl propiolate, was utilized to replace dimethyl acetylenedicarboxylate as one component. The addition reaction of aniline to methyl propiolate to give the active adducts, 3-arylaminoacrylates, usually needs more than twelve hours. Thus, we decided firstly to let arylamine and methyl propiolate react in ethanol at room temperature for 24 hours. TLC analysis indicated that the addition reaction had finished, and TLC analysis indicated that almost exclusively the desired β-arylaminoacrylates existed in the solution. Then isatin and malononitrile as well as triethylamine were added to the system and the mixture was heated under reflux for an additional 24 hours. The expected spiro[indoline-3,4’-pyridines] **1a**–**1p** were obtained in good yields by using this one-pot domino reaction procedure (Table 1). It can be seen that aniline bearing methoxy, methyl or chloro groups all reacted smoothly to give the expected spiro[indoline-3,4’-pyridines] with marginal effect. Benzylamine also afforded good yields of the spiro product. The structures of the prepared spiro[indoline-3,4’-pyridines] **1a**–**1p** were fully characterized by spectroscopic methods and were further confirmed by the determination of the single crystal structures of the spiro compounds **1c** (Figure 1) and **1h** (Figure 2).

**Table 1:** Synthesis of spiro[indoline-3,4’-pyridine] **1a**–**1p** by domino reaction.

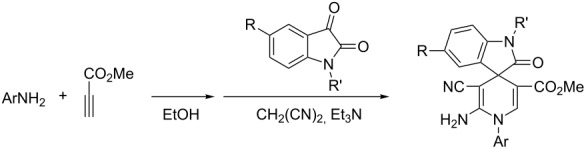					

Entry	Compd	Ar	R	R’	Yield (%)

1	**1a**	C_6_H_5_	H	H	79
2	**1b**	*p*-CH_3_C_6_H_4_	H	H	76
3	**1c**	*p*-CH_3_OC_6_H_4_	H	H	74
4	**1d**	*p-*ClC_6_H_4_	H	H	81
5	**1e**	*p*-BrC_6_H_4_	H	H	80
6	**1f**	*p-*ClC_6_H_4_	Me	H	75
7	**1g**	*p*-CH_3_C_6_H_4_	Me	H	73
8	**1h**	*p-*ClC_6_H_4_	Cl	H	84
9	**1i**	*p-*CH_3_C_6_H_4_	Cl	H	72
10	**1j**	*p-*ClC_6_H_4_	H	CH_2_C_6_H_5_	64
11	**1k**	*p-*CH_3_C_6_H_4_	H	CH_2_C_6_H_5_	75
12	**1l**	*p-*CH_3_OC_6_H_4_	H	CH_2_C_6_H_5_	72
13	**1m**	C_6_H_5_	H	CH_2_C_6_H_5_	66
14	**1n**	*m-*ClC_6_H_4_	H	CH_2_C_6_H_5_	61
15	**1o**	*m-*CH_3_C_6_H_4_	H	CH_2_C_6_H_5_	62
16	**1p**	C_6_H_5_CH_2_	H	CH_2_C_6_H_5_	50

**Scheme 1:**
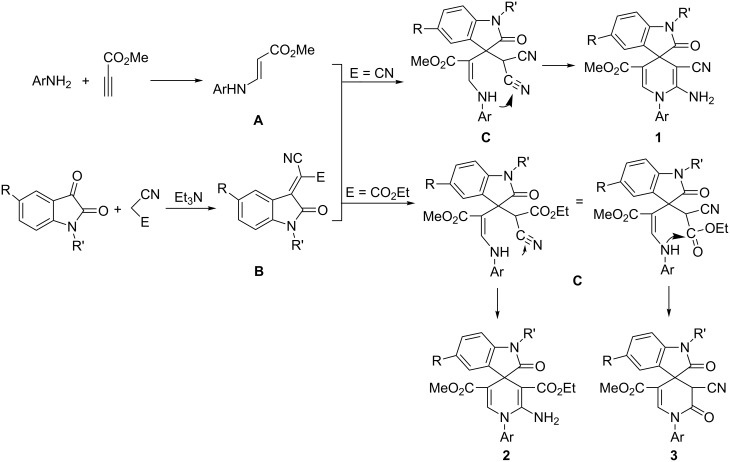
The proposed mechanism for the domino reaction.

When ethyl cyanoacetate was utilized in the domino reaction under similar conditions, the reaction usually resulted in a complicated mixture of spiro[indoline-3,4’-pyridines] **2** and spiro[indoline-3,4’-pyridinones] **3** depending on whether the cyano group or the ester group was taking part in the cyclization process (Scheme 1). In order to simplify the separation process, only the main product was separated from the reaction mixtures by column chromatography. The other product was not separated or structurally characterized. The results are listed in Table 2. It is interesting to find that anilines bearing *p*-chloro, *m*-chloro or *p*-bromo groups afforded spiro[indoline-3,4’-pyridines] **2a**–**2h** as the main products (Table 2, entries 1–8). Due to fact that *m*-nitroaniline and *p*-nitroaniline could not react with methyl propiolate to give the desired intermediate β-enamino ester, they were not utilized in this reaction. On the other hand *p*-methoxyaniline, *p*-methylaniline and aniline itself gave spiro[indoline-3,4’-pyridinones] **3a**–**3j** as the main products (Table 2, entries 9–18). Thus, it seems that anilines with electron-withdrawing groups preferably give spiro[indoline-3,4’-pyridines] **2**, while anilines with electron-donating groups preferably produce spiro[indoline-3,4’-pyridinones] **3**.

**Table 2:** Synthesis of spiro[indoline-3,4’-pyridines] **2a**–**2h** and spiro[indoline-3,4’-pyridinone] **3a**–**3n** via domino reaction.

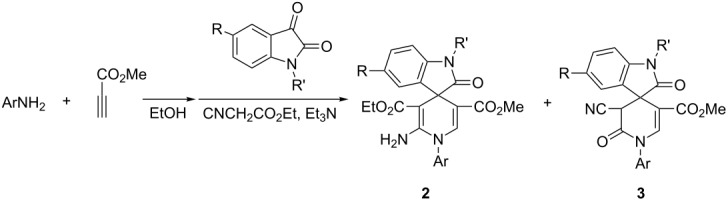					

Entry	Compd	Ar	R	R^’^	Yield (%)

1	**2a**	*p*-ClC_6_H_4_	H	H	49
2	**2b**	*p-*ClC_6_H_4_	H	CH_2_C_6_H_5_	52
3	**2c**	*p*-ClC_6_H_4_	Me	C_4_H_9_	47
4	**2d**	*p*-ClC_6_H_4_	Me	CH_2_C_6_H_5_	35
5	**2e**	*m*-ClC_6_H_4_	Me	CH_2_C_6_H_5_	36
6	**2f**	*p*-BrC_6_H_4_	H	H	52
7	**2g**	*p*-BrC_6_H_4_	H	C_4_H_9_	36
8	**2h**	*p*-BrC_6_H_4_	Me	CH_2_C_6_H_5_	56
9	**3a**	*p*-CH_3_OC_6_H_4_	H	H	39
10	**3b**	*p*-CH_3_OC_6_H_4_	H	CH_2_C_6_H_5_	53
11	**3c**	*p*-CH_3_OC_6_H_4_	H	C_4_H_9_	54
12	**3d**	*p*-CH_3_OC_6_H_4_	Me	CH_2_C_6_H_5_	51
13	**3e**	*p*-CH_3_C_6_H_4_	H	CH_2_C_6_H_5_	56
14	**3f**	*p*-CH_3_C_6_H_4_	H	C_4_H_9_	47
15	**3g**	*p*-CH_3_C_6_H_4_	Me	CH_2_C_6_H_5_	49
16	**3h**	C_6_H_5_	Me	C_4_H_9_	56
17	**3i**	C_6_H_5_	H	CH_2_C_6_H_5_	47
18	**3j**	C_6_H_5_	Me	CH_2_C_6_H_5_	52
19	**3k**	C_6_H_5_CH_2_	H	H	68
20	**3l**	C_6_H_5_CH_2_	Me	CH_2_C_6_H_5_	45
21	**3m**	C_6_H_5_CH_2_	H	C_4_H_9_	63
22	**3n**	C_6_H_5_CH_2_CH_2_	H	CH_2_C_6_H_5_	48

**Figure 3:**
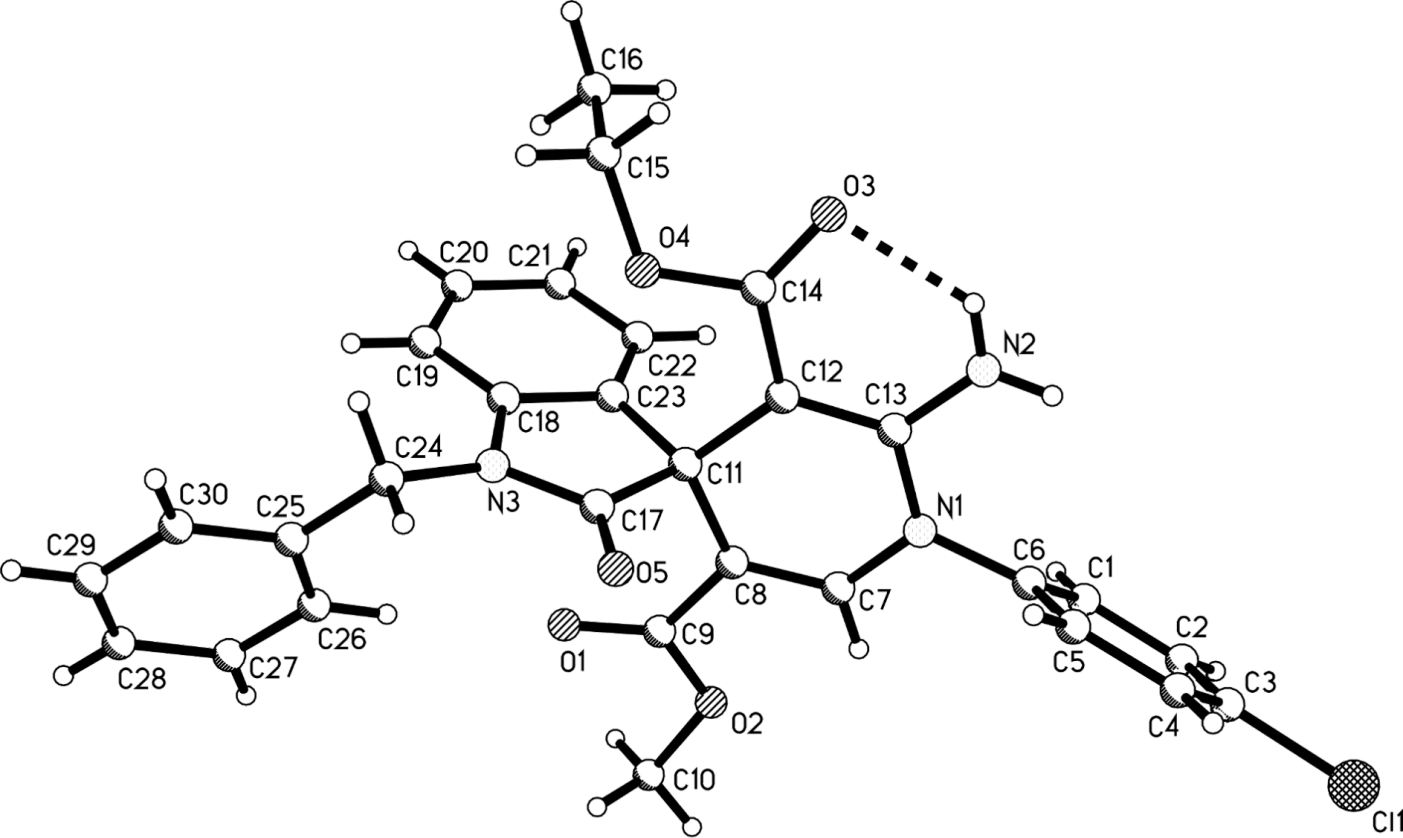
Molecular structure of spiro compound **2b**.

**Figure 4:**
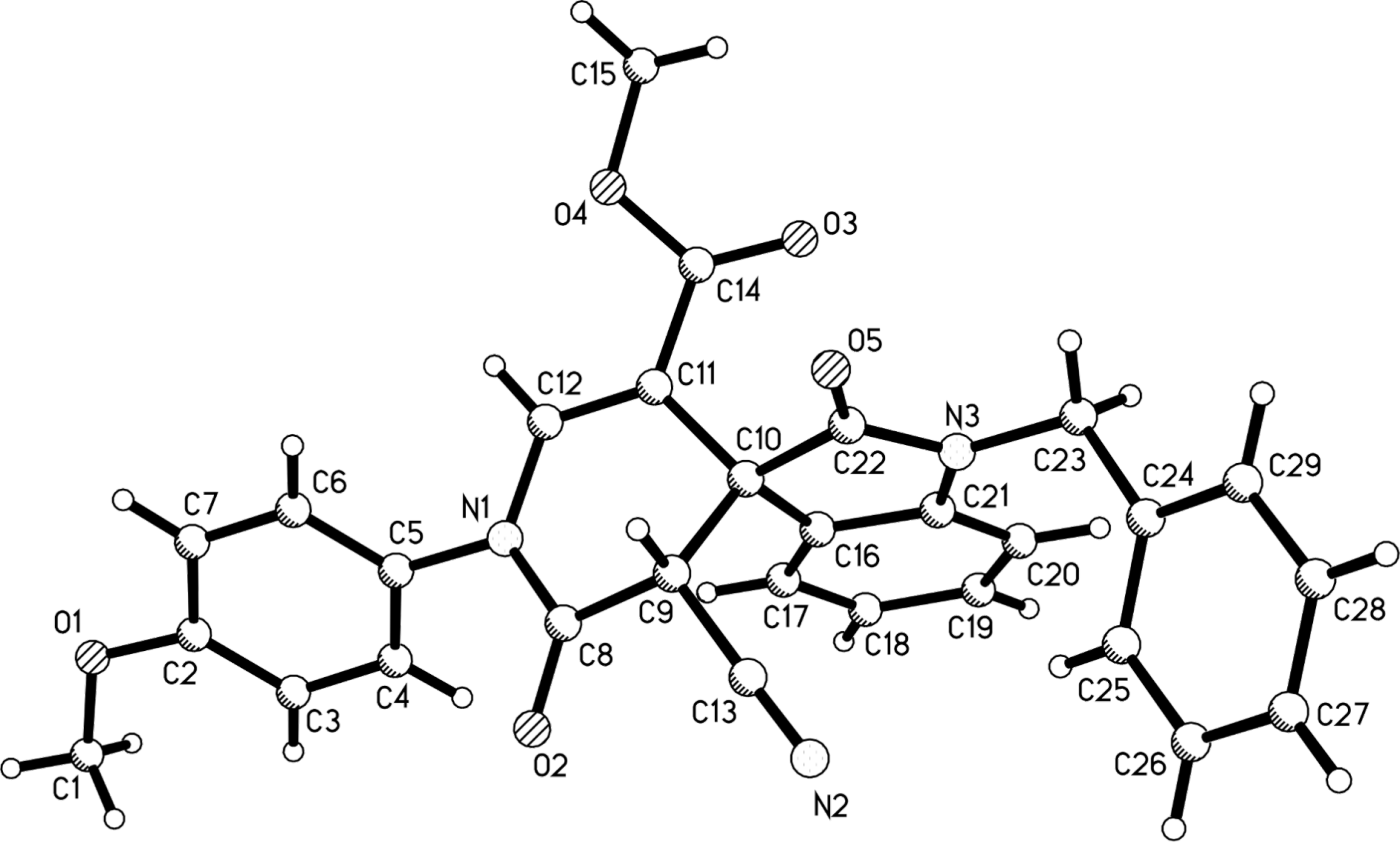
Molecular structure of spiro compound **3b**.

Benzylamine and 2-phenylethylamine could also be used in the domino reactions to give the spiro[indoline-3,4’-pyridinones] **3k**–**3n** as the main products (Table 2, entries 19–22). The structures of the prepared spiro compounds **2a**–**2h** and **3a**–**3n** were fully established by spectroscopic methods. The single-crystal structures of spiro compounds **2b** (Figure 3) and **3b** (Figure 4) were successfully determined by X-ray diffraction methods. It should be pointed out that the ^1^H NMR spectra of compounds **2a**–**2h** showed some distinguishing features. The characteristic resonance of the NH_2_ group usually displays one broad peak at about 7.40 ppm, which is overlapped with the peaks of aromatic protons. Comparing with the one singlet at 5.80 ppm of the NH_2_ group in compounds **1a**–**1p**, the peak of the NH_2_ group in compounds **2a**–**2h** is shifted to a much lower field. Secondly, the characteristic peaks of the CH_2_ unit of the ethoxy group in most spiro[indoline-3,4’-pyridines] **2a**–**2h** split into two mixed peaks at about 3.75–3.72 (m, 1H), 3.37–3.34 (m, 1H), which indicated that these two protons existed in different circumstance. The CH_3_ unit of the ethoxy goup showed a normal peak.

From the molecular structure of the spiro compound **3b** shown in Figure 4, we could clearly see that the cyano group and the phenyl group of oxindole moiety in the newly formed dihydropyridinone ring exist in *cis*-configuration. ^1^H NMR spectra of **3a**–**3n** all display one singlet at about 5.40 ppm for the one proton in the dihydropyridinone ring, which indicated that only one isomer exists in each product. Based on the single crystal structure and ^1^H NMR data we could tentatively conclude that the spiro[indoline-3,4’-pyridinones] **3a**–**3n** have *cis*-configuration of the cyano group and the phenyl group of the oxindole moiety.

To explain the mechanism of this domino reaction, a plausible reaction course was proposed to account for the different products based on the published reactions containing methyl propiolate [20–22], which are illustrated in Scheme 1. The first reaction is the formation of the key intermediate β-enamino ester **A** from the addition of arylamine to methyl propiolate. The second reaction is a Knoevenagel condensation of isatin with malononitrile or ethyl cyanoacetate under the catalysis of triethylamine to give the isatylidene deriatives **B**. The third reaction is a Michael addition of β-enamino ester intermediate **A** with isatinylidene derivative **B** to yield intermediate **C**. In the case of the reaction containing malononitrile, the nucleophilic addition of the amino group to the C–N triple bond in intermediate **C** resulted in spiro compound **1** with the tautomerization of the imino group to an amino group. In the case of the reaction containing ethyl cyanoacetate**,** the amino group could react with both the cyano group and the ester group in the adduct **C**. The nucleophilic addition of the amino group to the C–N triple bond finally afforded spiro[indoline-3,4’-pyridines] **2**. On the other hand the amino group attacked the ester group to give spiro[indoline-3,4’-pyridinones] **3** with the elimination of ethanol. In this reaction process, the reasons that anilines with electron-withdrawing groups preferably attack the cyano group and anilines with electron-donating groups preferably attack the ester group are not very clear.

## Conclusion

In summary, we have successfully developed a one-pot four-component reaction of arylamines, methyl propiolate, isatin and malononitrile or ethyl cyanoacetate with triethylamine as base catalyst. This reaction can proceed smoothly under mild conditions to afford the functionalized spiro[indoline-3,4’-pyridines] and spiro[indoline-3,4’-pyridinones] in moderate to good yields. The advantages of this reaction included readily available starting materials, mild reaction conditions, operational simplicity, a widely variety of substrates, and molecular diversity of the products. The potential uses of this reaction in synthetic and medicinal chemistry may be quite significant.

## Experimental

**Reagents and apparatus**: Aromatic aldehydes, arylamines, methyl propiolate and other reagents are commercial reagents and were used as received. Solvents were purified by standard techniques. All reactions were monitored by TLC. Melting points were taken on a hot-plate microscope apparatus. IR spectra were obtained on a Bruker Tensor 27 spectrometer (KBr disc). NMR spectra were recorded with a Bruker AV-600 spectrometer. HPLC–MS were measured with a Fennigan LCQ Deca XP MAX instrument. High-resolution mass spectra (ESI) were obtained with a Bruker UHR–TOF maXis spectrometer. X-ray data were collected on a Bruker Smart APEX-2 CCD diffractometer. Single-crystal data for compounds **1c** (CCDC 843674), **1h** (CCDC 843676), **2b** (CCDC 904924) and **3b** (CCDC 904923) have been deposited in the Cambridge Crystallographic Data Centre.

**General procedure for the synthesis of spiro[indoline-3,4’-pyridine] derivatives 1a–1p**: In an analogous manner to our procedure published in [25], a solution of arylamine (2.0 mmol), methyl propiolate (2.0 mmol) in 5 mL ethanol was stirred at room temperature overnight. Then isatin (2.0 mmol), malononitrile (2.0 mmol) and triethylamine (0.4 mmol) were added. The mixture was heated under reflux for about 24 hours. Then the solution was concentrated to approximately half the volume. The resulting precipitates were collected and washed with ethanol to give the pure product for analysis.

**General procedure for the synthesis of spiro[indoline-3,4’-pyridines] 2a–2h and spiro[indoline-3,4’-pyridinone] derivatives 3a–3n**: A similar procedure to that above was used. A solution of arylamine (2.0 mmol), methyl propiolate (2.0 mmol, 0.168 g) in 5 mL ethanol was stirred at room temperature overnight. Then isatin (2.0 mmol), ethyl cyanoacetate (2.0 mmol, 0.226 g) and triethylamine (0.4 mmol) were added to it, and the whole mixture was heated under reflux for about 24 hours. Then the solution was concentrated to approximately half the volume, which was subjected to column chromatography with ethyl acetate and light petroleum (v/v = 1:3) as eluent to give the pure product for analysis.

## Supporting Information

File 1 Experimental details and detailed spectroscopic data.
