# Supplementary material for: Synthesis of functionalized spiro[indoline-3,4’-pyridines] and spiro[indoline-3,4’-pyridinones] via one-pot four-component reactions
Source: Beilstein J Org Chem. 2013 May 2;9:846–51. doi: 10.3762/bjoc.9.97 (PMC3678513; doi:10.3762/bjoc.9.97)
Supplement: File 1 — Experimental details and detailed spectroscopic data. [file Beilstein_J_Org_Chem-09-846-s001.pdf]

Supporting Information

for

Synthesis of functionalized spiro[indoline-3,4'-pyridines] and spiro[indoline-3,4'-pyridinones] via one-pot four-component reactions

Li-Juan Zhang, Qun Wu, Jing Sun and Chao-Guo Yan*

Address: College of Chemistry & Chemical Engineering, Yangzhou University, Yangzhou 225002, China

Email: Chao-Guo Yan - cgyan@yzu.edu.cn

* Corresponding author

Experimental details and detailed spectroscopic data

1. General procedure for the synthesis of spiro[indoline-3,4'-pyridine] derivatives **1a–1p**: In an analogous manner to our procedure published in [1], a solution of arylamine (2.0 mmol), methyl propiolate (2.0 mmol) in 5 mL ethanol was stirred at room temperature overnight. Then isatin (2.0 mmol), malononitrile (2.0 mmol) and triethylamine (0.4 mmol) were added. The mixture was refluxed for about 24 hours. Then the solution was concentrated to half the volume. The resulting precipitates were collected and washed with ethanol to give the pure product for analysis.

1a: yellow solid, 79%, m.p. >250 ℃; ^{1}H NMR (600 MHz, DMSO-d_6) δ: 10.35 (s, 1H, NH), 7.57 (t, J = 7.2 Hz, 2H, ArH), 7.53~7.48 (m, 3H, ArH), 7.38 (s, 1H, CH), 7.24 (d, J = 7.2 Hz, 1H, ArH), 7.17 (t, J = 7.2 Hz, 1H, ArH), 6.96 (t, J = 7.8 Hz, 1H, ArH), 6.80 (d, J = 7.8 Hz, 1H, ArH), 5.80 (s, 2H, NH_2), 3.41 (s, 3H, OCH_3); ^{13}C NMR (150 MHz, DMSO-d_6) δ: 179.1, 164.7, 150.7, 141.3, 140.9, 138.8, 136.3, 130.2, 129.2, 128.3, 127.7, 123.9, 121.8, 119.0, 109.1, 104.1, 60.3, 51.1, 49.9; IR(KBr) υ: 3448, 3359, 3302, 3068, 2186, 1722, 1669, 1601, 1554, 1467, 1414, 1373, 1336, 1263, 1209, 1125, 1096, 1002, 942, 913, 761 cm^{-1}; MS (m/z): 373.31 ([M + 1]$^{+}$) 100%. Anal Calcd for $C_{21}H_{16}N_4O_3$: C 67.73, H 4.33, N 15.05; Found: C 67.48, H 4.53, N 14.71.

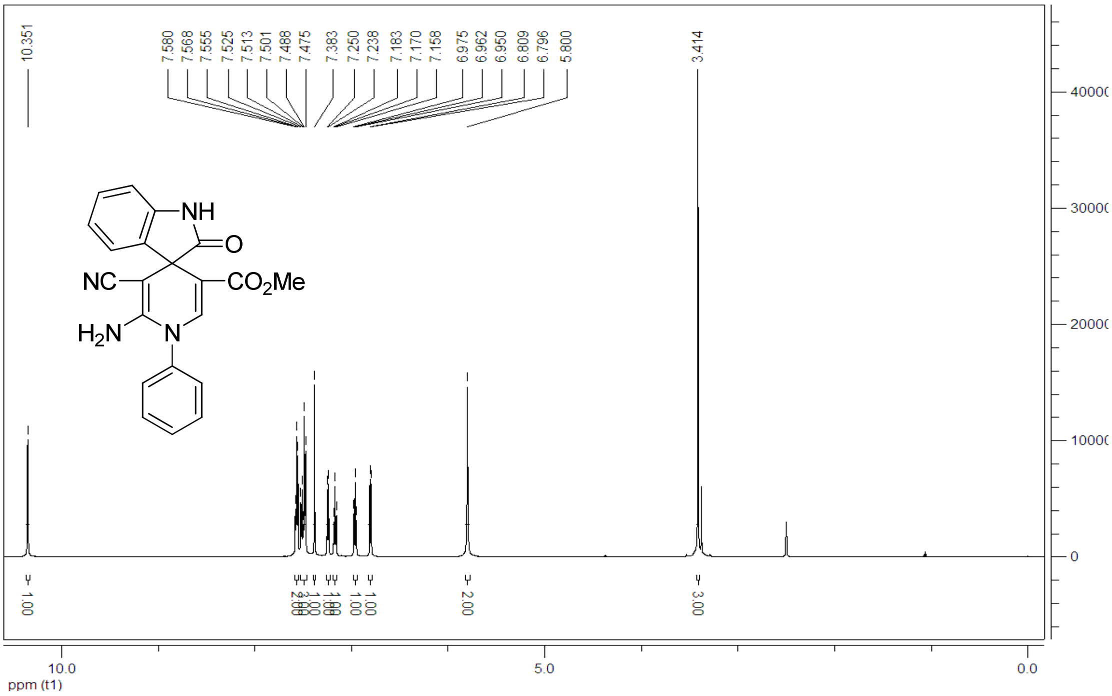

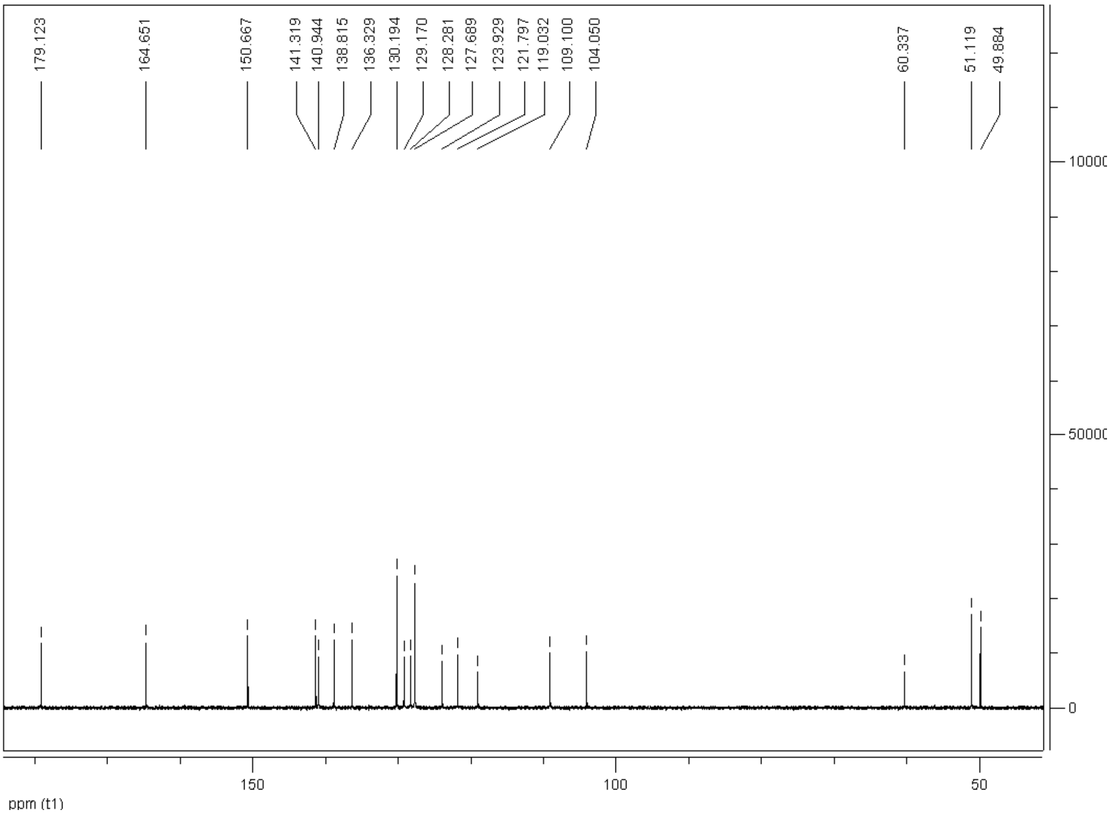

1b: white solid, 76%, m.p. >250 ℃; ^{1}H NMR (600 MHz, DMSO-d_6) δ: 10.33 (s, 1H, NH), 7.36~7.34 (m, 5H, ArH, CH), 7.23 (d, J = 7.2 Hz, 1H, ArH), 7.16 (t, J = 7.2 Hz, 1H, ArH), 6.95 (t, J = 7.2 Hz, 1H, ArH), 6.79 (d, J = 7.2 Hz, 1H, ArH), 5.74 (s, 2H, NH_2), 3.41 (s, 3H, OCH_3), 2.38 (s, 3H, CH_3); ^{13}C NMR (150 MHz, DMSO-d_6) δ: 179.1, 164.6, 150.7, 141.3, 141.0, 138.8, 136.4, 136.2, 130.6, 128.2, 127.5, 123.9, 121.7, 119.0, 109.0, 103.8, 60.1, 51.1, 49.8, 20.7; IR(KBr) υ: 3581, 3330, 3193, 3078, 2189, 1713, 1662, 1613, 1556, 1508, 1465, 1423, 1330, 1237, 1180, 1116, 1036, 936, 839, 755 cm^{-1}; MS (m/z): 387.50 ([M + 1]$^{+}$) 100%. Anal Calcd for $C_{22}H_{18}N_4O_3$: C 68.38, H 4.70, N 14.50; Found: C 68.22, H 5.14, N 14.19.

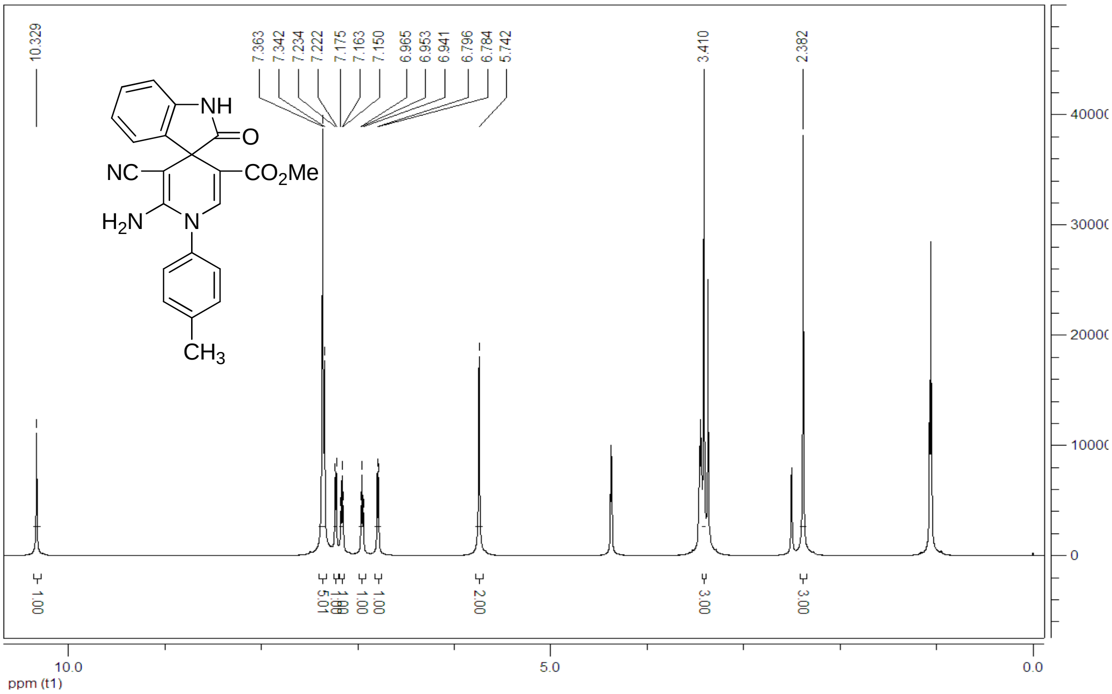

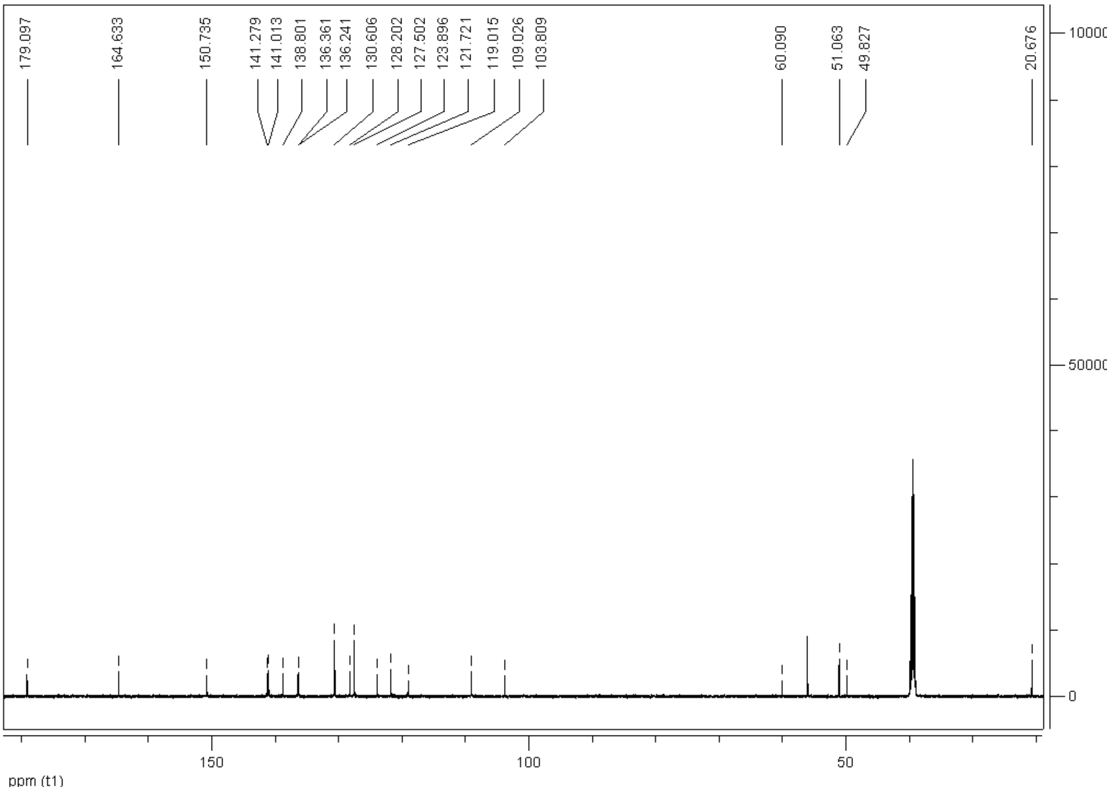

1c: yellow solid, 74%, m.p. 200–201 ℃; ^{1}H NMR (600 MHz, DMSO-d_6) δ: 10.31 (s, 1H, NH), 7.41 (d, J = 9.0 Hz, 2H, ArH), 7.33 (s, 1H, CH), 7.24 (d, J = 7.8 Hz, 1H, ArH), 7.16 (t, J = 7.8 Hz, 1H, ArH), 7.09 (d, J = 9.0 Hz, 2H, ArH), 6.95 (t, J = 7.8 Hz, 1H, ArH), 6.78 (d, J = 7.8 Hz, 1H, ArH), 5.74 (s, 2H, NH_2), 3.82 (s, 3H, OCH_3), 3.41 (s, 3H, OCH_3); ^{13}C NMR (150 MHz, DMSO-d_6) δ: 179.1, 164.6, 159.5, 151.0, 141.3, 136.4, 131.3, 129.2, 128.2, 123.9, 121.7, 119.1, 115.2, 109.0, 103.6, 59.8, 55.5, 51.0, 49.8; IR(KBr) υ: 3345, 2961, 2193, 1718, 1665, 1618, 1553, 1510, 1468, 1430, 1370, 1336, 1287, 1250, 1182, 1114, 1040, 936, 851, 765 cm^{-1}; MS (m/z): 403.48 ($[M + 1]^+$) 100%. Anal Calcd for $C_{22}H_{18}N_4O_4$: C 65.66, H 4.51, N 13.92; Found: C 65.37, H 4.77, N 13.63.

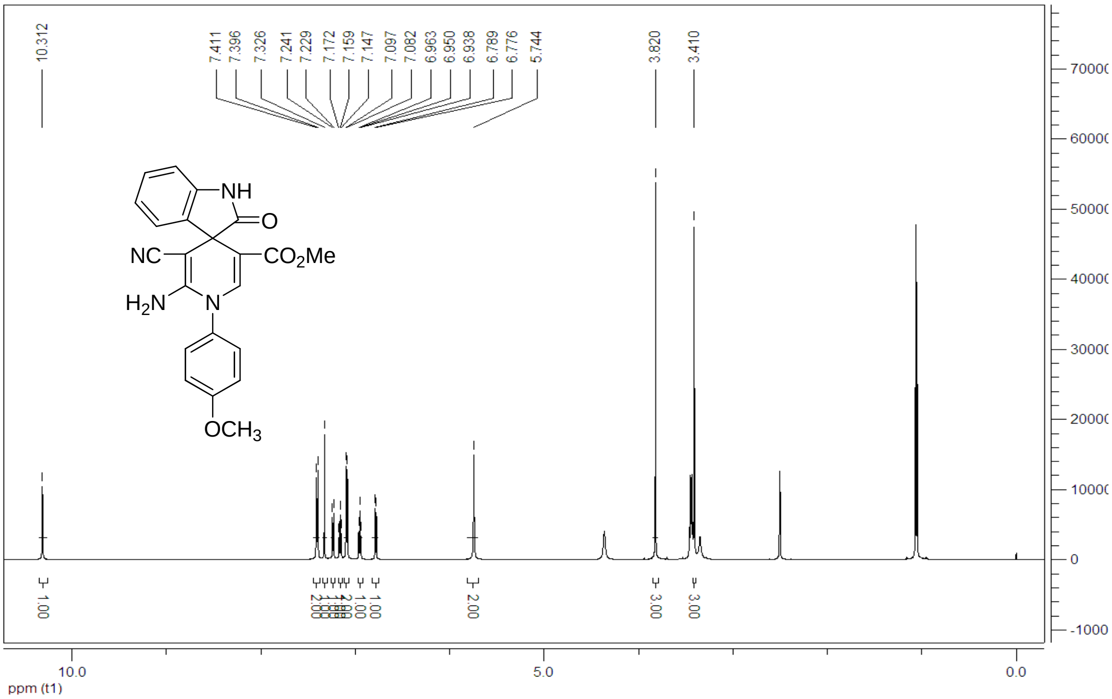

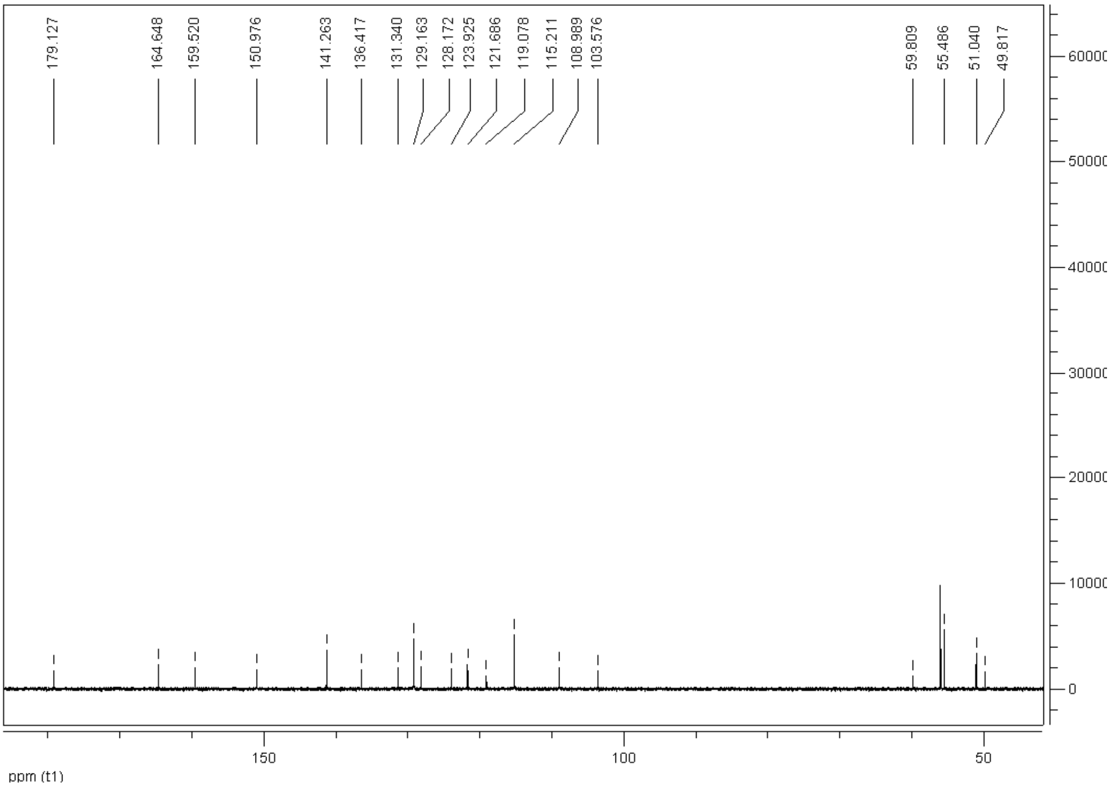

1d: yellow solid, 81%, m.p. >250 ℃; ^{1}H NMR (600 MHz, DMSO-d_6) δ: 10.34 (s, 1H, NH), 7.60 (d, J = 7.8 Hz, 2H, ArH), 7.51 (d, J = 7.8 Hz, 2H, ArH), 7.39 (s, 1H, CH), 7.24 (d, J = 7.2 Hz, 1H, ArH), 7.16 (t, J = 7.2 Hz, 1H, ArH), 6.95 (t, J = 7.2 Hz, 1H, ArH), 6.79 (d, J = 7.8 Hz, 1H, ArH), 5.94 (s, 2H, NH_2), 3.42 (s, 3H, OCH_3); ^{13}C NMR (150 MHz, DMSO-d_6) δ: 179.0, 164.6, 150.6, 141.3, 140.8, 137.7, 136.2, 133.6, 130.0, 129.8, 128.2, 123.9, 121.7, 118.9, 109.0, 104.2, 60.2, 51.1, 49.8; IR(KBr) υ: 3372, 3177, 2964, 2185, 1719, 1693, 1664, 1617, 1551, 1484, 1431, 1396, 1327, 1251, 1181, 1115, 1089, 1047, 931, 890, 847 cm^{-1}; MS (m/z): 407.05 ([M + 1]$^{+}$) 100%. Anal Calcd for $C_{21}H_{15}ClN_4O_3$: C 62.00, H 3.72, N 13.77; Found: C 61.76, H 3.74, N 13.64.

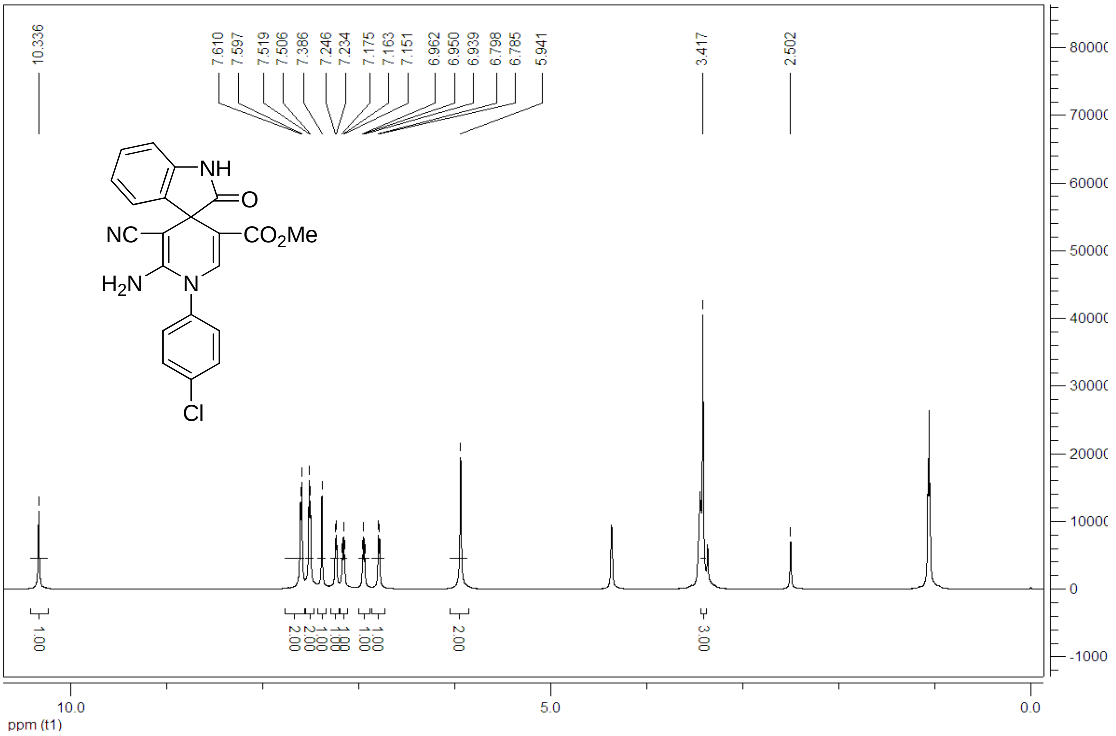

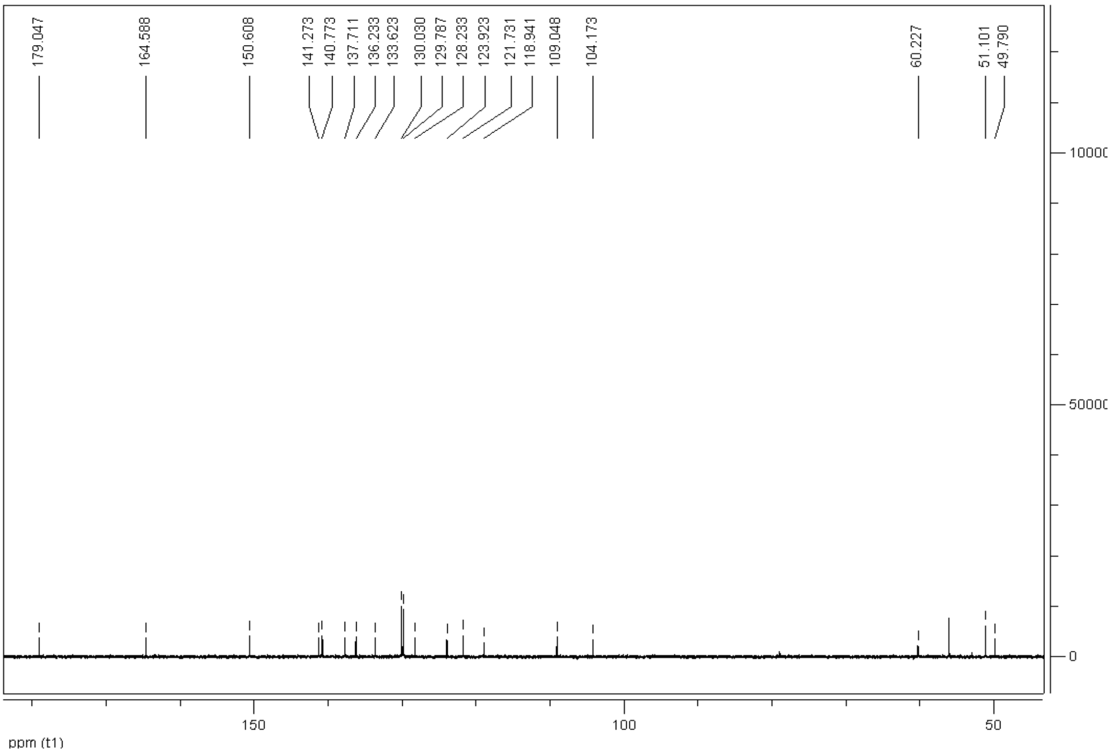

1e: yellow solid, 80%, m.p. 180–181 ℃; ^{1}H NMR (600 MHz, DMSO-d_6) δ: 10.30 (s, 1H, NH), 7.73 (d, J = 8.4 Hz, 2H, ArH), 7.44 (d, J = 8.4 Hz, 2H, ArH), 7.38 (s, 1H, CH), 7.23 (d, J = 7.2 Hz, 1H, ArH), 7.16 (t, J = 7.2 Hz, 1H, ArH), 6.95 (t, J = 7.2 Hz, 1H, ArH), 6.79 (d, J = 7.8 Hz, 1H, ArH), 5.89 (s, 2H, NH_2), 3.42 (s, 3H, OCH_3); ^{13}C NMR (150 MHz, DMSO-d_6) δ: 179.0, 164.6, 150.6, 141.3, 140.7, 138.2, 136.2, 133.0, 130.1, 128.2, 123.9, 122.2, 121.7, 118.9, 109.0, 104.2, 60.3, 51.1, 49.8; IR(KBr) υ: 3441, 3351, 2960, 2188, 1708, 1671, 1619, 1558, 1477, 1427, 1374, 1334, 1255, 1218, 1185, 1116, 1010, 930, 890, 839 cm^{-1}; MS (m/z): 449.17 ([M - 1]$^{+}$) 100%. Anal Calcd for $C_{21}H_{15}BrN_4O_3$: C 55.89, H 3.35, N 12.42; Found: C 55.77, H 3.72, N 12.20.

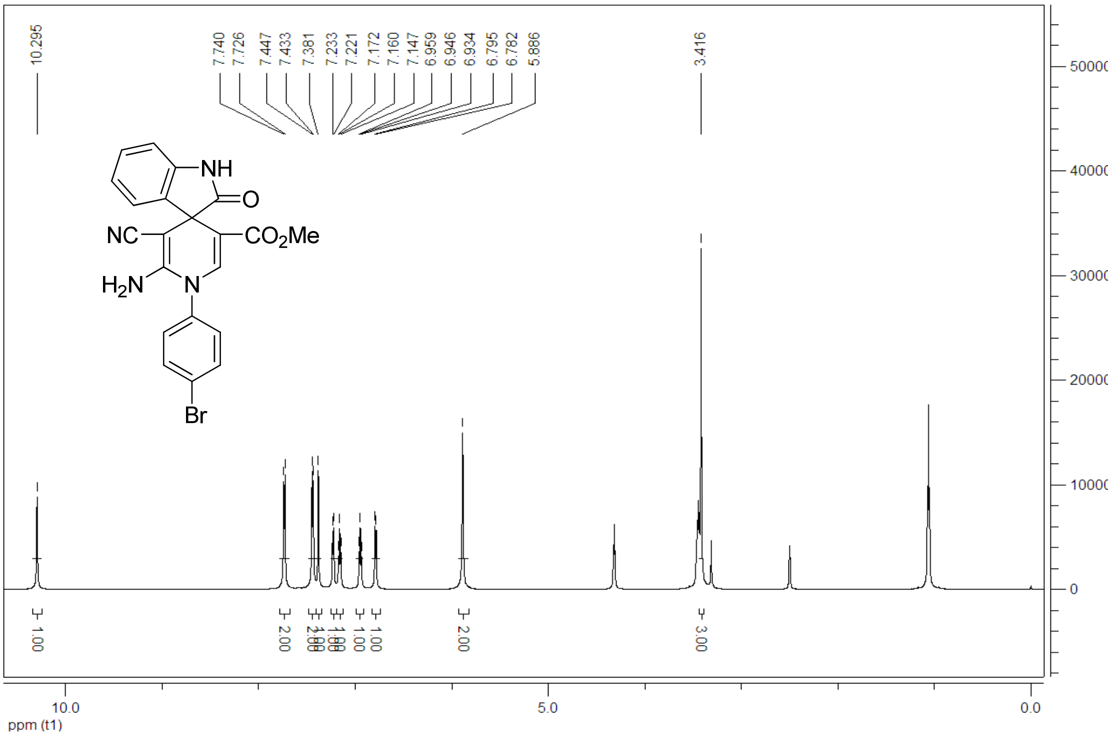

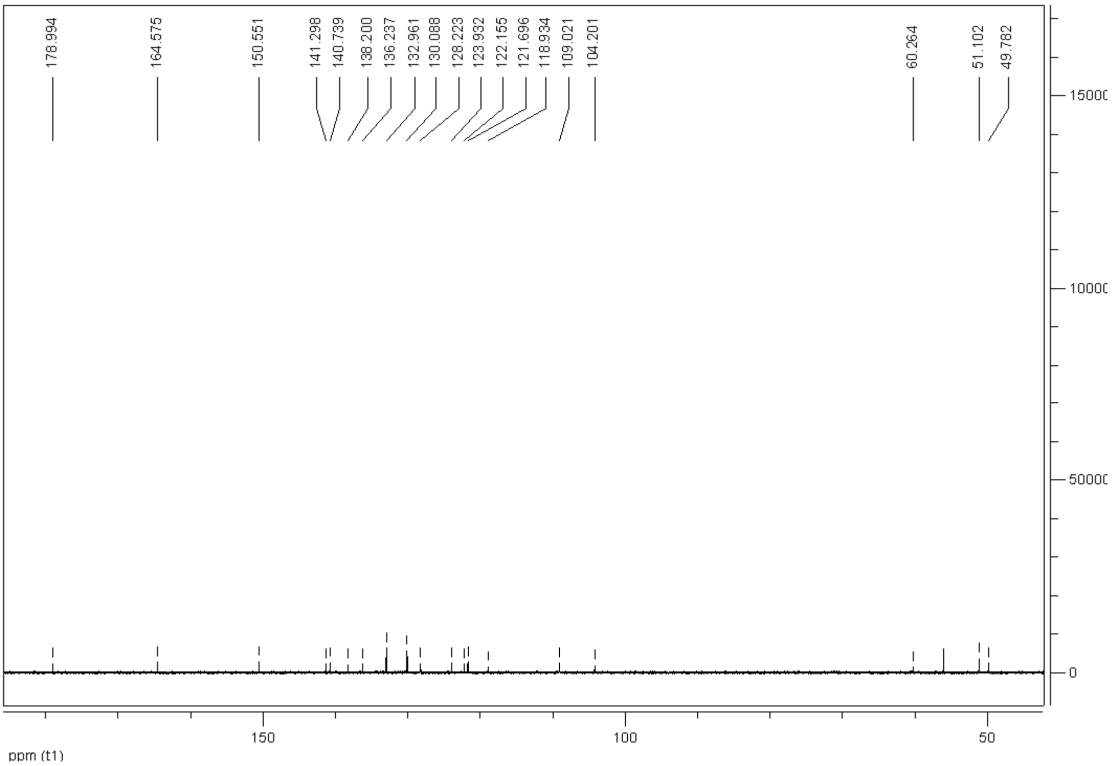

1f: yellow solid, 75%, m.p. >250 ℃; ^{1}H NMR (600 MHz, DMSO-d_6) δ: 10.18 (s, 1H, NH), 7.60 (d, J = 8.4 Hz, 2H, ArH), 7.51 (d, J = 7.8 Hz, 2H, ArH), 7.37 (s, 1H, CH), 7.04 (brs, 1H, ArH), 6.96 (d, J = 7.8 Hz, 1H, ArH), 6.67 (d, J = 7.2 Hz, 1H, ArH), 5.86 (s, 2H, NH_2), 3.42 (s, 3H, OCH_3), 2.26 (s, 1H, CH_3) ; ^{13}C NMR (150 MHz, DMSO-d_6) δ: 179.0, 164.6, 150.5, 140.7, 138.9, 137.8, 136.4, 133.6, 130.5, 130.0, 129.8, 128.5, 124.5, 119.0, 108.7, 104.2, 60.4, 51.1, 49.8, 20.6; IR(KBr) υ: 3373, 3174, 2964, 2187, 1720, 1669, 1554, 1492, 1430, 1399, 1371, 1333, 1285, 1248, 1212, 1141, 1097, 1049, 1016, 958, 886, 841, 813, 790, 767 cm^{-1}; MS (m/z): 419.55 ($[M - 1]^{+}$) 100%. Anal Calcd for $C_{22}H_{17}ClN_4O_3$: C 62.79, H 4.07, N 13.31; Found: C 62.42, H 4.34, N 13.25.

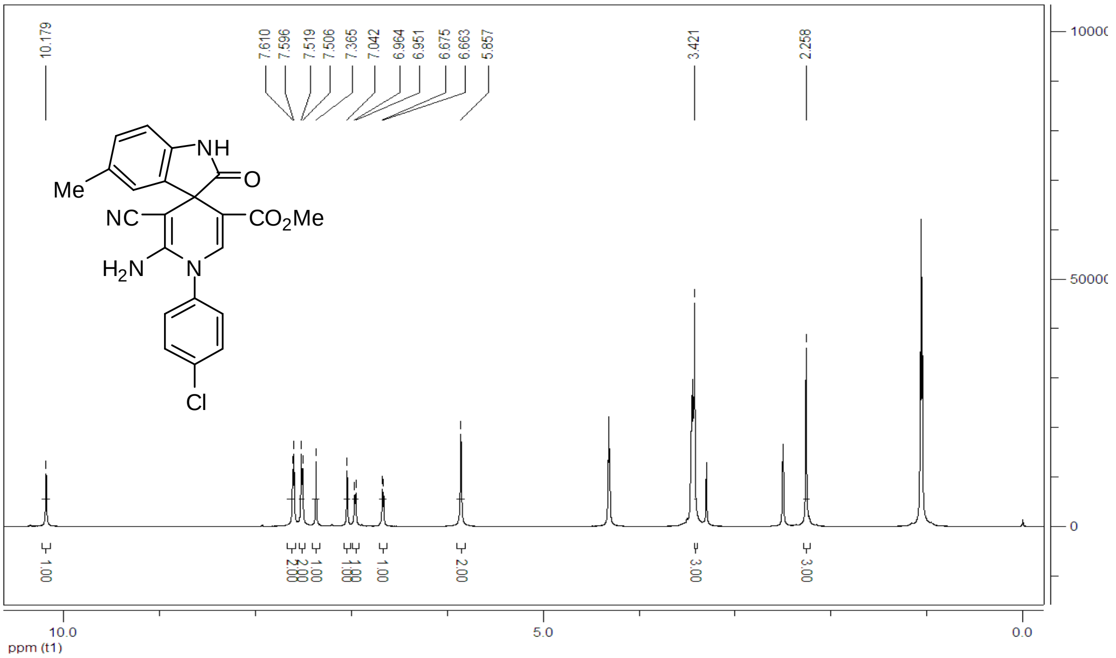

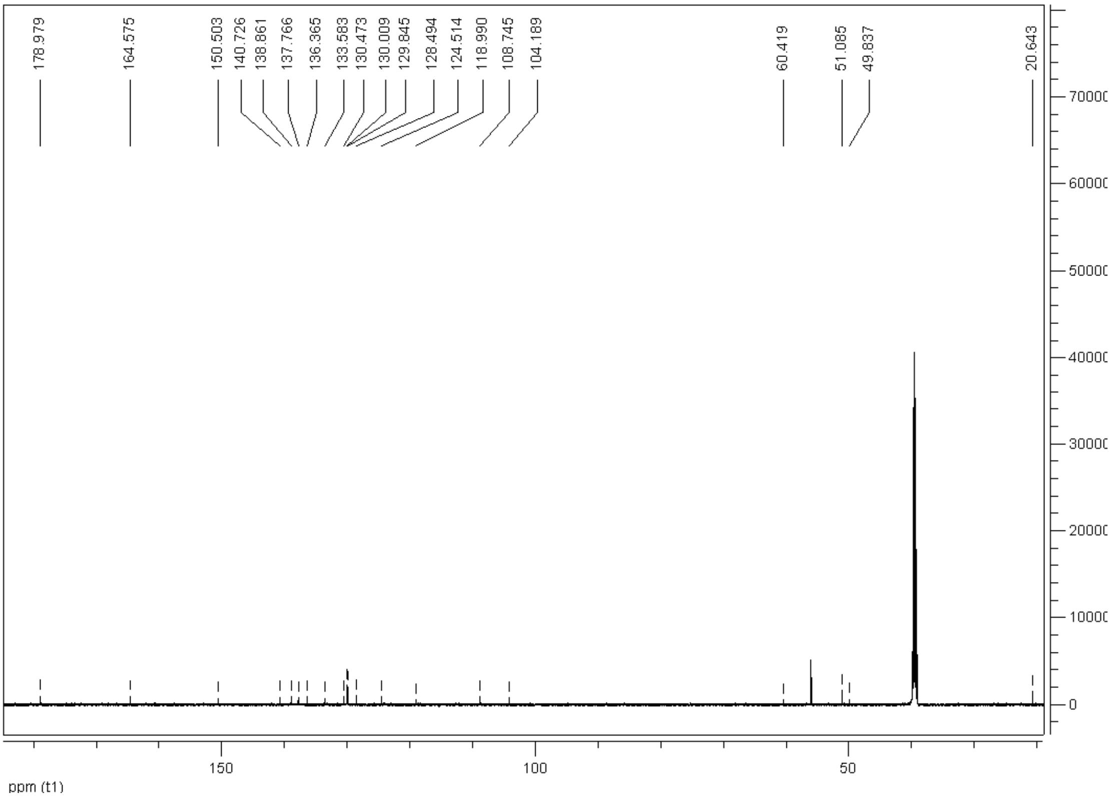

1g: yellow solid, 73%, m.p. >250 ℃; ^{1}H NMR (600 MHz, DMSO-d_6) δ: 10.20 (s, 1H, NH), 7.37–7.33 (m, 5H, ArH), 7.04 (s, 1H, CH), 6.96 (d, J = 7.8 Hz, 1H, ArH), 6.67 (d, J = 7.8 Hz, 1H, ArH), 5.50 (s, 2H, NH_2), 3.41 (s, 3H, OCH_3), 2.39 (s, 3H, CH_3), 2.26 (s, 3H, CH_3); ^{13}C NMR (150 MHz, DMSO-d_6) δ: 179.1, 164.6, 150.6, 141.0, 138.8, 138.7, 136.5, 136.3, 130.6, 130.5, 128.5, 127.6, 124.5, 119.1, 108.7, 103.8, 56.0, 51.1, 49.9, 20.7, 20.6; IR(KBr) υ: 3462, 3325, 2941, 2184, 1725, 1683, 1627, 1562, 1492, 1421, 1375, 1341, 1274, 1213, 1142, 1103, 1041, 1009, 942, 905, 853, 830, 801, 761 cm^{-1}; MS (m/z): 399.32 ($[M - 1]^+$) 100%. Anal Calcd for $C_{23}H_{20}N_4O_3$: C 68.99, H 5.03, N 13.99; Found: C 68.80, H 5.42, N 13.67.

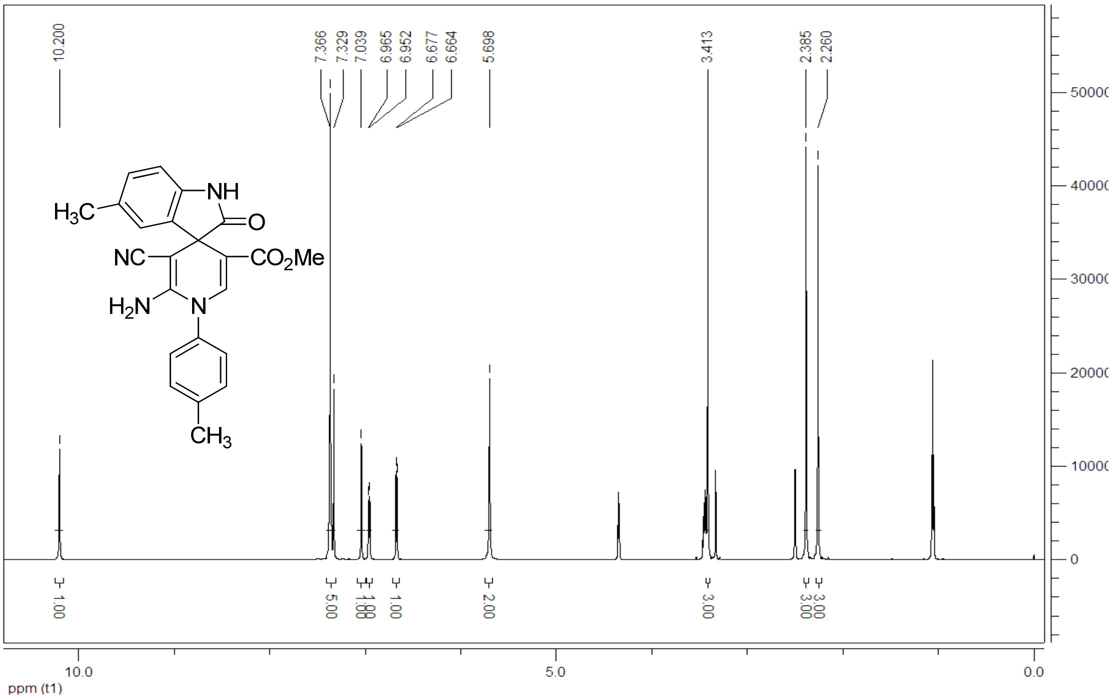

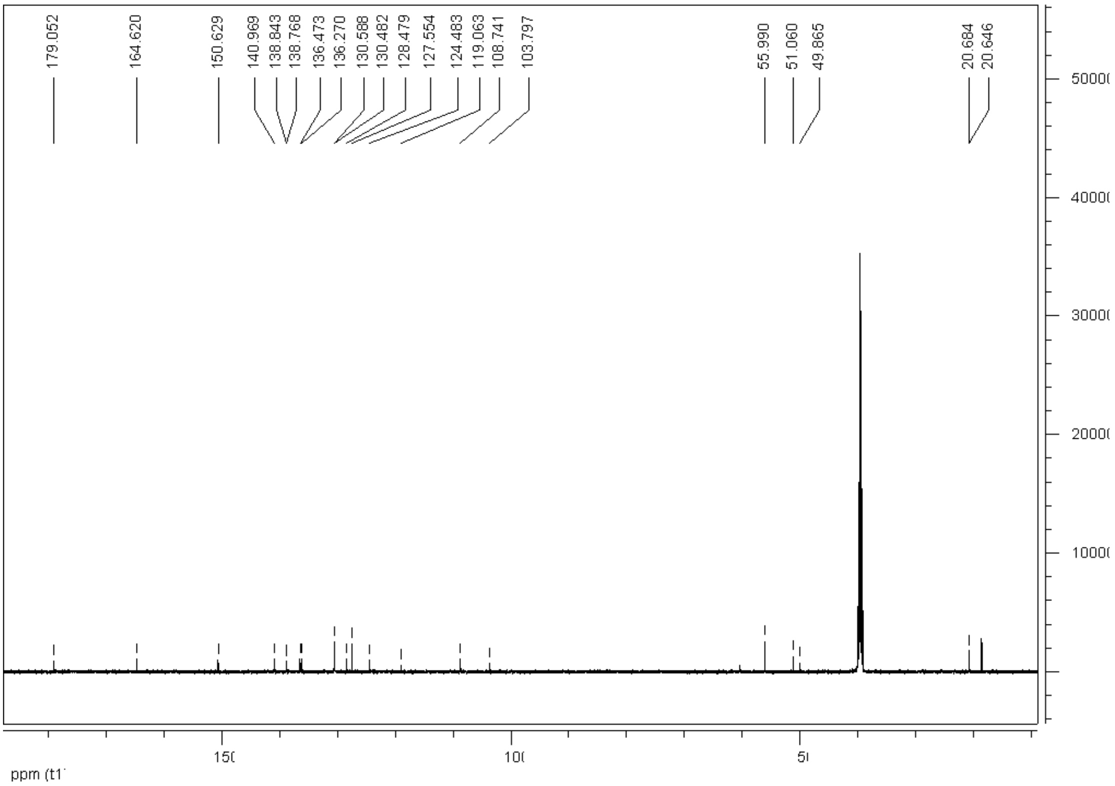

1h: white solid, 84%, m.p. >250 ℃; ^{1}H NMR (600 MHz, DMSO-d_6) δ: 10.43 (s, 1H, NH), 7.60 (d, J = 8.4 Hz, 2H, ArH), 7.55 (d, J = 8.4 Hz, 2H, ArH), 7.38–7.36 (m, 2H, CH, ArH), 7.21 (d, J = 7.8 Hz, 1H, ArH), 6.80 (d, J = 8.4 Hz, 1H, ArH), 5.97 (s, 2H, NH_2), 3.44 (s, 3H, OCH_3); ^{13}C NMR (150 MHz, DMSO-d_6) δ: 178.9, 164.6, 150.8, 141.2, 140.3, 138.2, 137.6, 133.7, 130.0, 129.9, 128.2, 125.7, 124.2, 118.8, 110.4, 103.2, 59.4, 51.2, 50.2; IR(KBr) υ: 3455, 3326, 3223, 3094, 2950, 2184, 1748, 1683, 1626, 1562, 1482, 1428, 1376, 1343, 1272, 1221, 1180, 1127, 1097, 1042, 1010, 939, 882, 847, 813, 770 cm^{-1}; MS (m/z): 439.23 ($[M - 1]^{+}$) 100%. Anal Calcd for $C_{21}H_{14}Cl_2N_4O_3$: C 57.16, H 3.20, N 12.70; Found: C 57.31, H 3.56, N 12.48.

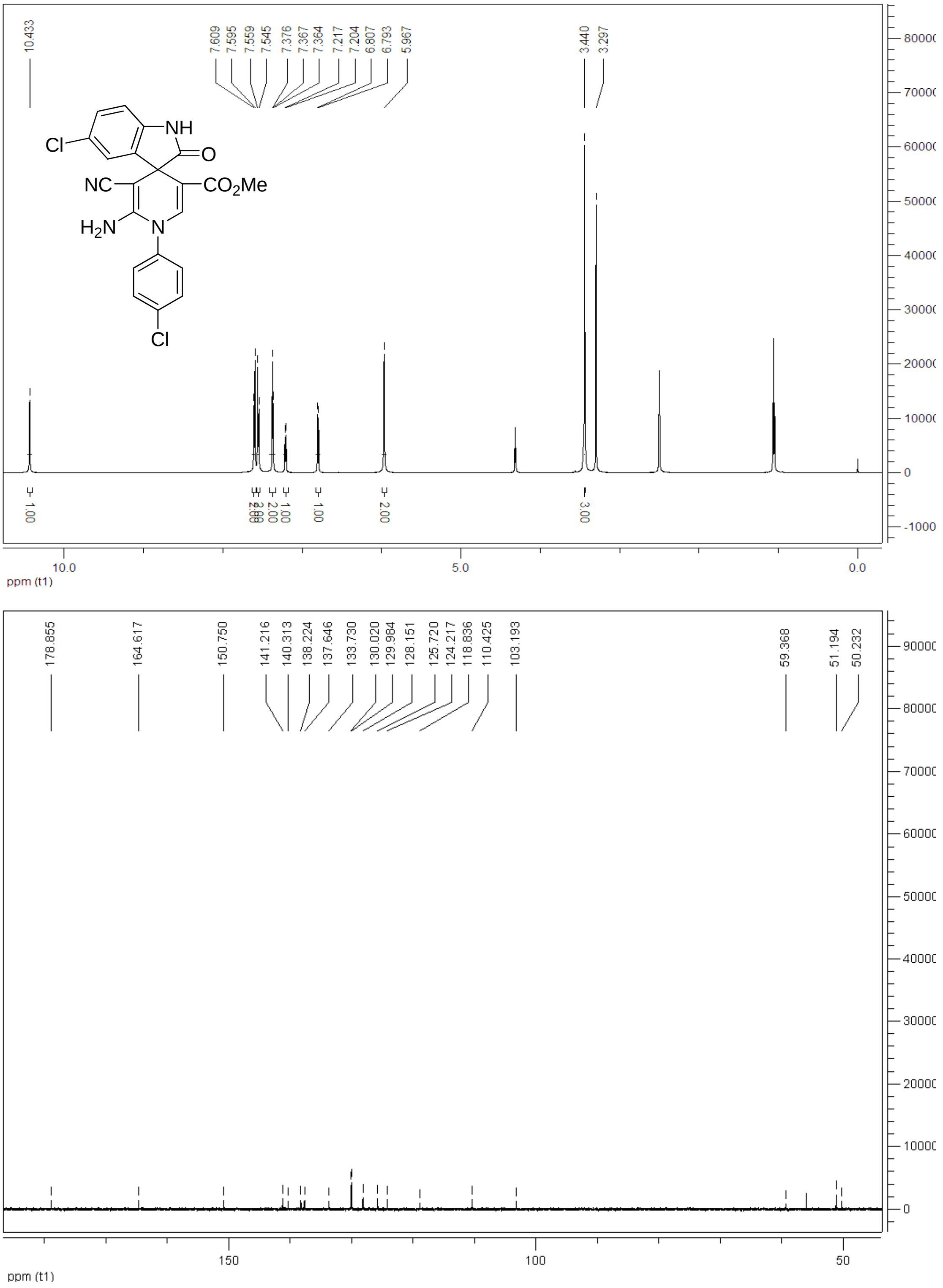

1i: yellow solid, 72%, m.p. >250 ℃; ^{1}H NMR (600 MHz, DMSO-d_6) δ: 10.47 (s, 1H, NH), 7.41–7.35 (m, 6H, ArH), 7.21 (d, J = 8.4 Hz, 1H, ArH), 6.81 (d, J = 8.4 Hz, 1H, ArH), 5.82 (s, 2H, NH_2), 3.44 (s, 3H, OCH_3), 2.38 (s, 3H, CH_3); ^{13}C NMR (150 MHz, DMSO-d_6) δ: 178.9, 164.7, 150.9, 141.5, 140.3, 138.9, 138.3, 136.2, 130.6, 128.1, 127.7, 125.7, 124.2, 118.9, 110.4, 102.8, 56.0, 51.2, 50.3, 20.7; IR(KBr) υ: 3504, 3458, 3326, 3220, 2946, 2184, 1748, 1677, 1625, 1561, 1512, 1477, 1424, 1375, 1343, 1279, 1253, 1218, 1181, 1127, 1041, 1010, 941, 881, 810, 766 cm^{-1}; MS (m/z): 421.71 ($[M + 1]^+$) 100%. Anal Calcd for $C_{22}H_{17}ClN_4O_3$: C 62.79, H 4.07, N 13.31; Found: C 62.56, H 4.25, N 12.94.

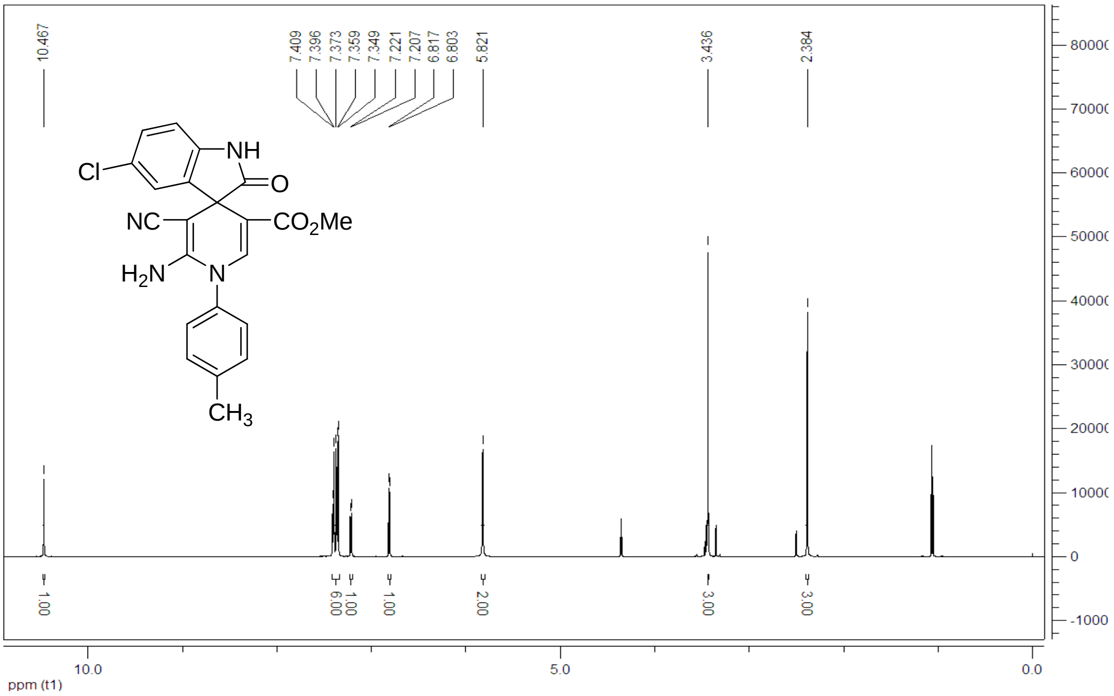

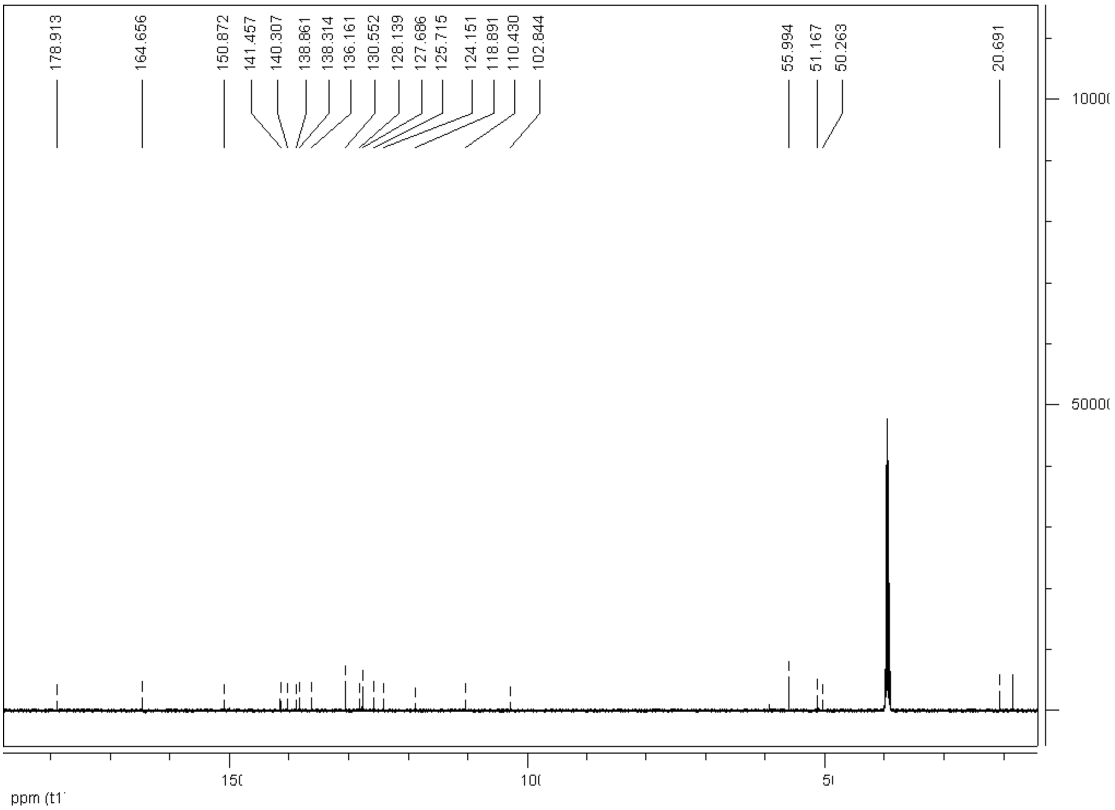

1j: yellow solid, 64%, m.p. 222–224 ℃; ^{1}H NMR (600 MHz, DMSO-d_6) δ: 7.62–7.48 (m, 6H, ArH), 7.44 (s, 1H, CH), 7.35–7.25 (m, 4H, ArH), 7.16 (t, J = 7.8 Hz, 1H, ArH), 7.02 (t, J = 7.8 Hz, 1H, ArH), 6.73 (d, J = 7.8Hz, 1H, ArH), 6.03 (s, 2H, NH_2), 4.99, 4.84 (dd, J = 16.2 Hz, 2H), (m, 2H, CH_2), 3.37 (s, 3H, OCH_3); ^{13}C NMR (150 MHz, DMSO-d_6) δ: 177.7, 164.7, 150.9, 141.9, 141.1, 137.7, 136.2, 135.5, 133.7, 130.1, 129.9, 128.4, 127.3, 127.2, 123.9, 122.6, 119.1, 108.7, 104.0, 59.9, 51.2, 49.6, 43.4; IR(KBr) υ: 3450, 3320, 3172, 3073, 2945, 2178, 1706, 1669, 1609, 1564, 1490, 1462, 1428, 1337, 1291, 1253, 1212, 1177, 1117, 1092, 1040, 1012, 988, 927, 839, 795, 760 cm^{-1}; MS (*m/z*): 495.14 ([M - 1]$^{+}$) 100%. Anal Calcd for $C_{28}H_{21}ClN_4O_3$: C 67.67, H 4.26, N 11.27; Found: C 67.52, H 4.51, N 11.25.

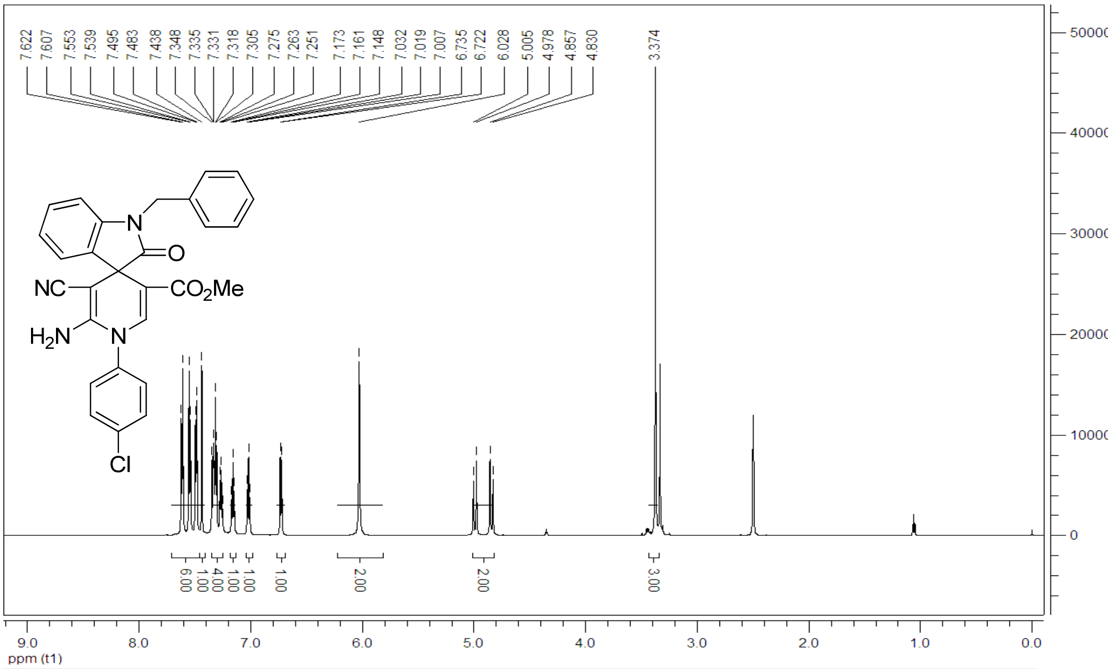

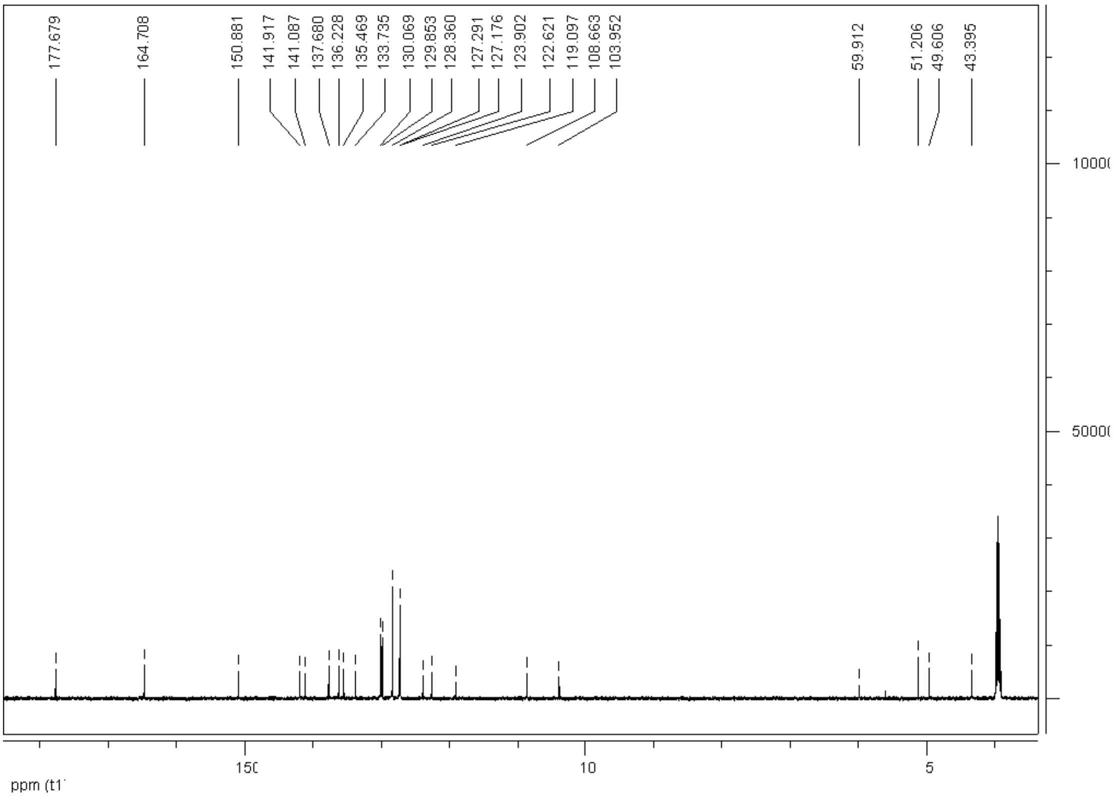

1k: yellow solid, 75%, m.p. 132–134 ℃; ^{1}H NMR (600 MHz, DMSO-d_6) δ: 7.49 (d, J = 7.2 Hz, 2H, ArH), 7.40–7.37 (m, 5H, ArH), 7.32 (t, J = 7.8 Hz, 3H, ArH), 7.26 (t, J = 7.2 Hz, 1H, ArH), 7.16 (t, J = 7.2 Hz, 1H, ArH), 7.02 (t, J = 7.8 Hz, 1H, ArH), 6.73 (d, J = 7.8 Hz, 1H, ArH), 5.84 (s, 2H, NH), 4.99, 4.84 (dd, J = 16.0 Hz, 2H, CH), 3.37 (s, 3H, OCH_3), 2.39 (s, 3H, CH_3); ^{13}C NMR (150 MHz, DMSO-d_6) δ: 177.7, 164.7, 151.0, 141.8, 141.3, 138.9, 136.2, 136.1, 135.5, 130.6, 128.3, 127.6, 127.3, 123.8, 122.6, 119.1, 108.6, 103.5, 59.7, 56.0, 51.2, 49.6, 43.3, 20.7, 18.5; IR(KBr) υ: 3465, 3365, 3214, 3036, 2960, 2183, 1709, 1662, 1610, 1553, 1431, 1353, 1242, 1185, 1114, 1037, 933, 838, 744 cm^{-1}; MS (m/z): HRMS (ESI) Calcd. for $C_{29}H_{23}N_4O_3$ ($[M - H]^-$): 475.1778, found: 475.1779.

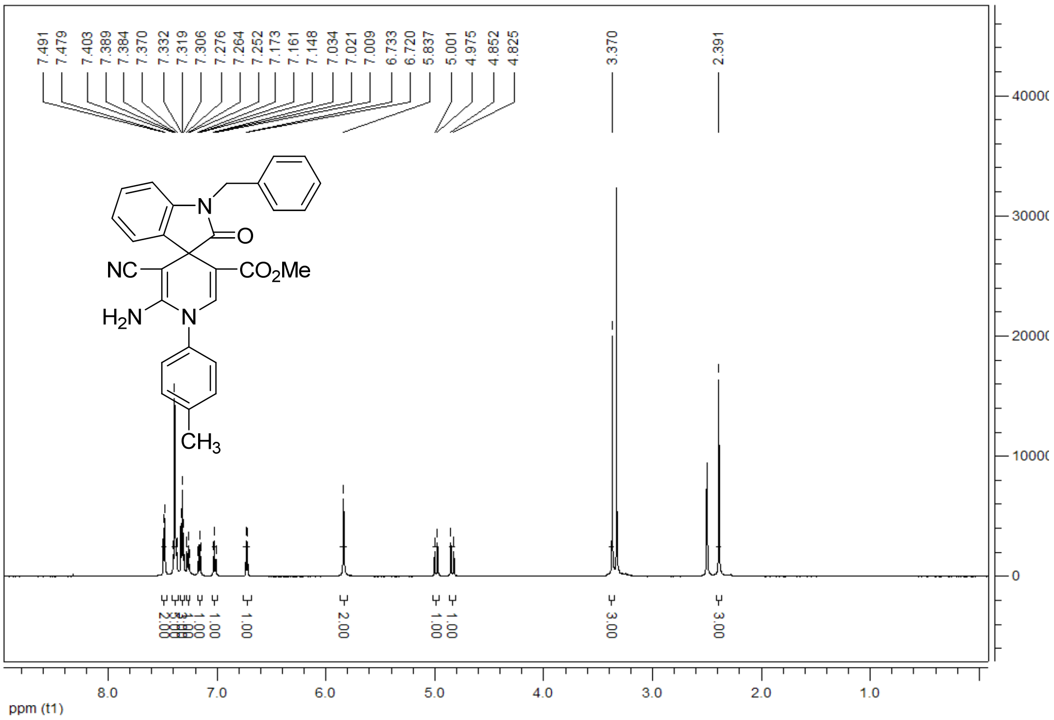

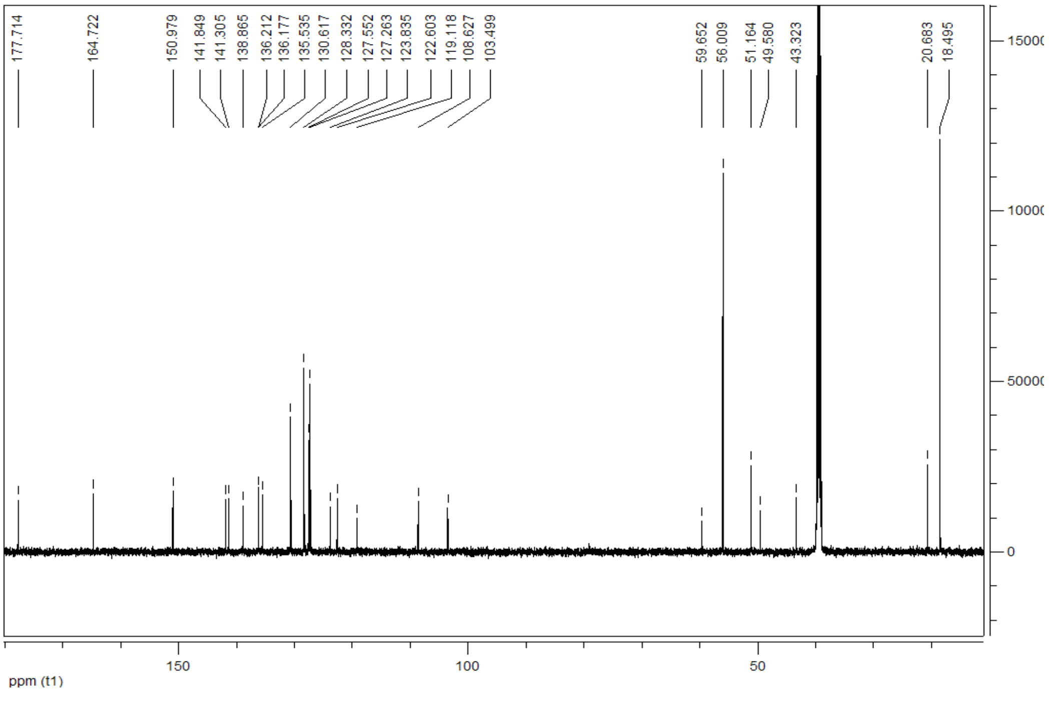

1l: yellow solid, 72%, m.p. 130–132 ℃; ^{1}H NMR (600 MHz, DMSO-d_6) δ: 7.48 (d, J = 7.8 Hz, 2H, ArH), 7.44 (d, J = 7.2 Hz, 2H, ArH), 7.38 (s, 1H, ArH), 7.34–7.31 (m, 3H, ArH), 7.26 (t, J = 7.2 Hz, 1H, ArH), 7.16 (t, J = 7.8 Hz, 1H, ArH), 7.10 (d, J = 9.0 Hz, 2H, ArH), 7.02 (t, J = 7.2 Hz, 1H, ArH), 6.72 (d, J = 7.8 Hz, 1H, ArH), 5.82 (s, 2H, NH_2), 4.99 (d, J = 16.0 Hz , 1H, CH), 4.84 (d, J = 16.0 Hz, 1H, CH), 3.83 (s, 3H, CH_3), 3.37 (s, 3H, CH_3); ^{13}C NMR (150 MHz, DMSO-d_6) δ: 177.8, 164.7, 159.6, 151.2, 141.8, 141.5, 136.2, 135.6, 131.3, 129.2, 128.3, 128.2, 127.3, 127.1, 123.9, 122.6, 119.2, 115.2, 108.6, 103.3, 59.4, 56.0, 55.5, 51.1, 49.6, 43.3, 18.5; IR(KBr) υ: 3450, 3364, 2946, 2183, 1708, 1666, 1610, 1560, 1509, 1427, 1343, 1246, 1179, 1111, 1035, 927, 844, 755 cm^{-1}; MS (m/z): HRMS (ESI) Calcd. for $C_{29}H_{23}N_4O_4$ ($[M - H]^-$): 491.1723, found: 491.1724.

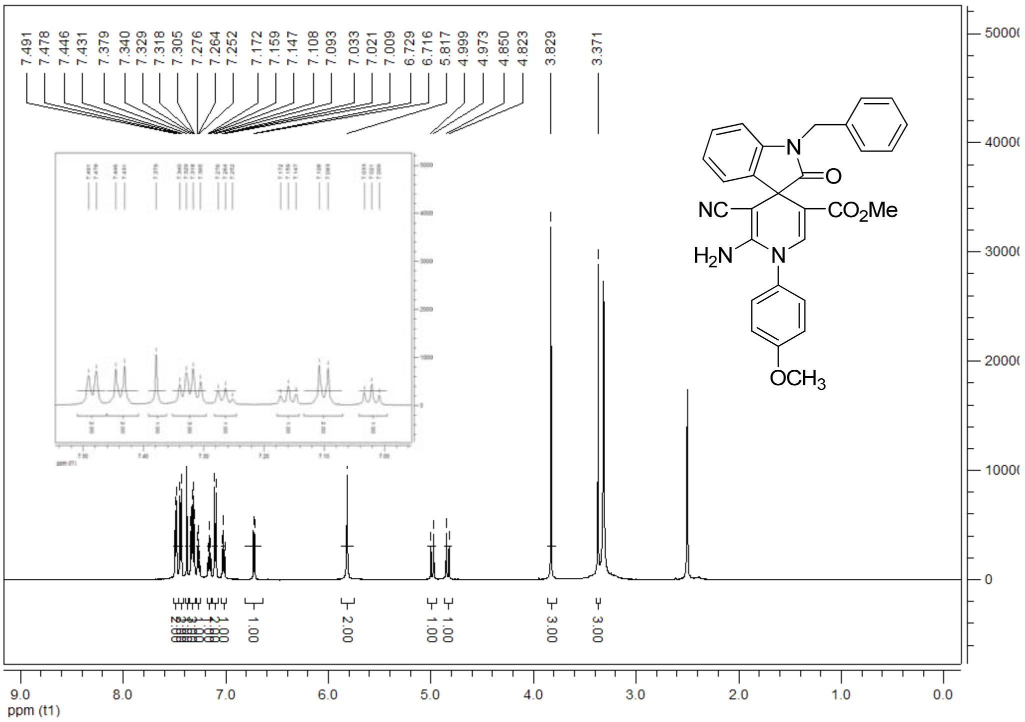

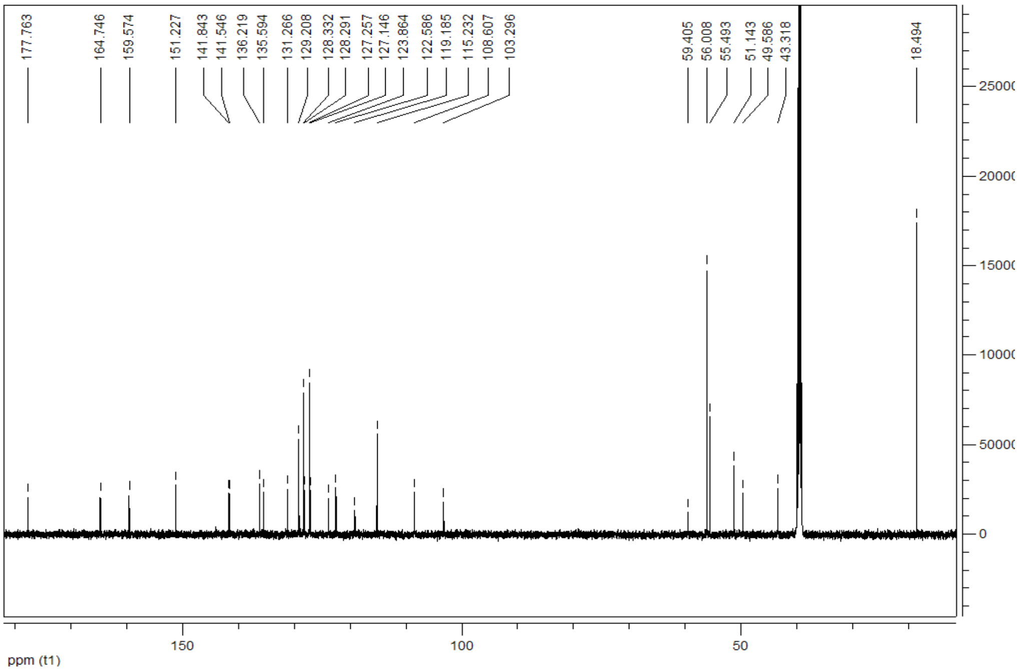

1m: yellow solid, 66%, m.p. 226–228 ℃; ^{1}H NMR (600 MHz, DMSO-d_6) δ: 7.58–7.43 (m, 8H, ArH), 7.32–7.27 (m, 4H, ArH), 7.17 (s, 1H, ArH), 7.03 (s, 1H, ArH), 6.73 (d, J = 5.4 Hz, 1H, ArH), 5.89 (s, 2H, NH_2), 4.99 (d, J = 15.6 Hz, 1H, CH), 4.84 (d, J = 15.6 Hz, 1H, CH), 3.38 (s, 3H, CH_3); ^{13}C NMR (150 MHz, DMSO-d_6) δ: 177.7, 164.7, 150.8, 141.9, 141.2, 138.8, 136.2, 135.5, 130.2, 129.2, 128.3, 127.8, 127.3, 127.2, 123.8, 122.6, 119.1, 106.6, 103.7, 59.9, 56.0, 51.2, 49.6, 43.3, 18.5; IR(KBr) υ: 3440, 3331, 3223, 3057, 2954, 2184, 1714, 1663, 1606, 1560, 1489, 1424, 1340, 1255, 1201, 1172, 1117, 995, 922, 845, 752, 703 cm^{-1}; MS (m/z): HRMS (ESI) Calcd. for $C_{28}H_{21}N_4O_3$ ([M - H]$^-$): 461.1598, found: 461.1598.

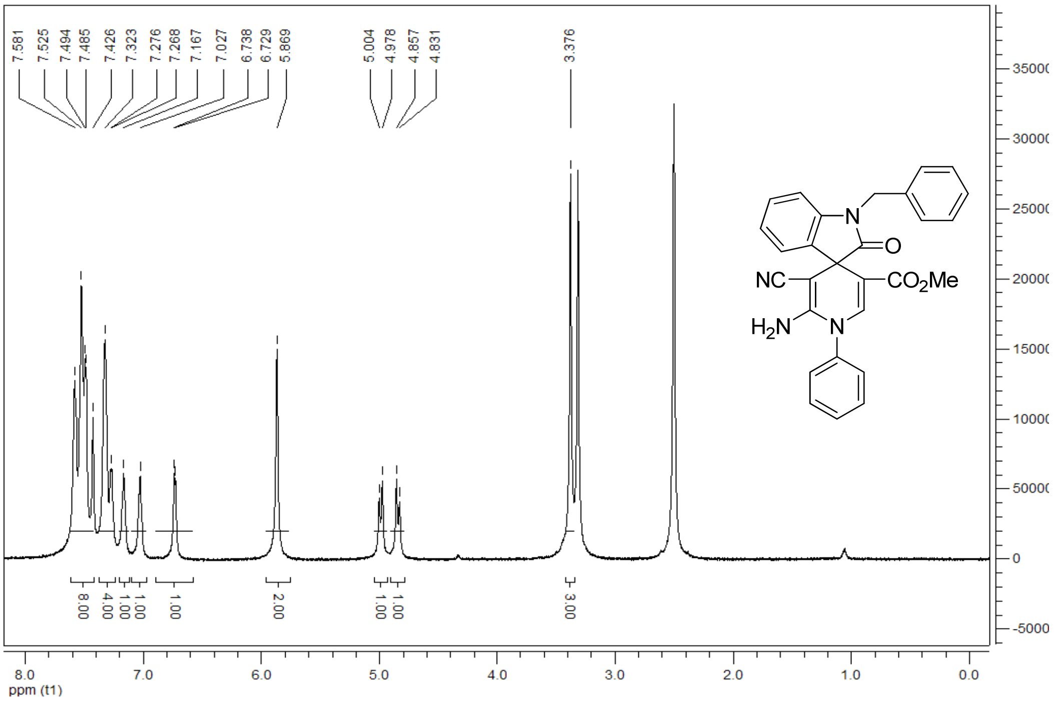

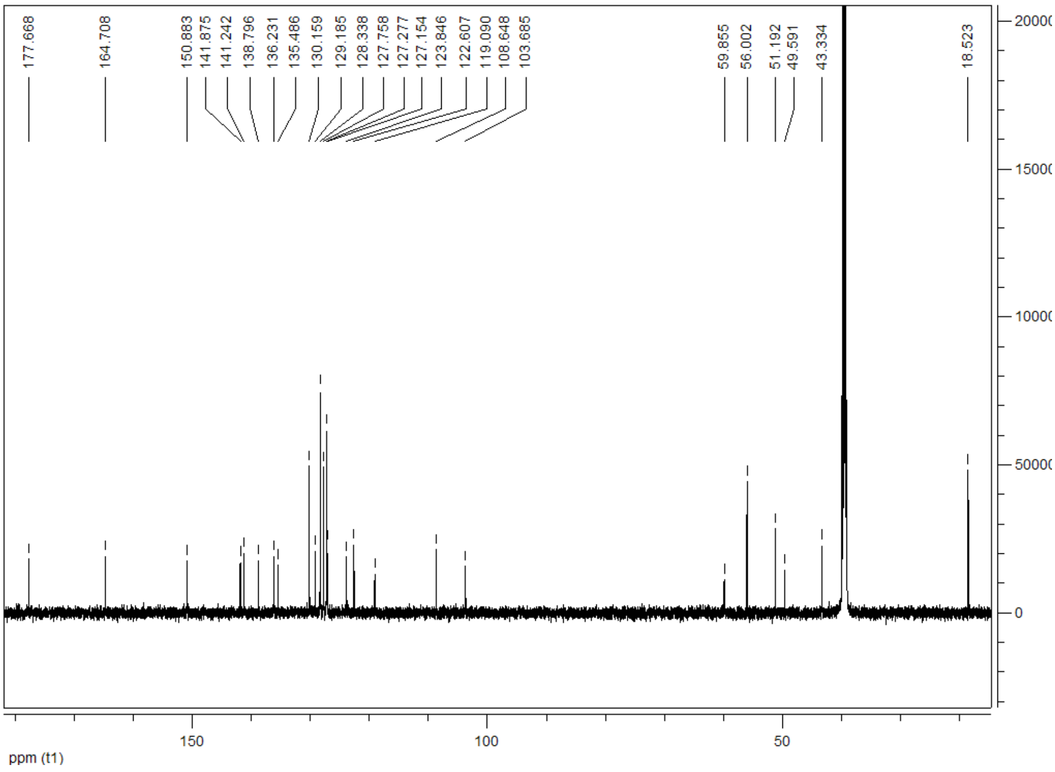

1n: yellow solid, 61%, m.p. >250 ℃; ^{1}H NMR (600 MHz, DMSO-d_6) δ: 7.71 (s, 1H, ArH), 7.58–7.56 (m, 2H, ArH), 7.49 (d, J = 7.2 Hz, 3H, ArH), 7.46 (s, 1H, ArH), 7.38 (d, J = 7.2 Hz, 1H, ArH), 7.32 (t, J = 7.2 Hz, 2H, ArH), 7.27 (t, J = 6.6 Hz, 1H, ArH), 7.16 (t, J = 6.6 Hz, 1H, ArH), 7.02 (t, J = 7.2 Hz, 1H, ArH), 6.73 (d, J = 7.2 Hz, 1H, ArH), 6.07 (s, 2H, NH_2), 4.99 (d, J = 16.0Hz, 1H, CH), 4.84 (d, J = 16.0 Hz, 1H, CH), 3.38 (s, 3H, CH_3); ^{13}C NMR (150 MHz, DMSO-d_6) δ: 177.6, 164.7, 150.8, 141.9, 141.0, 140.1, 136.2, 135.4, 133.9, 131.5, 129.2, 128.3, 128.2, 127.3, 127.2, 126.7, 124.0, 122.6, 119.1, 108.6, 104.0, 59.9, 51.2, 49.5, 43.3; IR(KBr) υ: 3426, 3294, 3213, 3068, 2942, 2186, 1709, 1665, 1601, 1562, 1477, 1424, 1361, 1240, 1196, 1116, 1034, 934, 887, 743 cm^{-1}; MS (m/z): HRMS (ESI) Calcd. for $C_{28}H_{20}ClN_4O_3$ ($[M - H]^-$): 495.1241, found: 495.1242.

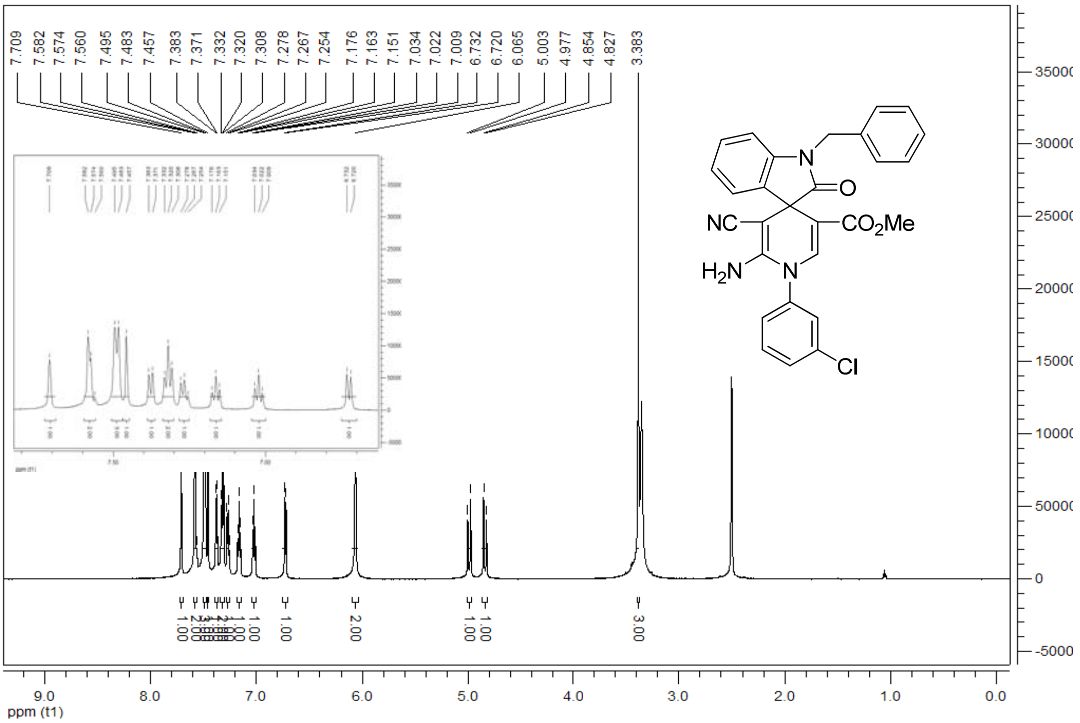

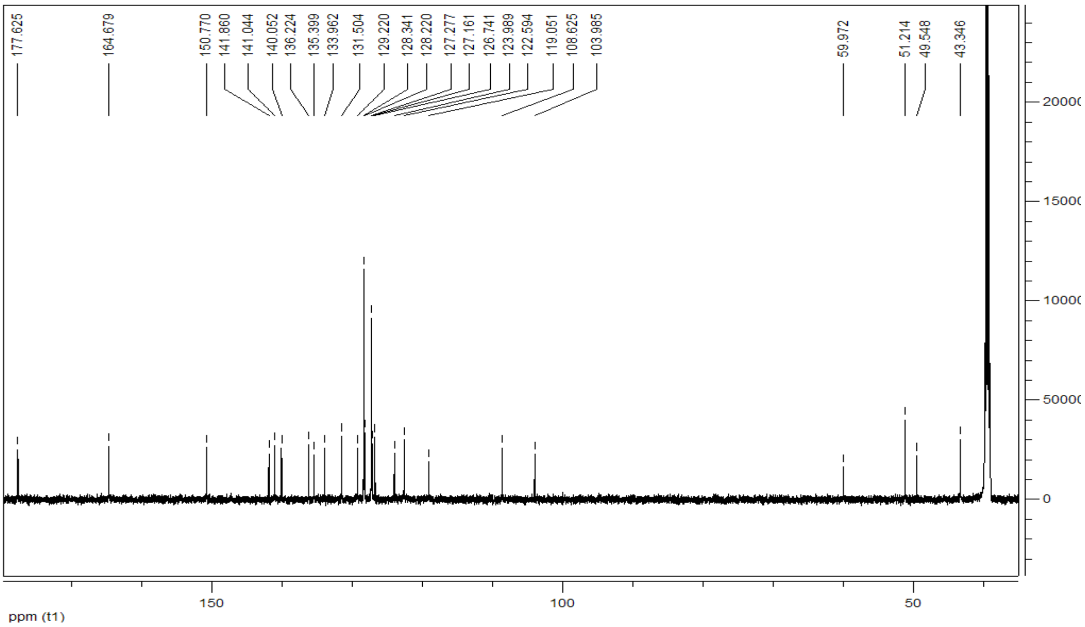

1o: yellow solid, 62%, m.p. 200–202 ℃; ^{1}H NMR (600 MHz, DMSO-d_6) δ: 7.49–7.44 (m, 3H, ArH), 7.41 (s, 1H, ArH), 7.35–7.25 (m, 7H, ArH), 7.16 (t, J = 7.8 Hz, 1H, ArH), 7.03 (t, J = 7.2 Hz, 1H, ArH), 6.73 (d, J = 7.8 Hz, 1H, ArH), 5.87 (s, 2H, NH_2), 4.99 (d, J = 16.2 Hz, 1H, CH), 4.84 (d, J = 16.2 Hz, 1H, CH), 3.37 (s, 3H, CH_3), 2.40 (s, 3H, CH_3); ^{13}C NMR (150 MHz, DMSO-d_6) δ: 177.7, 164.7, 150.9, 141.9, 141.2, 140.0, 138.6, 136.2, 135.5, 129.9, 129.8, 128.3, 128.2, 127.3, 127.2, 124.6, 123.8, 122.6, 119.1, 108.6, 103.6, 59.7, 51.2, 49.6, 43.3, 20.8; IR(KBr) υ: 3454, 3294, 3208, 3029, 2947, 2184, 1710, 1661, 1605, 1558, 1480, 1423, 1347, 1248, 1167, 1116, 1038, 934, 878, 799, 751, 703 cm^{-1}; MS (m/z): HRMS (ESI) Calcd. for $C_{29}H_{23}N_4O_3$ ([M - H]$^-$): 475.1781, found: 475.1781.

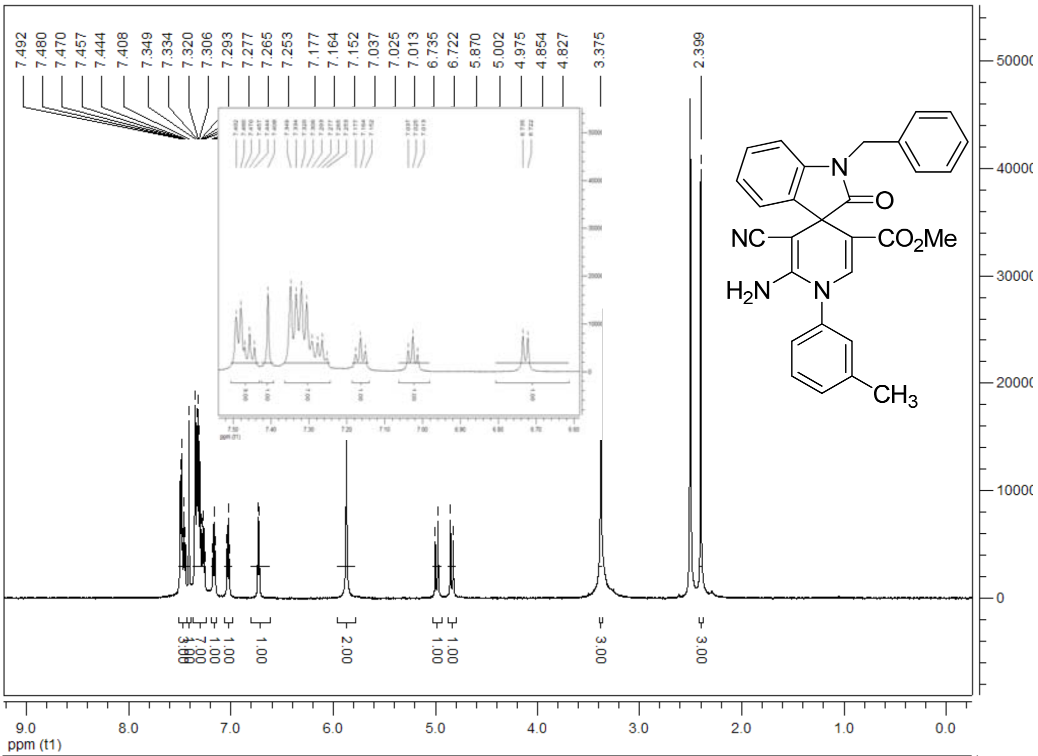

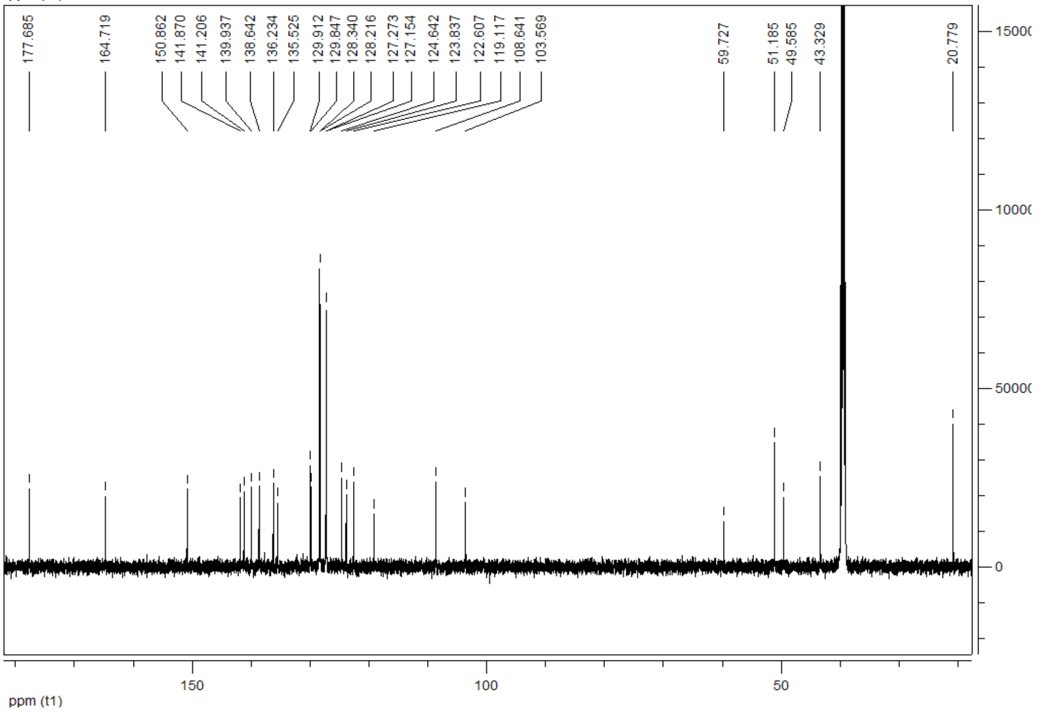

1p: white solid, 50%, m.p. 226–228 ℃; ^{1}H NMR (600 MHz, DMSO-d_6) δ: 7.70 (s, 1H, ArH), 7.45 (t, J = 7.8 Hz, 4H, ArH), 7.36–7.30 (m, 5H, ArH), 7.26 (t, J = 6.6 Hz, 1H, ArH), 7.13 (t, J = 7.2 Hz, 1H, ArH), 7.03 (d, J = 7.2 Hz, 1H, ArH), 6.97 (t, J = 7.2 Hz, 1H, ArH), 6.71 (d, J = 7.8 Hz, 1H, ArH), 6.41 (s, 2H, NH_2), 5.08 (d, J = 16.8 Hz, 1H, CH), 5.02 (d, J = 16.8 Hz, 1H, CH), 4.94 (d, J = 16.0 Hz, 1H, CH), 4.83 (d, J = 16.0 Hz, 1H, CH), 3.86 (s, 3H, CH_3); ^{13}C NMR (150 MHz, DMSO-d_6) δ: 177.9, 164.9, 160.9, 151.6, 142.7, 142.0, 137.1, 136.3, 135.8, 128.7, 128.3, 128.1, 127.5, 127.3, 127.1, 126.4, 123.3, 122.5, 119.5, 108.6, 103.5, 59.8, 51.8, 51.1, 49.6, 43.2; IR(KBr) υ: 3456, 3233, 2949, 2182, 1695, 1669, 1610, 1560, 1468, 1431, 1376, 1349, 1314, 1242, 1202, 1171, 1132, 1081, 1063, 994, 952, 746, 716 cm^{-1}; MS (m/z): HRMS (ESI) Calcd. for $C_{29}H_{23}N_4O_3$ ($[M - H]^-$): 475.1760, found: 475.1761.

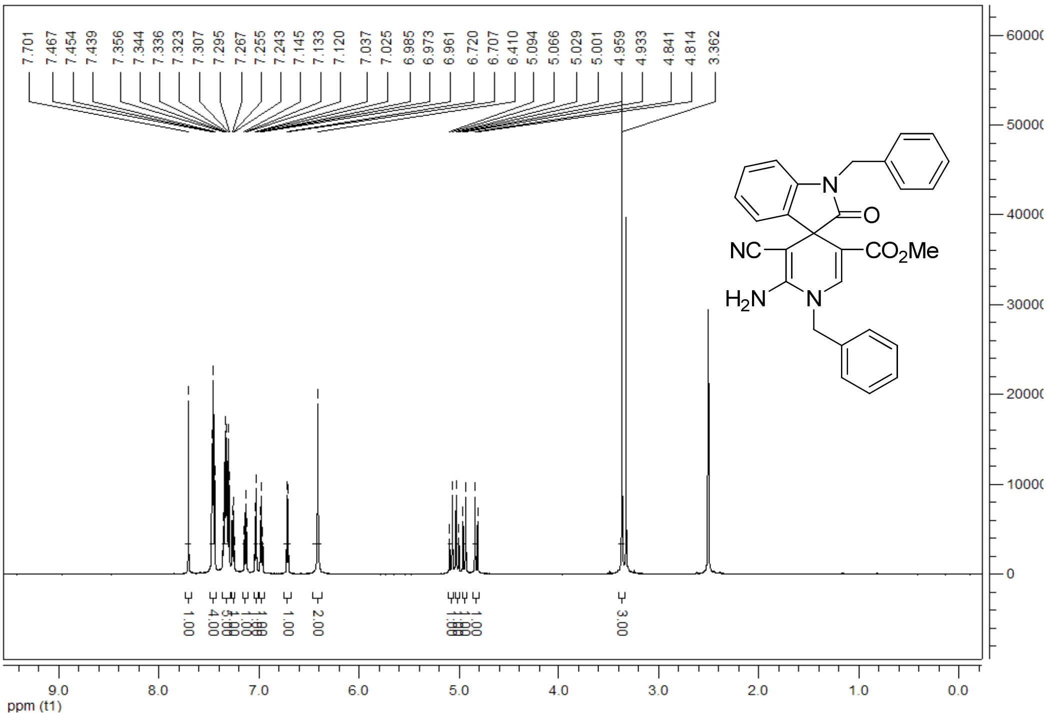

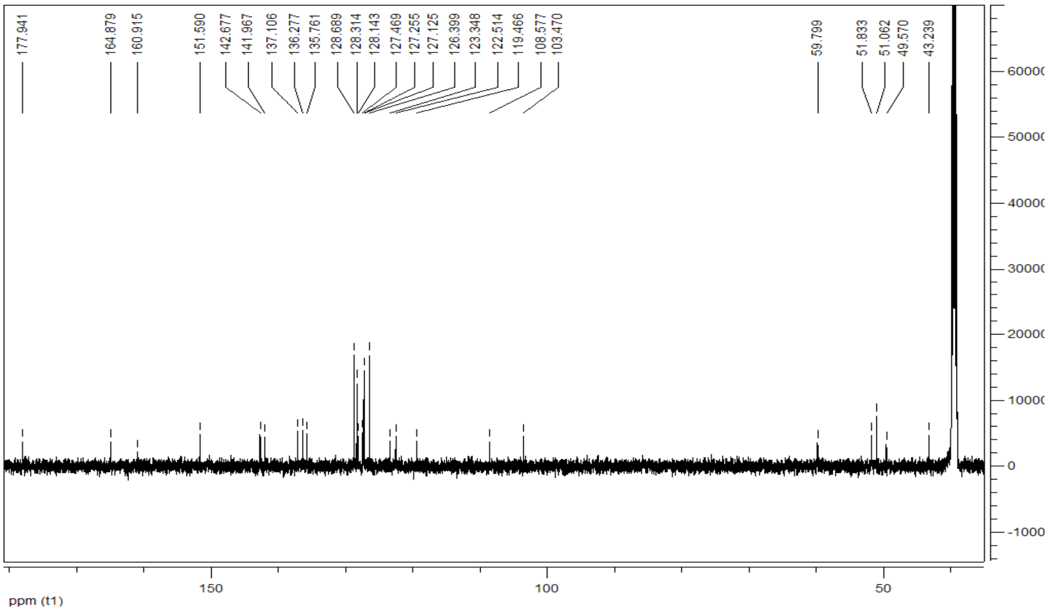

2. General procedure for the synthesis of spiro[indoline-3,4'-pyridines] 2a–2h and spiro[indoline-3,4'-pyridinones] 3a–3n: The similar procedure as above was used. A solution of arylamine (2.0 mmol), methyl propiolate (2.0 mmol) in 5.0 mL ethanol was stirred at room temperature overnight. Then isatin (2.0 mmol), ethyl cyanoacetate (2.0 mmol) and triethylamine (0.4 mmol) were added. The mixture was refluxed for about 24 hours. Then the solution was concentrated to half the volume, which was subjected to column chromatograpy with light petroleum and ethyl acetate (V/V = 1:1) as elute to give the pure product for analysis.

2a: white solid, 49%, m.p. >250 ℃; ^{1}H NMR (600 MHz, DMSO-d_6) δ: 10.04 (s, 1H, NH), 7.65 (d, J = 8.4 Hz, 2H, ArH), 7.56 (d, J = 9.0 Hz, 2H, ArH), 7.40 (br, 2H, NH_2), 7.31 (s, 1H, ArH), 7.12 (d, J = 7.2 Hz, 1H, ArH), 7.06 (t, J = 7.2 Hz, 1H, ArH), 6.82 (t, J = 7.2 Hz, 1H, ArH), 6.66 (d, J = 7.8 Hz, 1H, ArH), 3.71–3.64 (m, 2H, CH_2), 3.38 (s, 3H, CH_3), 0.79 (t, J = 7.2 Hz, 3H, CH_3); ^{13}C NMR (150 MHz, DMSO-d_6) δ: 181.2, 168.6, 164.3, 152.0, 143.1, 139.5, 137.8, 137.7, 133.8, 130.2, 130.0, 127.2, 123.2, 120.7, 107.9, 106.6, 78.3, 58.5, 50.8, 49.7, 13.0; IR(KBr) υ: 3033, 2982, 2953, 2899, 2848, 2347, 2026, 1708, 1673, 1616, 1496, 1434, 1369, 1299, 1252, 1217, 1120, 1048, 1025, 961, 929, 847, 745 cm^{-1}; MS (m/z): HRMS (ESI) Calcd. for $C_{23}H_{20}ClN_3NaO_5$ ([M + Na]$^+$): 476.0984, found: 476.0981.

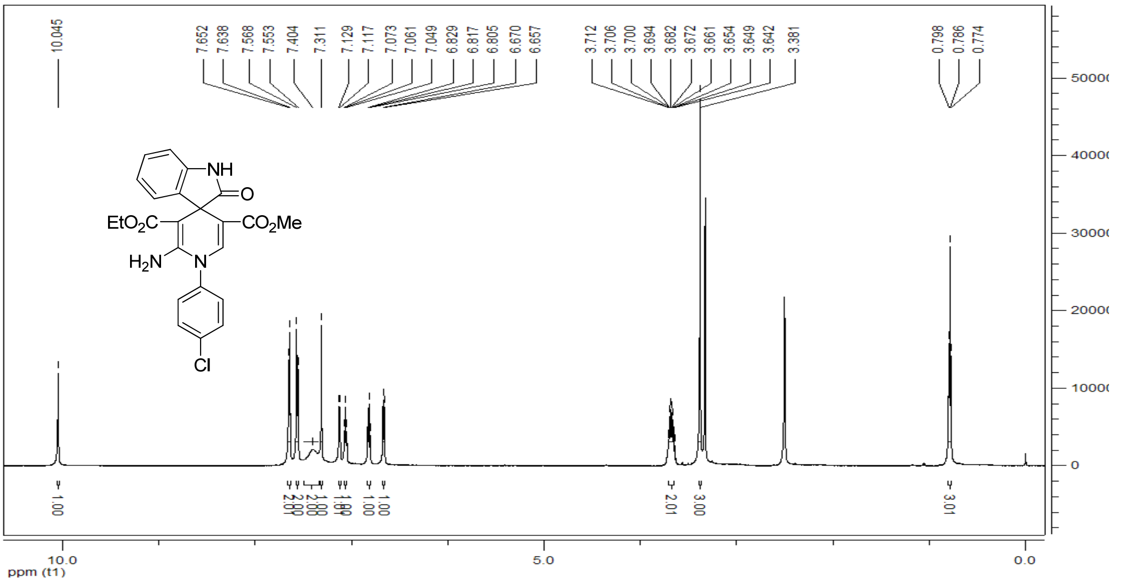

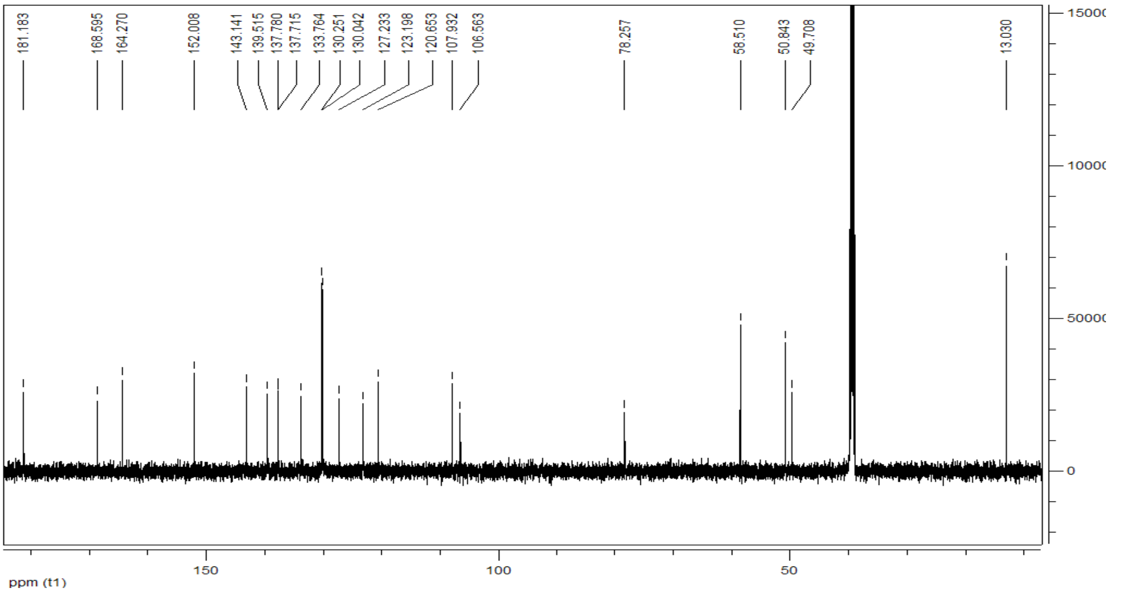

2b: 52%, m.p. 206–208 ℃; ^{1}H NMR (600 MHz, DMSO-*d*$_6$) δ: 7.65 (d, *J* = 9.0 Hz, 2H, ArH), 7.60–7.58 (m, 4H, ArH), 7.46 (br, 2H, NH_2), 7.35 (t, *J* = 9.0 Hz, 3H, ArH), 7.27 (t, *J* = 7.2 Hz, 1H, ArH), 7.22 (d, *J* = 6.6 Hz, 1H, ArH), 7.11 (t, *J* = 7.8 Hz, 1H, ArH), 6.90 (t, *J* = 7.2 Hz, 1H, ArH), 6.79 (d, *J* = 7.8 Hz, 1H, ArH), 4.91 (d, *J* = 15.6 Hz, 1H, CH), 4.75 (d, *J* = 15.6 Hz, 1H, CH), 3.76–3.71 (m, 1H, CH_2), 3.40–3.36 (m, 1H, CH_2), 3.28 (s, 3H, CH_3), 0.44 (t, *J* = 7.2 Hz, 3H, CH_3) ;^{13}C NMR (150 MHz, DMSO-*d*$_6$) δ: 179.7, 168.4, 164.5, 152.2, 144.0, 139.9, 137.7, 137.2, 137.1, 133.8, 130.3, 130.1, 128.3, 128.2, 127.3, 127.1, 123.1, 121.5, 107.3, 106.2, 78.2, 58.1, 50.9, 49.3, 44.2, 13.5; IR(KBr) υ: 3354, 3062, 2982, 1714, 1670, 1604, 1485, 1431, 1364, 1308, 1252, 1111, 1024, 930, 841, 754 cm^{-1}; MS (*m/z*): HRMS (ESI) Calcd. for $C_{30}H_{26}ClN_3NaO_5$ ([M + Na]$^+$): 566.1448, found: 566.1451.

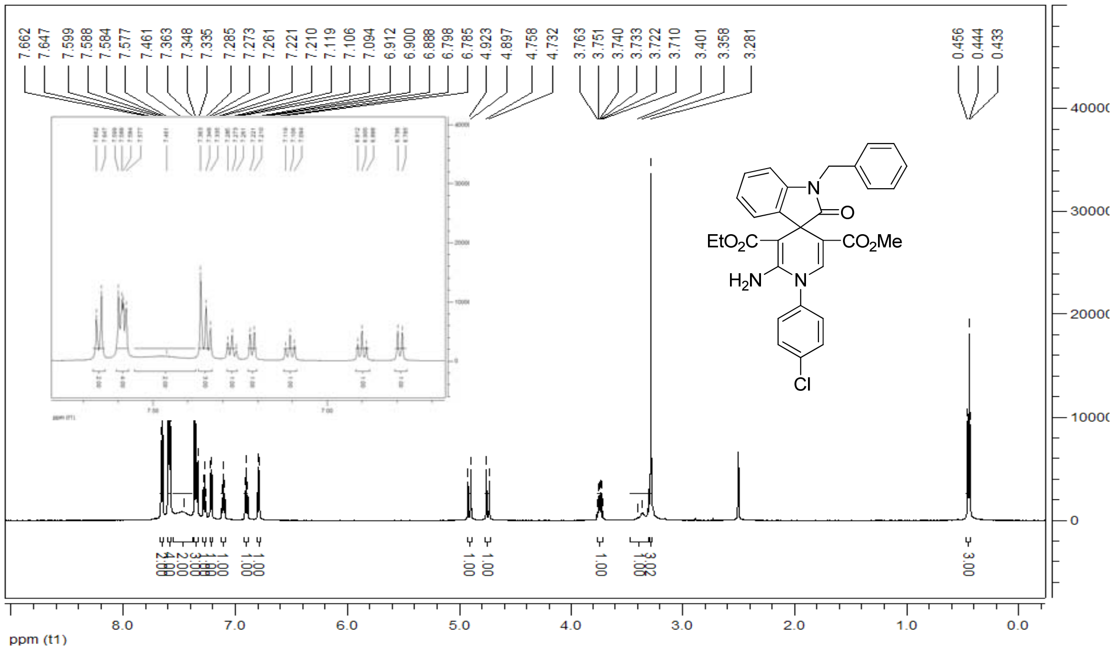

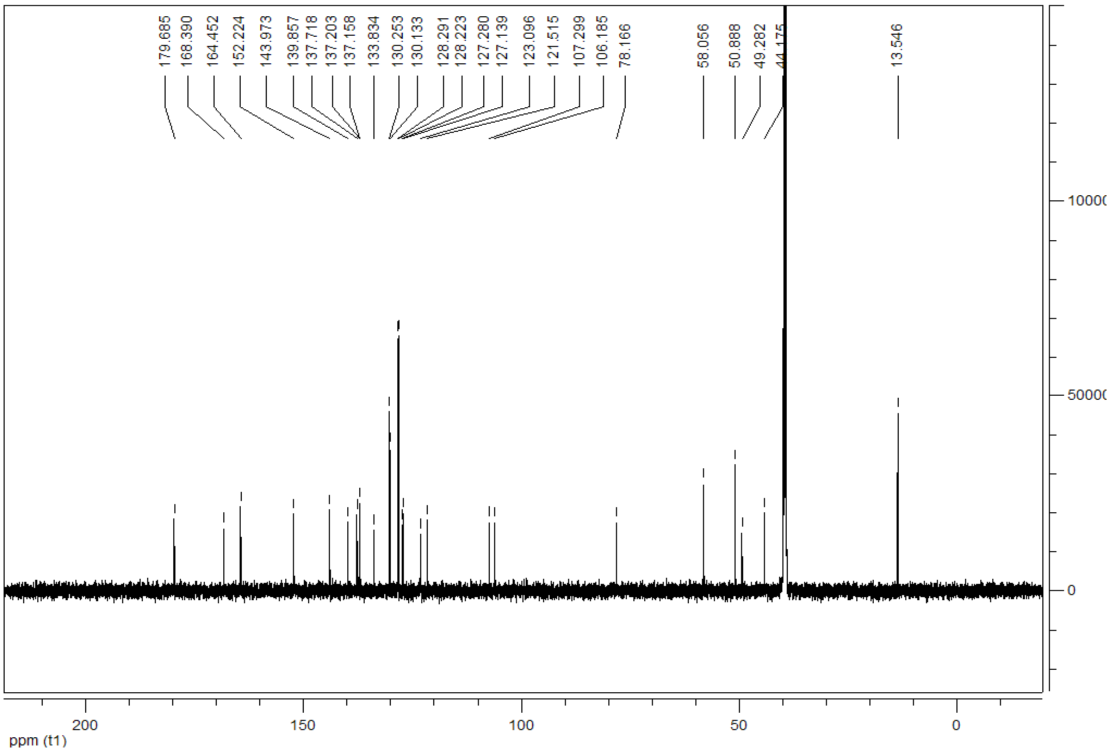

2c: white solid, 47%, m.p. >250 ℃; ^{1}H NMR (600 MHz, DMSO-d_6) δ: 7.65 (d, J = 7.8 Hz, 2H, ArH), 7.58 (d, J = 8.4 Hz, 2H, ArH), 7.43 (br, 2H, NH_2), 7.32 (s, 1H, ArH), 7.01 (s, 1H, ArH), 6.95 (d, J = 7.8 Hz, 1H, ArH), 6.72 (d, J = 7.8 Hz, 1H, ArH), 3.70–3.66 (m, 2H, CH_2), 3.62–3.58 (m, 1H, CH_2), 3.46–3.42 (m, 1H, CH_2), 3.35 (s, 3H, CH_3), 2.24 (s, 3H, CH_3), 1.61–1.60 (m, 2H, CH_2), 1.44–1.40 (m, 2H, CH_2), 0.94 (t, J = 7.8 Hz, 3H, CH_3), 0.68 (t, J = 7.2 Hz, 3H, CH_3); ^{13}C NMR (150 MHz, DMSO-d_6) δ: 178.9, 168.5, 164.3, 152.2, 141.8, 139.6, 137.8, 137.1, 133.8, 130.2, 130.1, 129.8, 127.6, 123.9, 106.5, 106.4, 78.1, 58.1, 50.9, 49.2, 29.1, 20.6, 19.9, 13.8, 13.6; IR(KBr) υ: 3170, 2958, 2669, 2026, 1709, 1671, 1602, 1491, 1428, 1369, 1314, 1252, 1213, 1114, 1033, 909, 838, 804, 750 cm^{-1}; MS (m/z): HRMS (ESI) Calcd. for $C_{28}H_{30}ClN_3NaO_5$ ([M + Na]$^+$): 546.1766, found: 546.1767.

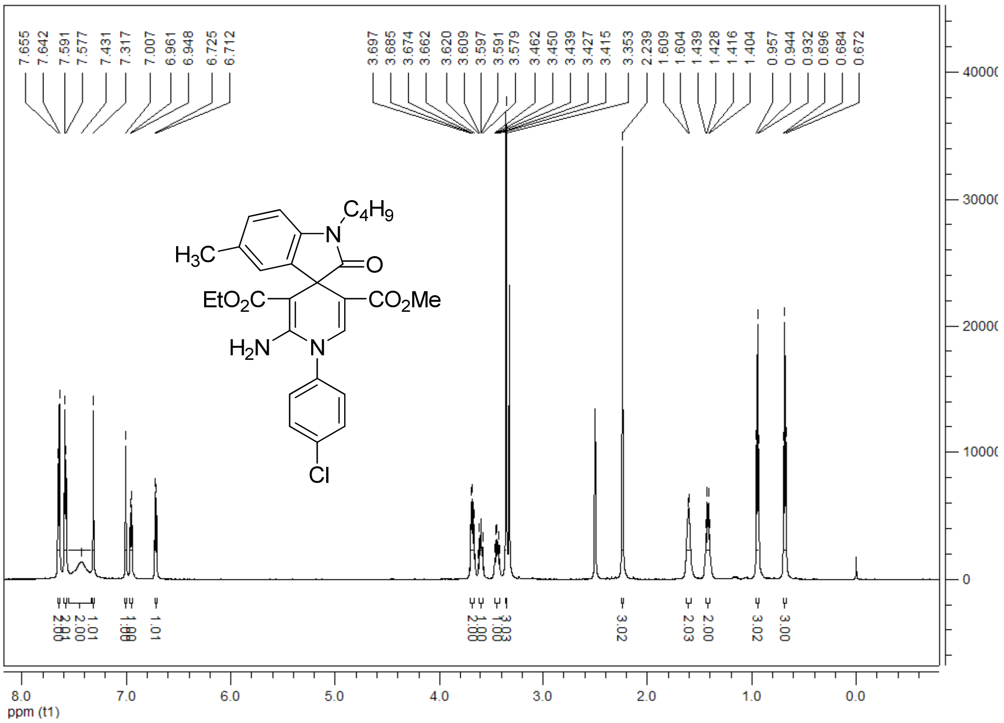

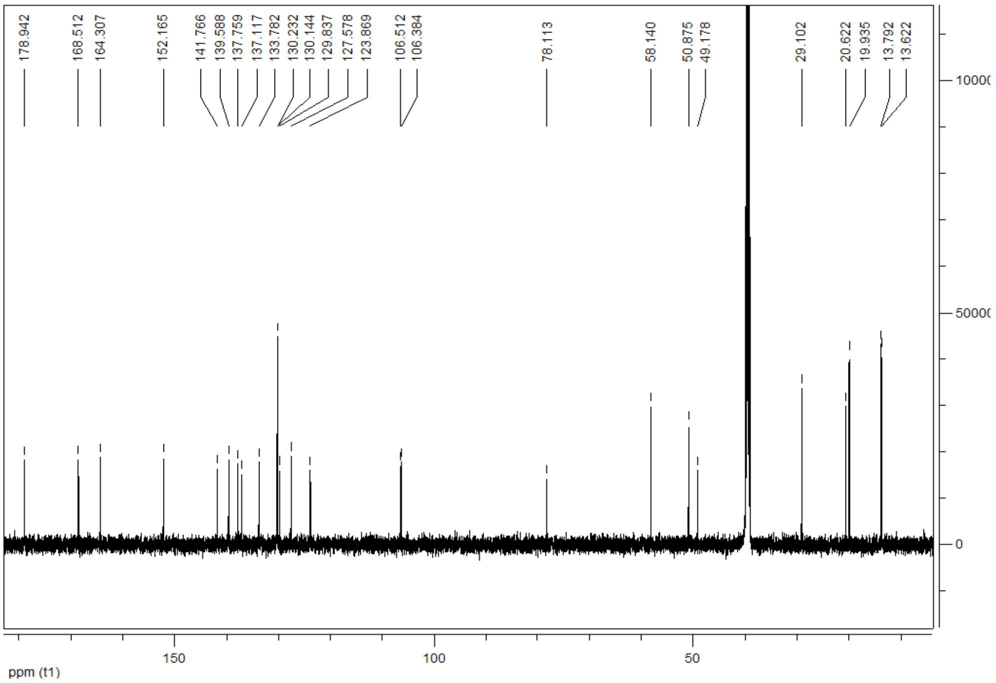

2d: white solid, 35%, m.p. 196–198 ℃; ^{1}H NMR (600 MHz, DMSO-d_6) δ: 7.65 (d, J = 8.4 Hz, 2H, ArH), 7.59 (d, J = 7.8 Hz, 2H, ArH), 7.57 (d, J = 7.2 Hz, 2H, ArH), 7.45 (br, 2H, NH_2), 7.34 (t, J = 7.8 Hz, 3H, ArH),7.27 (t, J = 7.2 Hz, 1H, ArH), 7.03 (s, 1H, ArH), 6.90 (d, J = 7.2 Hz, 1H, ArH), 6.65 (d, J = 7.8 Hz, 1H, ArH), 4.89 (d, J = 15.5 Hz , 1H, CH), 4.70 (d, J = 15.5 Hz , 1H, CH), 3.73–3.70 (m, 1H, CH_2), 3.37–3.34 (m, 1H, CH_2), 3.28 (s, 3H, CH_3), 2.20 (s, 3H, CH_3), 0.49 (t, J = 7.8 Hz, 3H, CH_3); ^{13}C NMR (150 MHz, DMSO-d_6) δ: 179.6, 168.4, 164.4, 152.2, 141.7, 139.8, 137.7, 137.2, 133.8, 130.3, 130.2, 130.1, 128.2, 128.1, 127.4, 127.1, 123.8, 107.1, 106.3, 78.3, 58.1, 50.9, 49.3, 44.2, 20.6, 13.6; IR(KBr) υ: 3463, 3170, 2981, 2949, 1711, 1673, 1601, 1492, 1427, 1371, 1312, 1252, 1187, 1109, 1032, 937, 911, 882, 846, 807, 727 cm^{-1}; MS (m/z): HRMS (ESI) Calcd. for $C_{31}H_{27}ClN_3O_5$ ([M - H]$^-$): 556.1593, found: 556.1592.

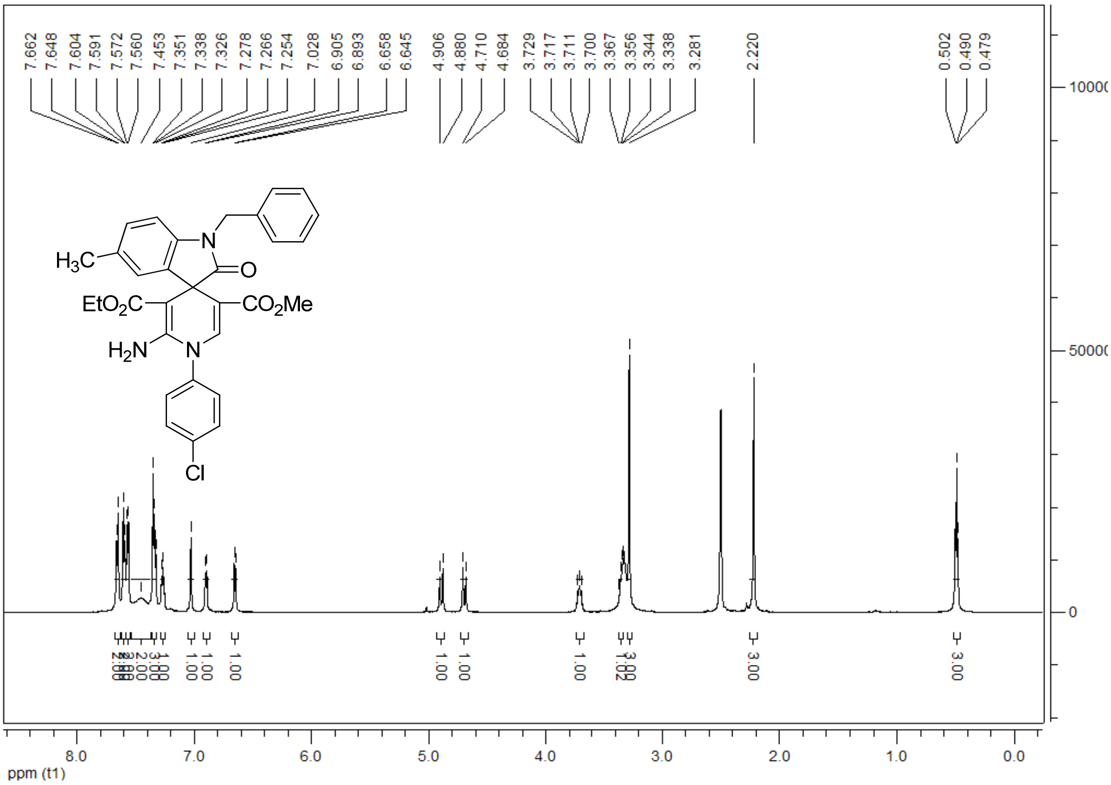

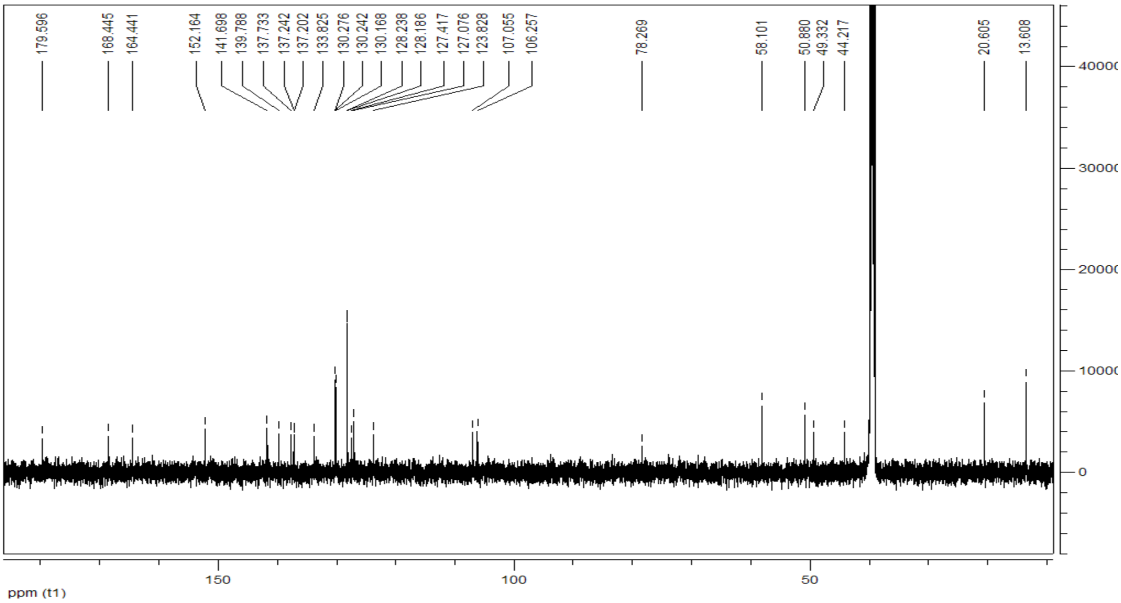

2e: white solid, 36%, m.p. 176–178 ℃; ^{1}H NMR (600 MHz, DMSO-d_6) δ: 7.77 (s, 1H, ArH), 7.62 (d, J = 4.2 Hz, 2H, ArH), 7.58 (d, J = 7.8 Hz, 2H, ArH), 7.54 (d, J = 3.6 Hz, 1H, ArH), 7.49 (br, 2H, NH_2), 7.39 (s, 1H, ArH), 7.34 (t, J = 7.4 Hz, 2H, ArH), 7.27 (t, J = 7.2 Hz, 1H, ArH), 7.09 (s, 1H, ArH), 6.90 (d, J = 7.8 Hz, 1H, ArH), 6.66 (d, J = 7.8 Hz, 1H, ArH), 4.92 (d, J = 15.5 Hz, 1H, CH), 4.71 (d, J = 15.5 Hz, 1H, CH), 3.75–3.72 (m, 1H, CH_2), 3.37–3.34 (m, 1H, CH_2), 3.29 (s, 3H, CH_3), 2.23 (s, 3H, CH_3), 0.50 (t, J = 7.2 Hz, 3H, CH_3); ^{13}C NMR (150 MHz, DMSO-d_6) δ: 179.6, 168.5, 164.5, 152.1, 141.7, 140.1, 139.7, 137.3, 137.2, 134.2, 131.7, 130.3, 129.4, 128.5, 128.2, 128.1, 127.4, 127.1, 127.0, 124.0, 107.0, 106.3, 78.4, 58.1, 50.9, 49.3, 44.2, 20.6, 13.6; IR(KBr) υ: 3186, 2967, 2026, 1715, 1673, 1595, 1489, 1428, 1373, 1339, 1306, 1248, 1102, 1026, 908, 876, 792, 699 cm^{-1}; MS (m/z): HRMS (ESI) Calcd. for $C_{31}H_{28}ClN_3NaO_5$ ($[M + Na]^+$): 580.1610, found: 580.1606.

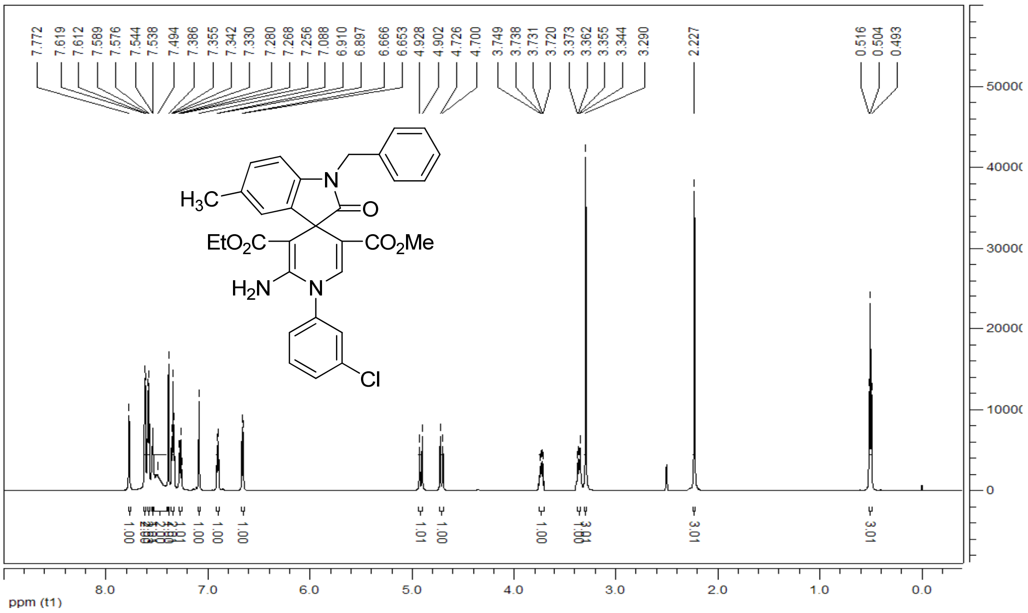

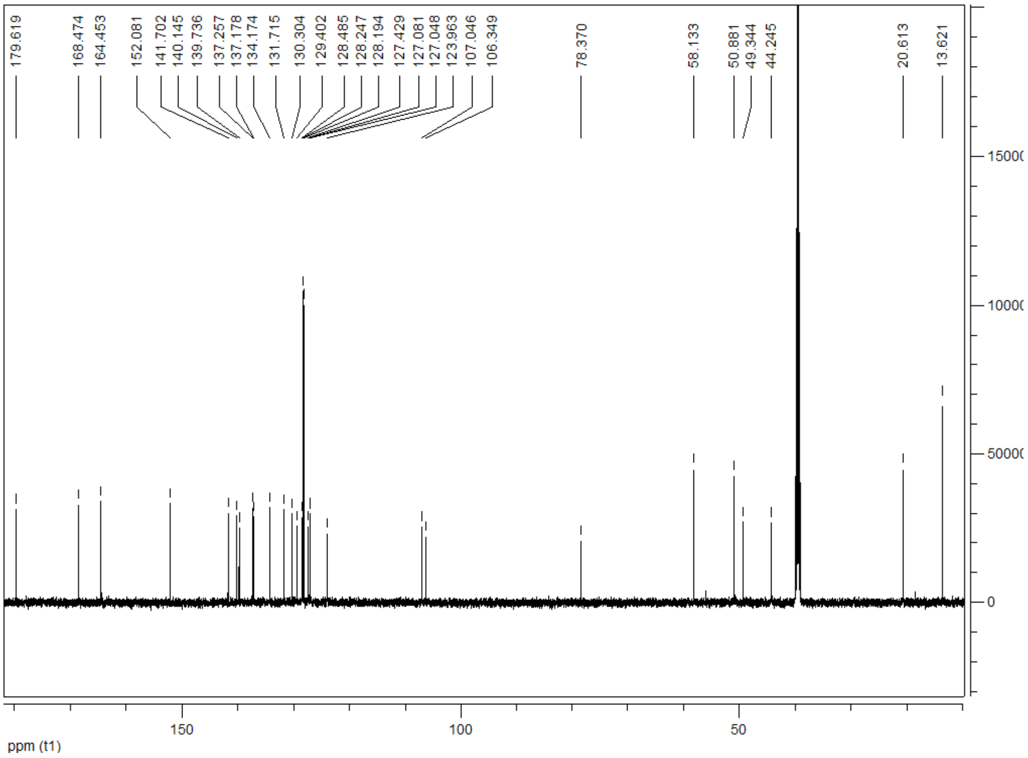

2f: white solid, 52%, m.p. >250 ℃; ^{1}H NMR (600 MHz, DMSO-d_6) δ: 10.06 (s, 1H, NH), 7.78 (d, J = 7.8 Hz, 2H, ArH), 7.49 (d, J = 7.2 Hz, 2H, ArH), 7.41 (br, 2H, NH_2), 7.31 (s, 1H, ArH), 7.12 (d, J = 7.2 Hz, 1H, ArH), 7.06 (t, J = 7.8 Hz, 1H, ArH), 6.82 (t, J = 7.2 Hz, 1H, ArH), 6.66 (d, J = 7.2 Hz, 1H, ArH), 3.71~3.64 (m, 2H, CH_2), 3.38 (s, 3H, CH_3), 0.78 (t, J = 7.2 Hz, 3H, CH_3); ^{13}C NMR (150 MHz, DMSO-d_6) δ: 181.1, 168.6, 164.3, 152.0, 143.2, 139.4, 138.2, 137.8, 133.2, 130.3, 127.2, 123.2, 122.3, 120.6, 107.9, 106.7, 78.4, 58.5, 50.8, 49.7, 13.1; IR(KBr) υ: 3352, 3183, 3078, 3032, 2980, 2896, 2845, 2740, 1711, 1671, 1613, 1489, 1433, 1367, 1299, 1253, 1117, 1041, 921, 845, 752, 653 cm^{-1}; MS (m/z): HRMS (ESI) Calcd. for $C_{24}H_{21}BrN_2O_5$ ($[M-H]^-$): 496.0514, found: 496.0462.

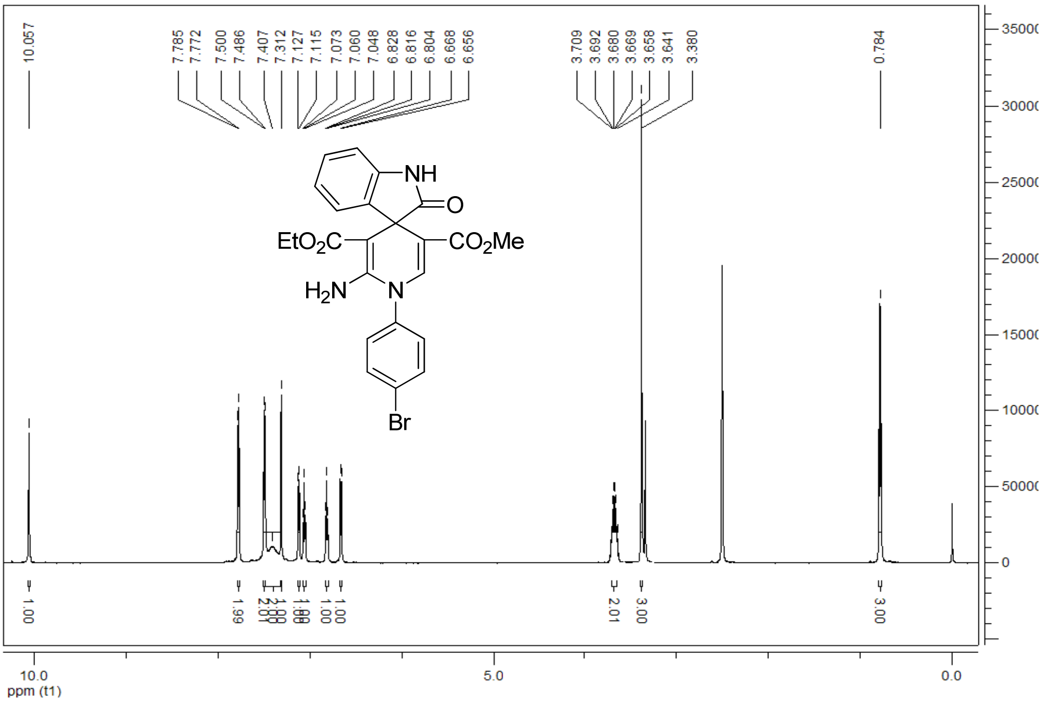

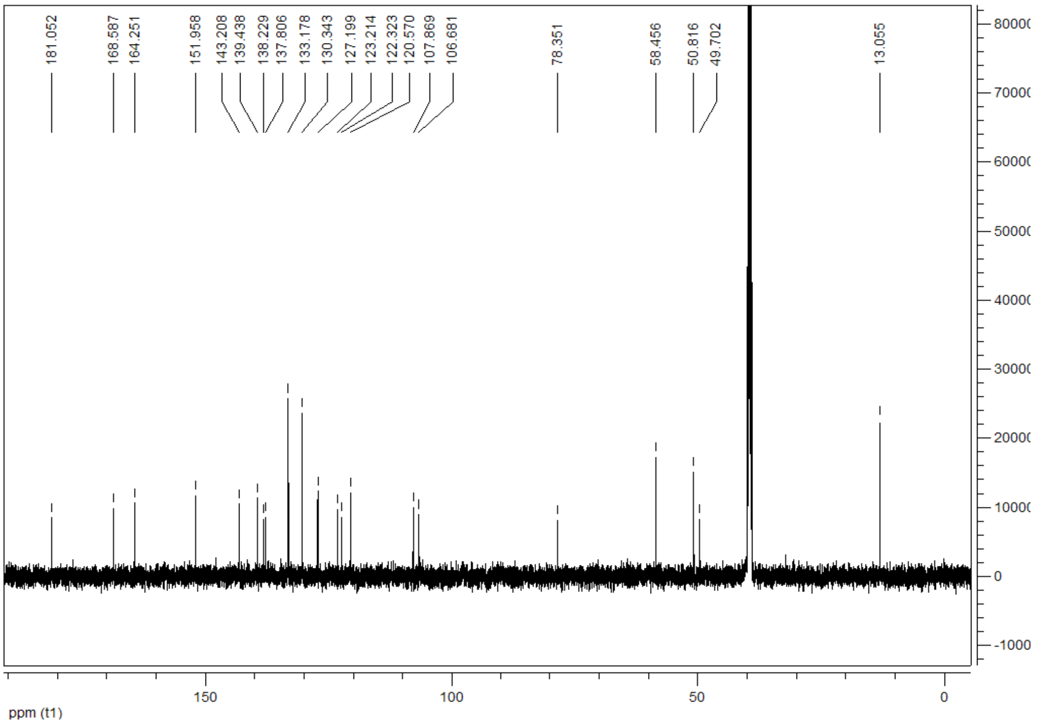

2g: white solid, 36%, m.p. >250 ℃; ^{1}H NMR (600 MHz, DMSO-d_6) δ: 7.78 (d, J = 7.8 Hz, 2H, ArH), 7.51 (d, J = 7.8 Hz, 2H, ArH), 7.45 (br, 2H, NH_2), 7.33 (s, 1H, ArH), 7.19 (d, J = 7.2 Hz, 1H, ArH), 7.16 (t, J = 7.2 Hz, 1H, ArH), 6.89 (t, J = 7.8 Hz, 1H, ArH), 6.84 (d, J = 7.8 Hz, 1H, ArH), 3.74–3.67 (m, 2H, CH_2), 3.60–3.54 (m, 1H, CH_2), 3.50–3.46 (m, 1H, CH_2), 3.50 (s, 3H, CH_3), 1.62–1.60 (m, 2H, CH_2), 1.45–1.41 (m, 2H, CH_2), 0.95 (t, J = 7.2 Hz, 3H, CH_3), 0.66 (t, J = 7.2 Hz, 3H, CH_3); ^{13}C NMR (150 MHz, DMSO-d_6) δ: 179.0, 168.4, 164.3, 152.2, 144.0, 139.6, 138.2, 137.1, 133.2, 130.4, 127.4, 123.1, 122.4, 121.1, 106.6, 106.5, 78.1, 58.1, 50.9, 49.1, 29.1, 19.9, 13.8, 13.6; IR(KBr) υ: 3446, 3177, 2959, 2867, 2027, 1713, 1669, 1604, 1481, 1430, 1367, 1315, 1251, 1193, 1110, 1024, 926, 839, 754, 690 cm^{-1}; MS (m/z): HRMS (ESI) Calcd. for $C_{28}H_{29}BrN_2O_5$ ([M - H]$^-$): 552.1140, found: 552.1136.

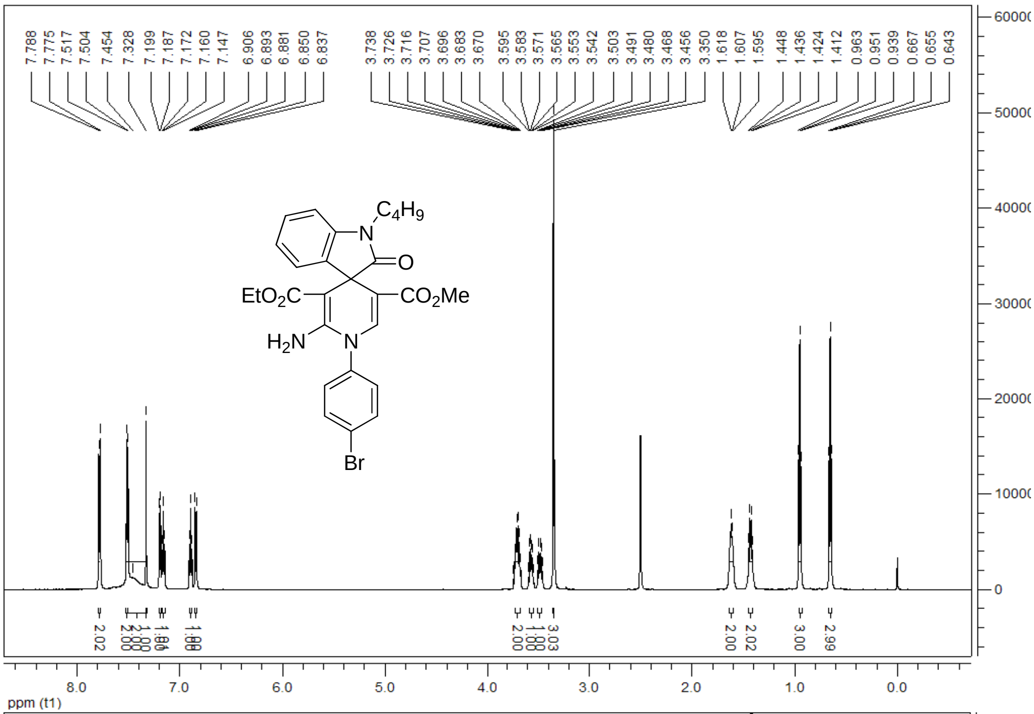

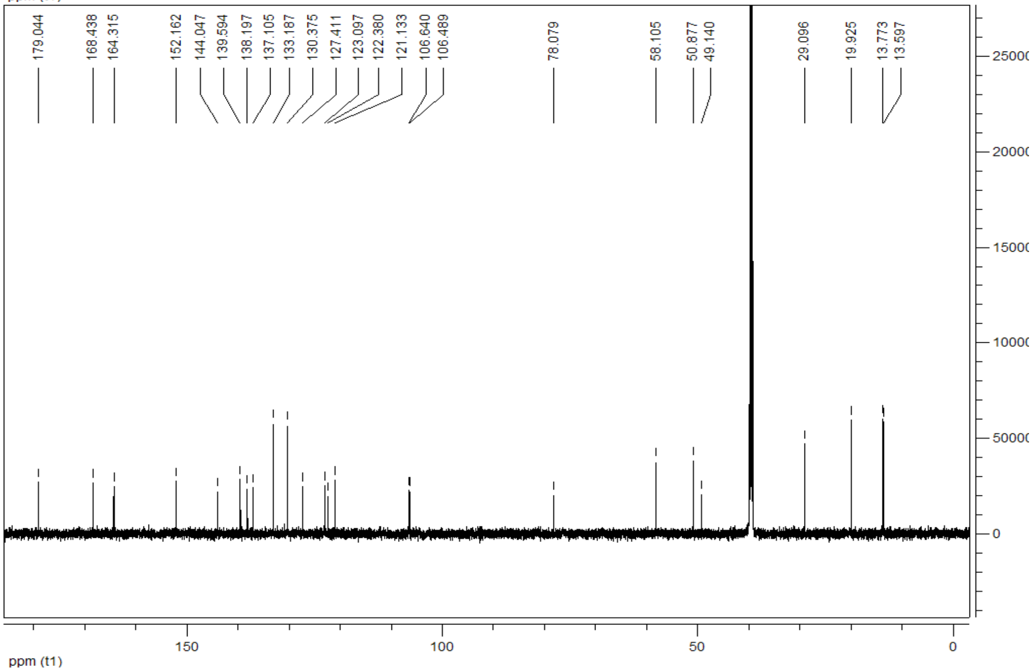

2h: white solid, 56%, m.p. 236–238 ℃; ^{1}H NMR (600 MHz, DMSO-d_6) δ: 7.79 (d, J = 7.8 Hz, 2H, ArH), 7.57 (d, J = 7.2 Hz, 2H, ArH), 7.53 (d, J = 7.8 Hz, 2H, ArH), 7.48 (br, 2H, NH_2), 7.36–7.33 (m, 3H, ArH), 7.27 (d, J = 7.2 Hz, 1H, ArH), 7.03 (s, 1H, ArH), 6.90 (d, J = 7.2 Hz, 1H, ArH), 6.66 (d, J = 7.2 Hz, 1H, ArH), 4.90 (d, J = 15.4 Hz, 1H, CH), 4.71 (d, J = 15.4 Hz, 1H, CH), 3.74–3.71 (m, 1H, CH_2), 3.36–3.34 (s, 4H, CH, CH_3), 2.22 (s, 3H, CH_3), 0.50 (t, J = 7.2 Hz, 3H, CH_3); ^{13}C NMR (150 MHz, DMSO-d_6) δ: 179.6, 168.5, 164.4, 152.1, 141.7, 139.7, 138.2, 137.3, 137.2, 133.2, 130.4,130.3, 128.2, 128.1, 127.4, 127.1, 123.8, 122.4, 107.1, 106.3, 78.3, 58.1, 50.9, 49.3, 44.2, 20.6, 13.6; IR(KBr) υ: 3165, 2982, 2026, 1711, 1671, 1601, 1489, 1427, 1313, 1251, 1187, 1107, 1032, 909, 804, 692 cm^{-1}; MS (m/z): HRMS (ESI) Calcd. for $C_{31}H_{28}BrN_3NaO_5$ ([M + Na]$^+$): 624.1105, found: 624.1111.

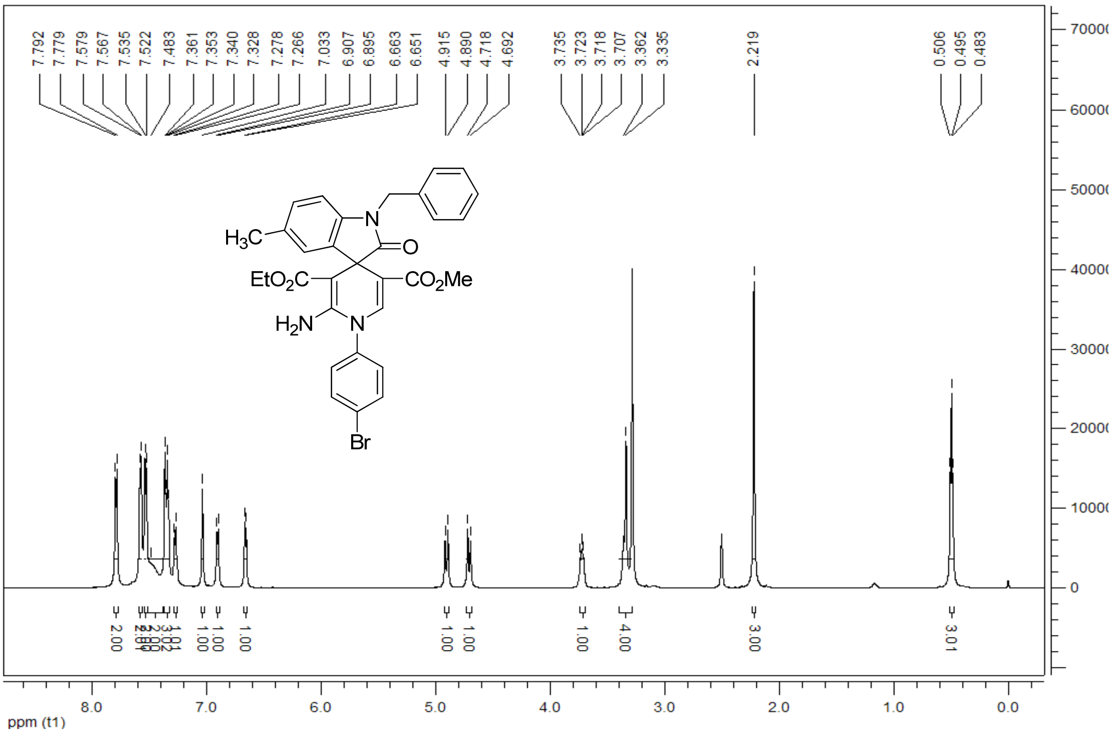

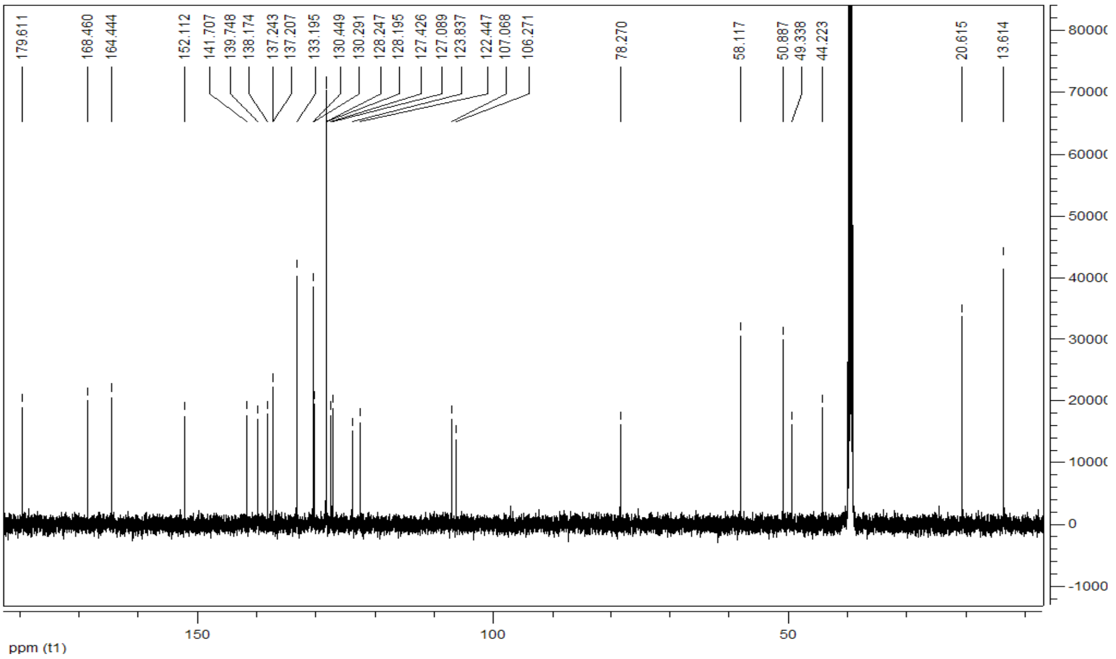

3a: white solid, 39%, m.p. 244–246 ℃; ^{1}H NMR (600 MHz, DMSO-d_6) δ: 11.03 (s, 1H, NH), 7.65 (s, 1H, ArH), 7.45 (d, J = 7.8 Hz, 2H, ArH), 7.31 (d, J = 24.6 Hz, 1H, ArH), 7.18 (d, J = 6.6 Hz, 1H, ArH), 7.08 (d, J = 7.8 Hz, 2H, ArH), 7.04 (d, J = 7.2 Hz, 1H, ArH), 6.97 (d, J = 7.2 Hz, 1H, ArH), 5.39 (s, 1H, CH), 3.82 (s, 3H, CH_3), 3.56 (s, 3H, CH_3); ^{13}C NMR (150 MHz, DMSO-d_6) δ: 175.7, 163.5, 161.1, 159.2, 142.4, 142.2, 131.4, 130.2, 127.9, 127.1, 123.1, 122.5, 114.7, 114.6, 113.2, 110.3, 108.6, 55.5, 51.8, 51.4, 44.0; IR(KBr) υ: 3756, 3311, 2928, 2869, 2374, 1732, 1679, 1635, 1511, 1141, 1329, 1265, 1179, 1126, 1034, 835, 751 cm^{-1}; MS (m/z): HRMS (ESI) Calcd. for $C_{22}H_{17}N_3NaO_5$ ([M + Na]$^+$): 426.1060, found: 426.1061.

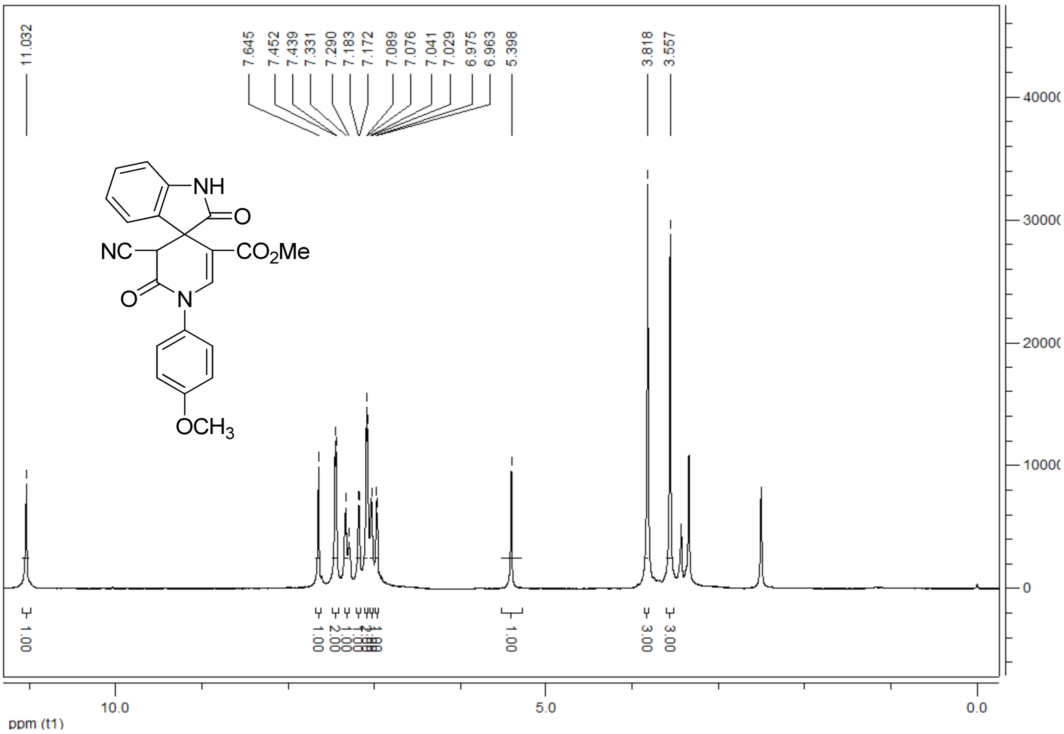

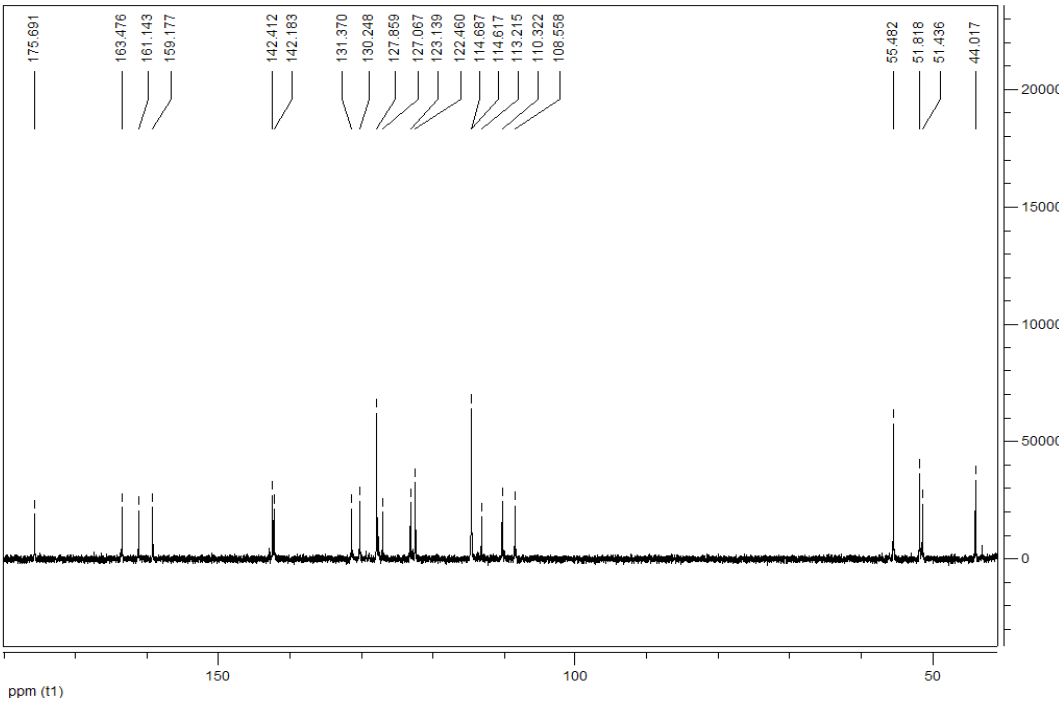

3b: white solid, 53%, m.p. 224–226 ℃; ^{1}H NMR (600 MHz, DMSO-d_6) δ: 7.71 (s, 1H, ArH), 7.51 (d, J = 7.8 Hz, 2H, ArH), 7.47 (d, J = 9.0 Hz, 2H, ArH), 7.35 (t, J = 7.2 Hz, 3H, ArH), 7.29 (t, J = 9.0 Hz, 2H, ArH), 7.09 (d, J = 9.0 Hz, 3H, ArH), 6.97 (d, J = 7.8 Hz, 1H, ArH), 5.57 (s, 1H, CH), 5.06 (d, J = 16.0 Hz, 1H, CH), 5.00 (d, J = 16.0 Hz, 1H, CH), 3.82 (s, 3H, CH_3), 3.53 (s, 3H, CH_3); ^{13}C NMR (150 MHz, DMSO-d_6) δ: 174.4, 163.5, 161.0, 159.2, 142.8, 142.7, 135.7, 131.3, 130.3, 128.5, 127.9, 127.6, 127.4, 127.3, 127.1, 126.3, 123.3, 123.1, 114.7, 114.6, 113.2, 110.0, 108.1, 55.5, 51.9, 51.0, 43.9, 43.7; IR(KBr) υ: 3405, 3036, 2950, 2861, 1706, 1642, 1606, 1508, 1452, 1337, 1253, 1180, 1125, 1030, 993, 912, 837, 755 cm^{-1}; MS (m/z): HRMS (ESI) Calcd. for $C_{29}H_{22}N_3O_5$ ($[M - H]^-$): 492.1566, found: 492.1567.

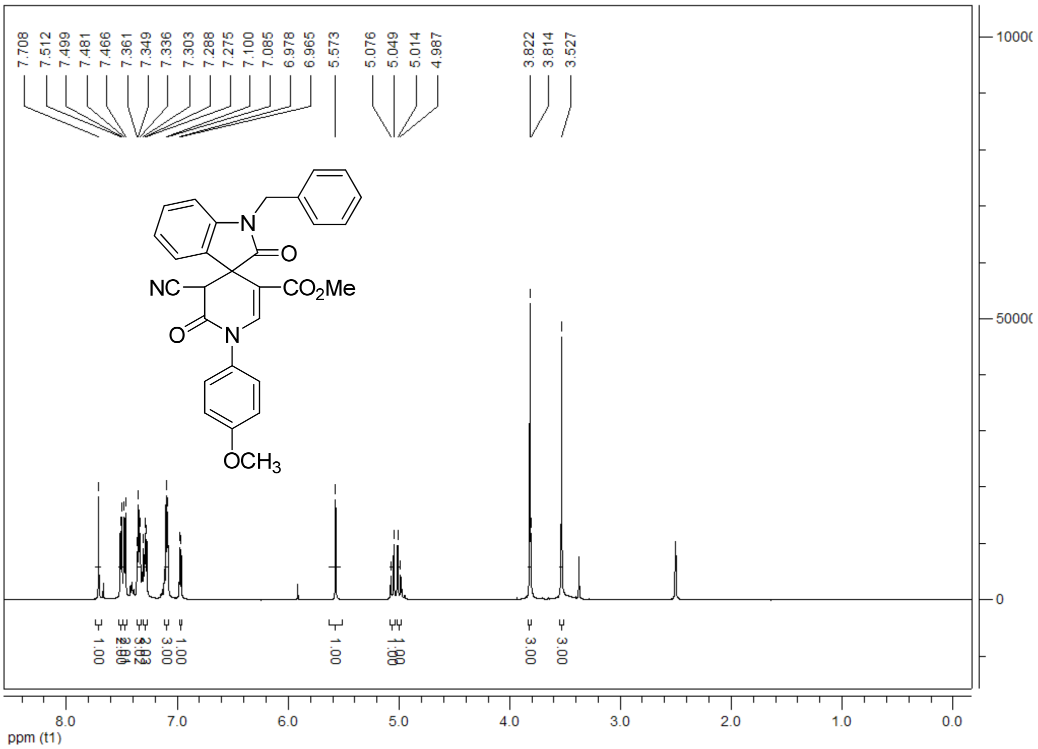

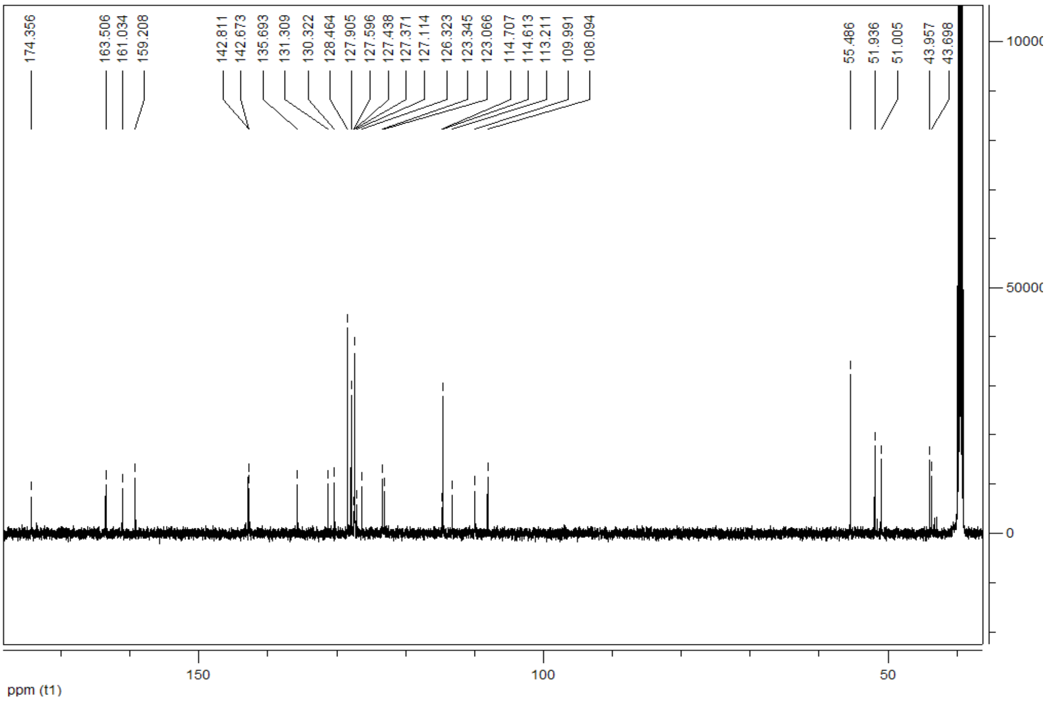

3c: white solid, 54%, m.p. 216–218 ℃; ^{1}H NMR (600 MHz, DMSO-d_6) δ: 7.66 (s, 1H, ArH), 7.46–7.41 (m, 3H, ArH), 7.24–7.20 (m, 2H, ArH), 7.11–7.08 (m, 3H, ArH), 5.46 (s, 1H, CH), 3.82 (s, 3H, CH_3), 3.74–3.71 (m, 2H, CH_2), 3.53 (s, 3H, CH_3), 1.65–1.62 (m, 2H, CH_2), 1.44–1.41 (m, 2H, CH_2), 0.93 (t, J = 7.2 Hz, 3H, CH_3); ^{13}C NMR (150 MHz, DMSO-d_6) δ: 174.0, 163.4, 161.1, 159.2, 143.1, 142.6, 131.4, 130.4, 127.9, 127.6, 126.4, 123.0, 114.7, 114.6, 113.0, 109.5, 108.3, 55.5, 51.9, 50.9, 44.0, 29.0, 19.5, 13.7; IR(KBr) υ: 3464, 2958, 2865, 2373, 1704, 1640, 1607, 1511, 1463, 1339, 1257, 1183, 1130, 1032, 987, 918, 887, 833, 757 cm^{-1}; MS (m/z): HRMS (ESI) Calcd. for $C_{26}H_{25}N_3NaO_5$ ($[M + Na]^+$): 482.1686, found: 482.1685.

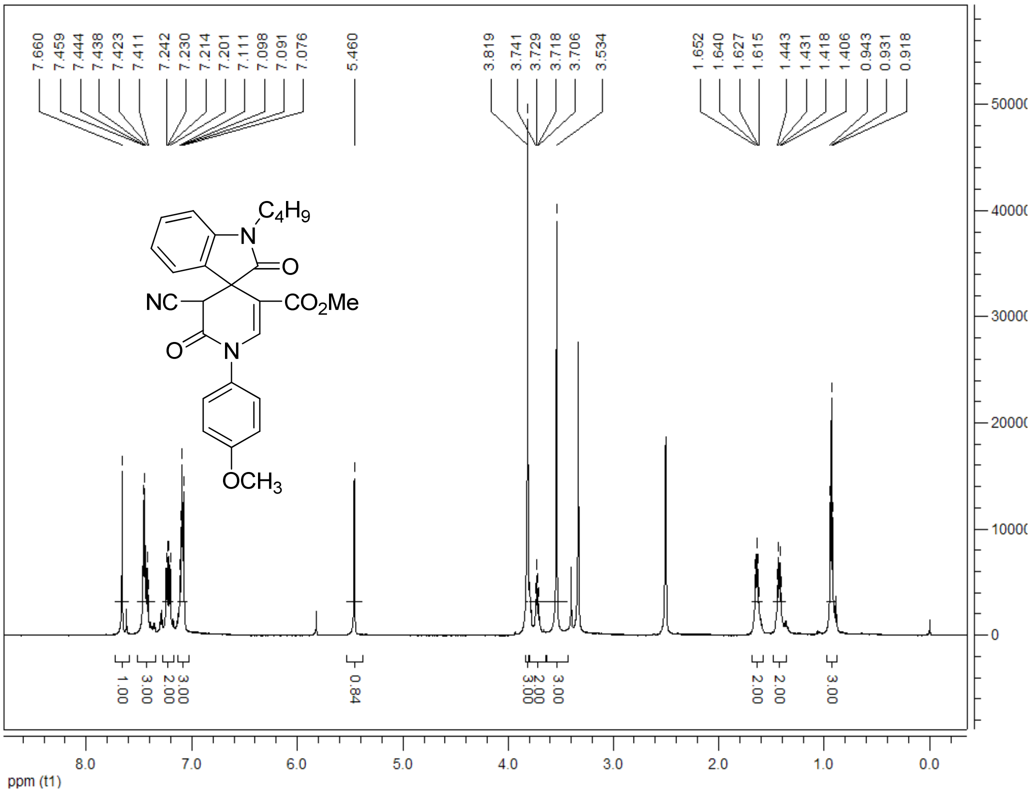

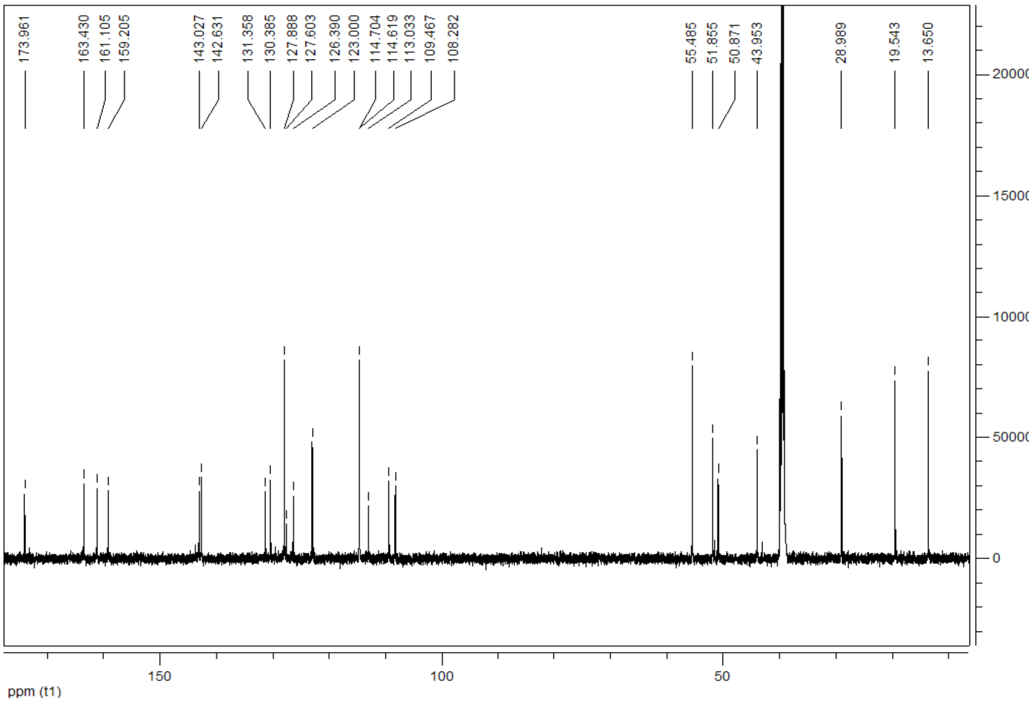

3d: white solid, 51%, m.p. 248–250℃; ^{1}H NMR (600 MHz, DMSO-d_6) δ: 7.70 (s, 1H, ArH), 7.48 (t, J = 6.0 Hz, 4H, ArH), 7.34 (t, J = 6.6 Hz, 2H, ArH), 7.29 (d, J = 6.6 Hz, 1H, ArH), 7.14 (d, J = 7.2 Hz, 1H, ArH), 7.10 (d, J = 7.2 Hz, 1H, ArH), 7.03 (s, 1H, ArH), 6.84 (d, J = 7.8 Hz, 1H, ArH), 5.55 (s, 1H, CH), 5.03 (d, J = 16.0 Hz, 1H, CH), 4.97 (d, J = 16.0 Hz, 1H, CH), 3.82 (s, 3H, CH_3), 3.52 (s, 3H, CH_3), 2.27 (s, 3H, CH_3); ^{13}C NMR (150 MHz, DMSO-d_6) δ: 174.3, 163.5, 161.0, 159.2, 142.7, 140.4, 135.8, 132.4, 131.4, 130.6, 128.4,127.9, 127.4, 127.3, 126.4, 123.4, 114.7, 114.6, 109.8, 108.1, 55.5, 51.9, 51.1, 48.1, 44.0, 43.7, 20.6; IR(KBr) υ:3439, 2953, 2861, 2166, 1710, 1646, 1511, 1496, 1440, 1333, 1263, 1195, 1127, 1029, 908, 838, 814, 778, 730 cm^{-1}; MS (m/z): HRMS (ESI) Calcd. for $C_{30}H_{24}N_3O_5$ ([M - H]$^-$): 506.1732, found: 506.1733.

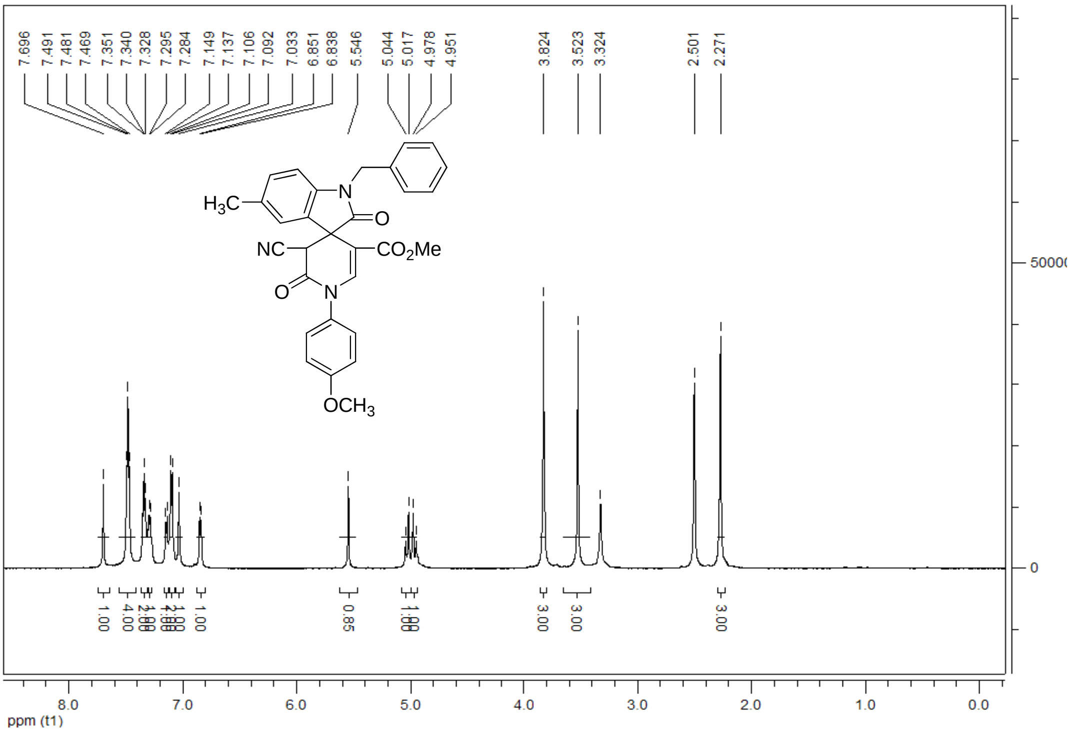

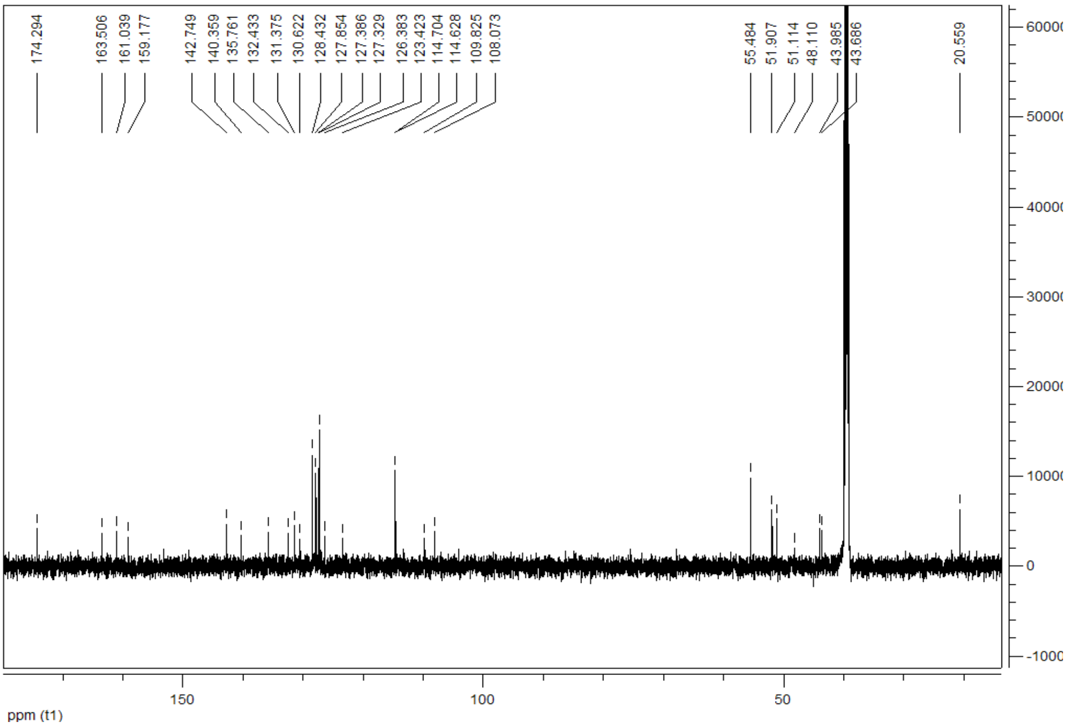

3e: white solid, 56%, m.p. >250 ℃; ^{1}H NMR (600 MHz, DMSO-d_6) δ: 7.72 (s, 1H, ArH), 7.50 (d, J = 7.2 Hz, 2H, ArH), 7.42 (d, J = 7.8 Hz, 2H, ArH), 7.37–7.34 (m, 5H, ArH), 7.31–7.26 (m, 2H, ArH), 7.13–7.09 (m, 1H, ArH), 6.97 (d, J = 7.8 Hz, 1H, ArH), 5.59 (s, 1H, CH), 5.04 (d, J = 16.0 Hz, 1H, CH), 5.01 (d, J = 16.0 Hz, 1H, CH), 3.53 (s, 3H, CH_3), 2.38 (s, 3H, CH_3); ^{13}C NMR (150 MHz, DMSO-d_6) δ: 174.3, 163.5, 160.9, 143.0, 142.7, 142.5, 138.4, 136.1, 135.7, 130.4, 130.0, 128.5, 127.5, 127.4, 127.1, 126.3, 126.2, 123.4, 123.0, 110.0, 108.3, 52.0, 51.0, 44.0, 43.7, 20.7; IR(KBr) υ: 3410, 3078, 2954, 2865, 1712, 1641, 1499, 1441, 1331, 1242, 1175, 1129, 923, 827, 749 cm^{-1}; MS (m/z): HRMS (ESI) Calcd. for $C_{29}H_{22}N_3O_4$ ([M - H]$^-$): 476.1594, found: 476.1593.

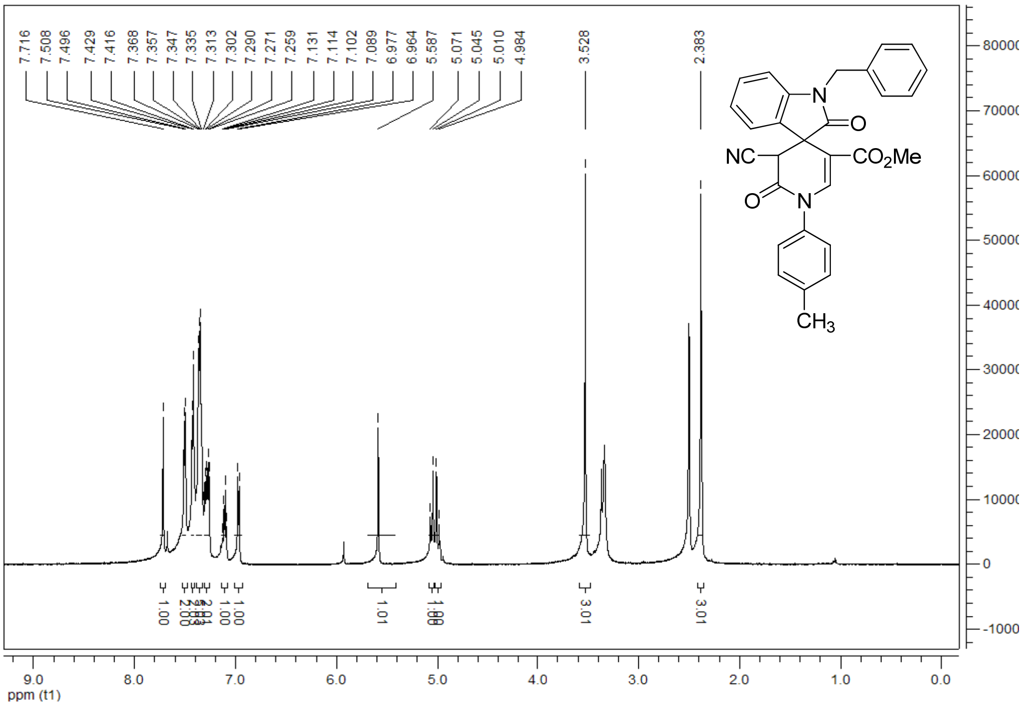

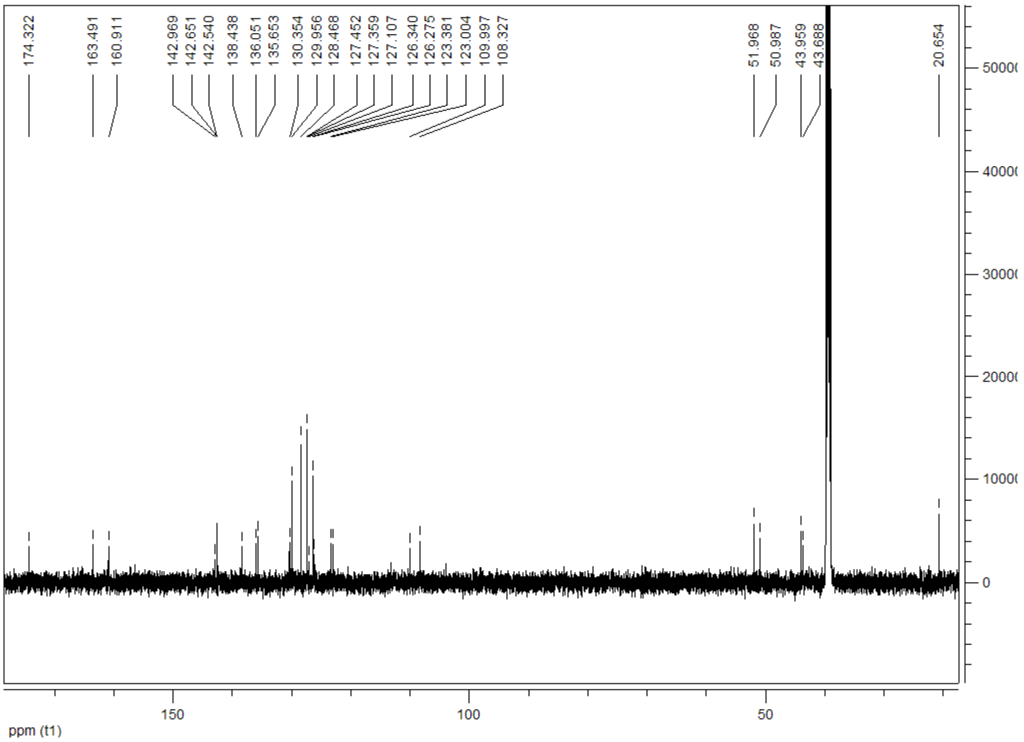

3f: white solid, 47%, m.p. 200–202 ℃; ^{1}H NMR (600 MHz, DMSO-d_6) δ: 7.68 (s, 1H, ArH), 7.42 (d, J = 7.8 Hz, 3H, ArH), 7.35 (d, J = 7.2 Hz, 2H, ArH), 7.25–7.21 (m, 2H, ArH), 7.10 (t, J = 7.8 Hz, 1H, ArH), 5.50 (s, 1H, CH), 3.83–3.80 (m, 1H, CH_2), 3.75–3.71 (m, 1H, CH_2), 3.53 (s, 3H, CH_3), 2.38 (s, 3H, CH_3), 1.66–1.64 (m, 2H, CH_2), 1.45–1.41 (m, 2H, CH_2), 0.93 (t, J = 7.2 Hz, 3H, CH_3); ^{13}C NMR (150 MHz, DMSO-d_6) δ: 173.9, 163.4, 161.0, 143.0, 142.4, 138.4, 136.1, 130.4, 130.0, 129.9, 126.3, 126.1, 123.0, 113.0, 109.5, 108.5, 51.9, 50.9, 44.0, 29.0, 20.7, 19.5, 13.7; IR(KBr) υ: 3398, 3039, 2956, 2868, 1707, 1643, 1607, 1512, 1463, 1335, 1260, 1185, 1130, 1025, 987, 916, 887, 827, 759, 720 cm^{-1}; MS (m/z): HRMS (ESI) Calcd. for $C_{26}H_{25}N_3NaO_4$ ($[M + Na]^+$): 466.1737, found: 466.1737.

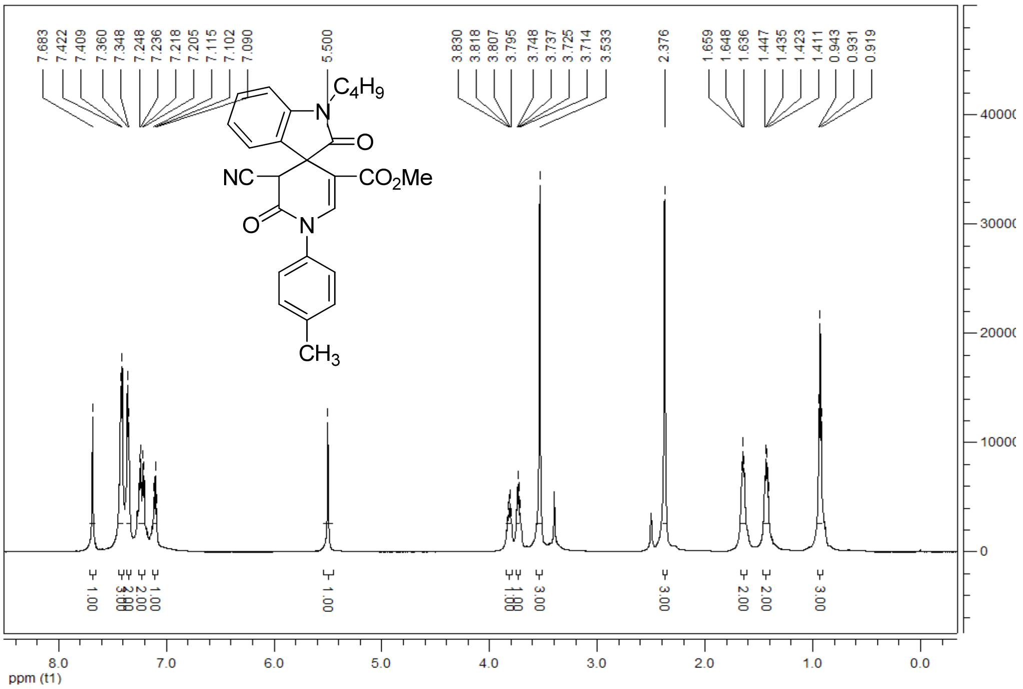

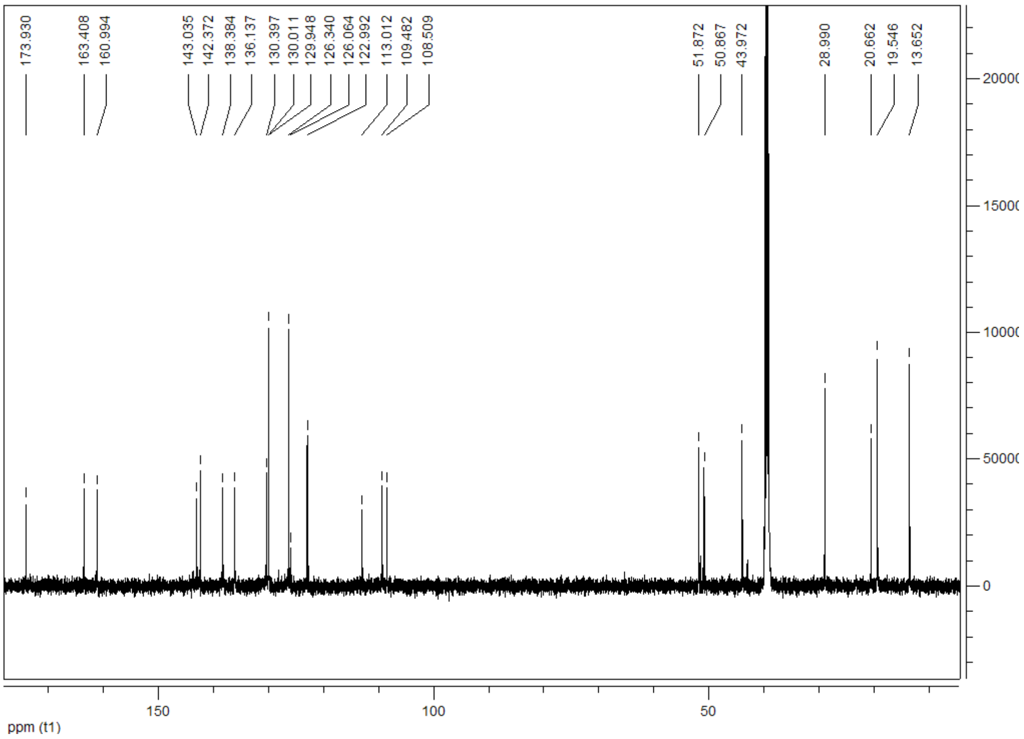

3g: white solid, 49%, m.p. 228–230 ℃; ^{1}H NMR (600 MHz, DMSO-d_6) δ: 7.71 (s, 1H, ArH), 7.49–7.30 (m, 9H, ArH), 7.15 (s, 1H, ArH), 7.02 (s, 1H, ArH), 6.85 (s, 1H, ArH), 5.57 (s, 1H, CH), 5.04–4.95 (m,2H, CH_2), 3.52 (s, 3H, CH_3), 2.39 (s, 3H, CH_3), 2.27 (s, 3H, CH_3); ^{13}C NMR (150 MHz, DMSO-d_6) δ: 174.3, 163.5, 160.9, 142.5, 140.4, 138.4, 136.2, 135.8, 132.4, 130.6, 129.9,128.4, 127.4, 127.3, 126.4, 126.3, 123.4, 117.1, 113.3, 109.8, 108.3, 79.6, 65.8, 51.9, 51.1, 44.0, 43.7, 20.7, 20.6; IR(KBr) υ: 3416, 3034, 2954, 2878, 2166, 1716, 1632, 1495, 1438, 1369, 1342, 1245, 1193, 1153, 1117, 1023, 971, 917, 809, 778, 703 cm^{-1}; MS (*m*/*z*): HRMS (ESI) Calcd. for $C_{30}H_{24}N_3O_4$ ($[M - H]^-$): 490.1782, found: 490.1783.

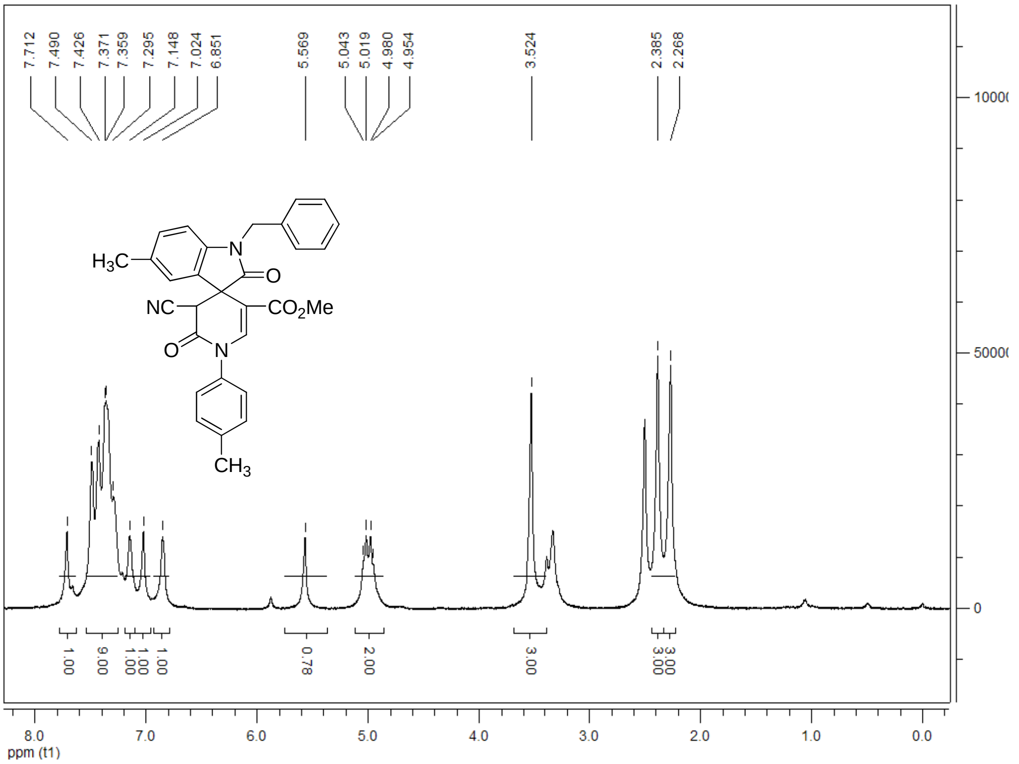

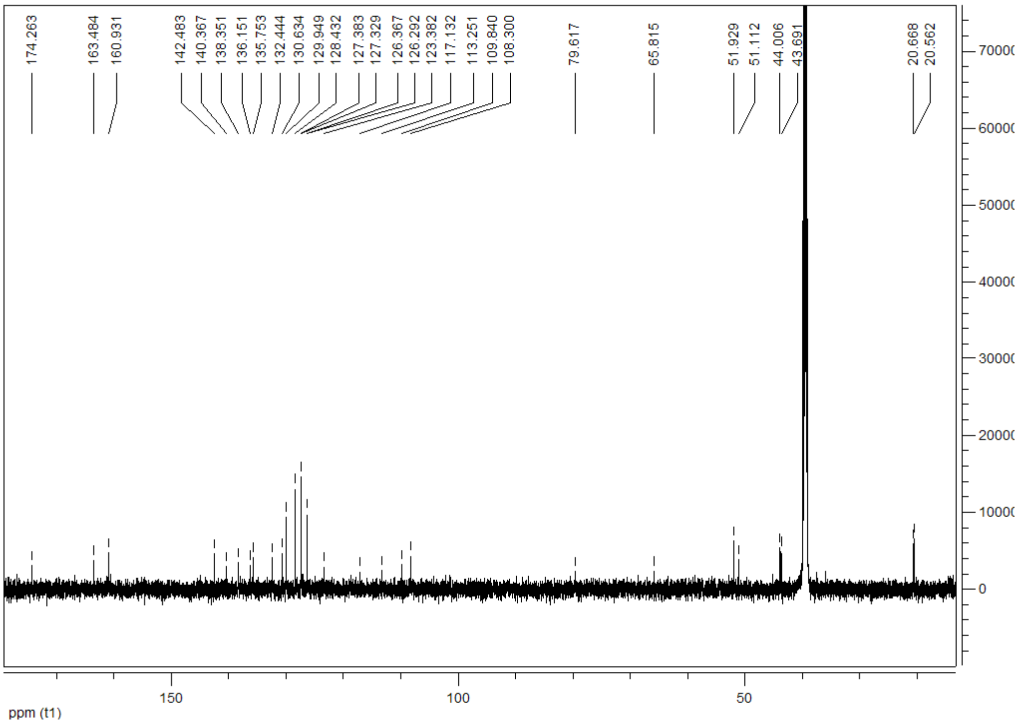

3h: white solid, 56%, m.p. >250 ℃; ^{1}H NMR (600 MHz, DMSO-d_6) δ: 7.71 (s, 1H, ArH), 7.56 (t, J = 7.8 Hz, 4H, ArH), 7.50 (d, J = 6.0 Hz, 1H, ArH), 7.23 (d, J = 7.8 Hz, 1H, ArH), 7.10 (d, J = 7.8 Hz, 1H, ArH), 7.00 (s, 1H, ArH), 5.47 (s, 1H, CH), 3.81–3.76 (m, 1H, CH_2), 3.70–3.67 (m, 1H, CH_2), 3.54 (s, 3H, CH_3), 2.29 (s, 3H, CH_3), 1.64–1.62 (m, 2H, CH_2), 1.42–1.40 (m, 2H, CH_2), 0.93 (t, J = 6.6 Hz, 3H, CH_3); ^{13}C NMR (150 MHz, DMSO-d_6) δ: 173.8, 163.4, 161.0, 142.2, 140.7, 138.7, 132.0, 130.7, 129.5, 128.7, 126.5, 126.4, 123.4, 123.3, 113.1, 109.3, 108.6, 50.9, 44.0, 29.0, 20.6, 19.5, 13.7; IR(KBr) υ: 3433, 3105, 3062, 2952, 2929, 2868, 2026, 1703, 1648, 1622, 1599, 1496, 1440, 1357, 1330, 1295, 1268, 1197, 1125, 989, 915, 892, 824, 776, 757, 713 cm^{-1}; MS (m/z): HRMS (ESI) Calcd. for $C_{26}H_{26}N_3O_4$ ($[M + H]^+$): 444.1918, found: 444.1885.

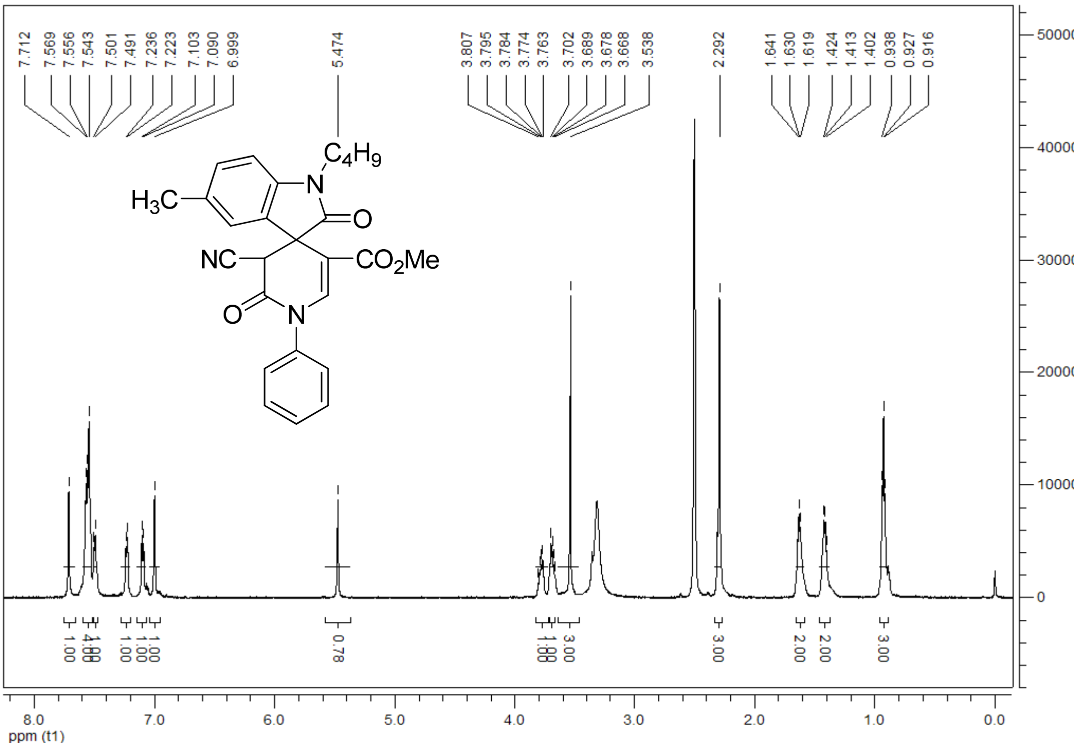

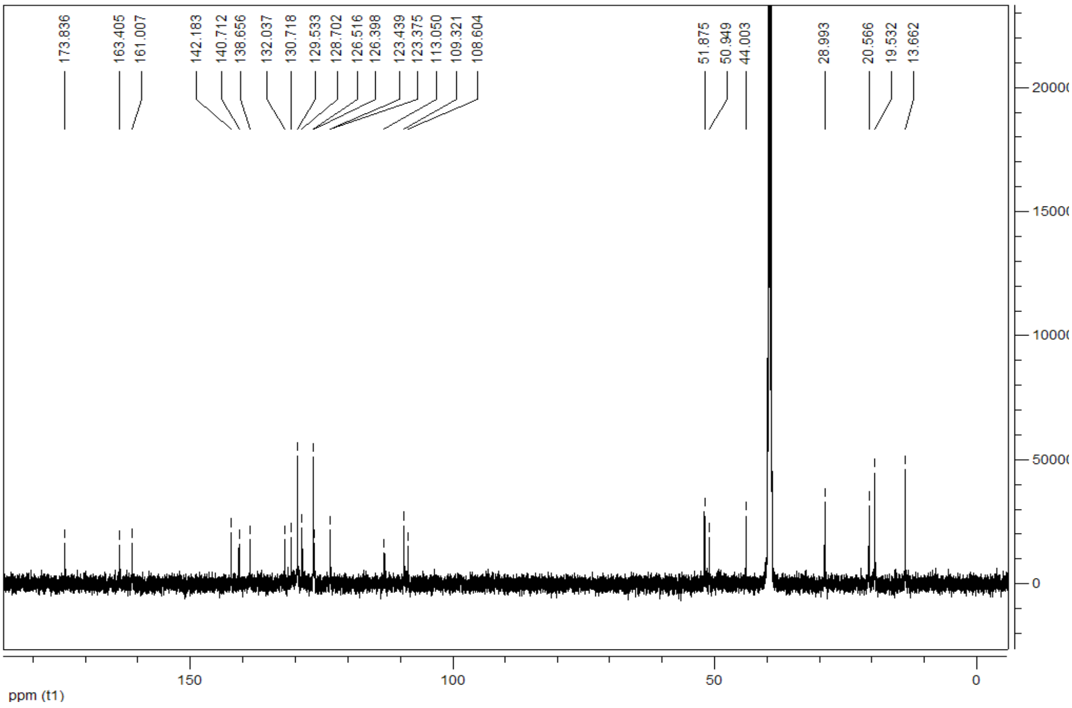

3i: white solid, 47%, m.p. >250 ℃; ^{1}H NMR (600 MHz, DMSO-d_6) δ: 7.77 (s, 1H, ArH), 7.56–7.50 (m, 7H, ArH), 7.40 (d, J = 7.2 Hz, 1H, ArH), 7.35 (t, J = 6.6 Hz, 2H, ArH), 7.29 (t, J = 8.4 Hz, 2H, ArH), 7.11 (t, J = 6.6 Hz, 1H, ArH), 6.98 (d, J = 7.8 Hz, 1H, ArH), 5.61 (s, 1H, CH), 5.08–4.99 (m, 2H, CH_2), 3.54 (s, 1H, CH_3); ^{13}C NMR (150 MHz, DMSO-d_6) δ: 174.3, 163.5, 160.9, 142.7, 142.4, 138.6, 135.7, 130.4, 129.5, 128.8, 128.5, 127.5, 127.4, 127.1, 126.6, 126.3, 123.4, 123.1, 113.2, 110.0, 108.5, 52.0, 51.0, 44.0, 43.7; IR(KBr) υ: 3428, 3097, 3063, 2952, 2924, 2887, 2026, 1705, 1645, 1609, 1490, 1466, 1439, 1371, 1328, 1295, 1267, 1193, 1172, 1129, 1032, 988, 906, 883, 838, 792, 753, 709 cm^{-1}; MS (m/z): HRMS (ESI) Calcd. for $C_{28}H_{22}N_3O_4$ ($[M + H]^+$): 464.1605, found: 464.1574.

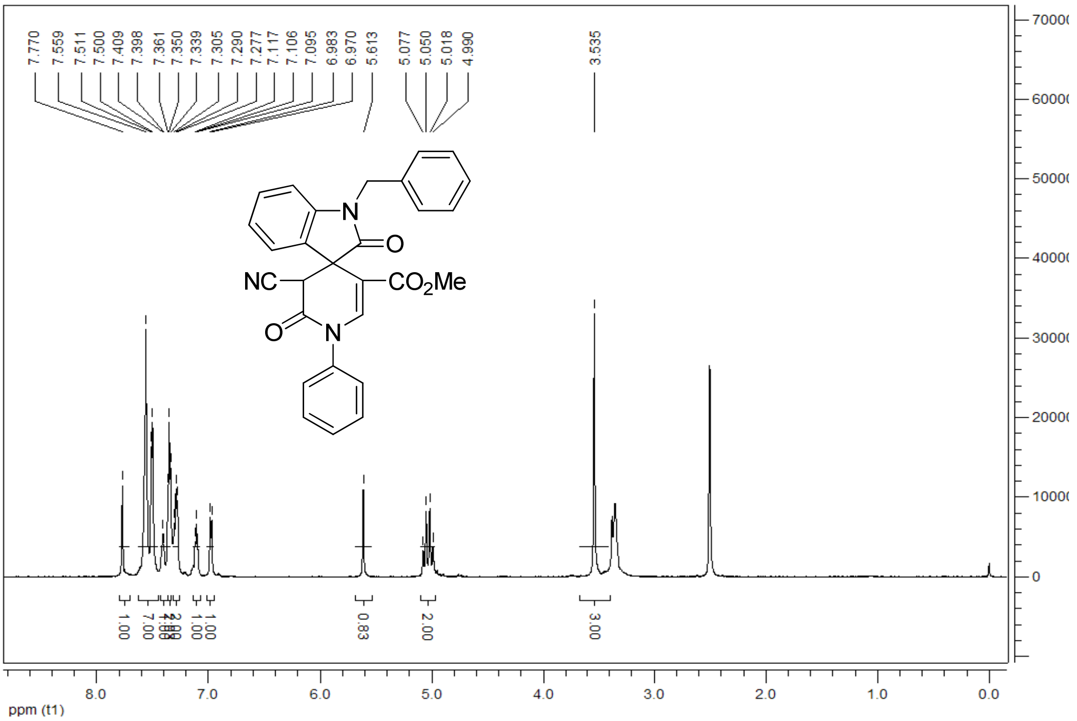

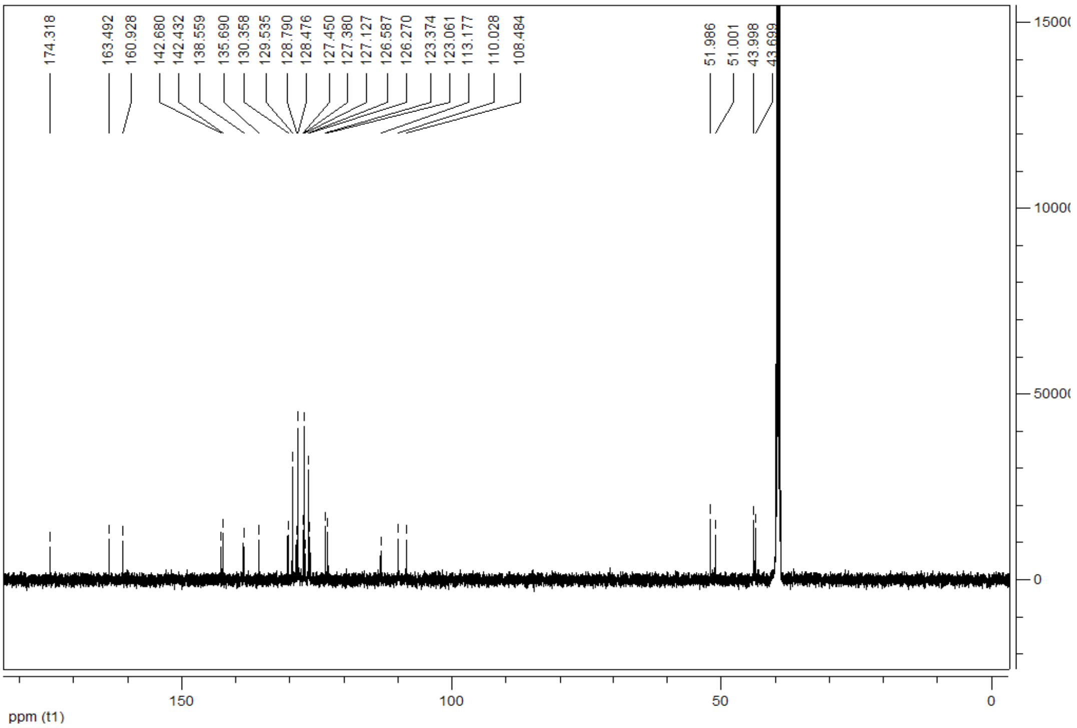

3j: white solid, 52%, m.p. >250 ℃; ^{1}H NMR (600 MHz, DMSO-d_6) δ: 7.77 (s, 1H, ArH), 7.57 (d, J = 5.4 Hz, 4H, ArH), 7.49 (d, J = 7.8 Hz, 2H, ArH), 7.40 (t, J = 7.2 Hz, 1H, ArH), 7.34 (t, J = 7.2 Hz, 2H, ArH), 7.29 (d, J = 7.2 Hz, 1H, ArH), 7.15 (d, J = 7.8 Hz, 1H, ArH), 7.05 (s, 1H, ArH), 6.85 (d, J = 7.8 Hz, 1H, ArH), 5.60 (s, 1H, CH), 5.05–4.95 (m, 2H, CH_2), 3.53 (s, 3H, CH_3), 2.27 (s, 3H, CH_3); ^{13}C NMR (150 MHz, DMSO-d_6) δ: 174.3, 163.5, 160.9, 142.4, 140.4, 138.6, 135.8, 132.5, 130.7, 129.6, 129.5, 128.7, 128.4, 127.4, 127.3, 127.1, 126.5, 126.4, 123.4, 113.3, 109.9, 108.5, 52.0, 51.1, 44.1, 43.7, 20.6; IR(KBr) υ: 3427, 3067, 2951, 2881, 2026, 1703, 1645, 1598, 1495, 1438, 1370, 1329, 1268, 1192, 1124, 1028, 910, 817, 783, 757, 698 cm^{-1}; MS (m/z): HRMS (ESI) Calcd. for $C_{29}H_{24}N_3O_4$ ($[M + H]^+$): 478.1761, found: 478.1737.

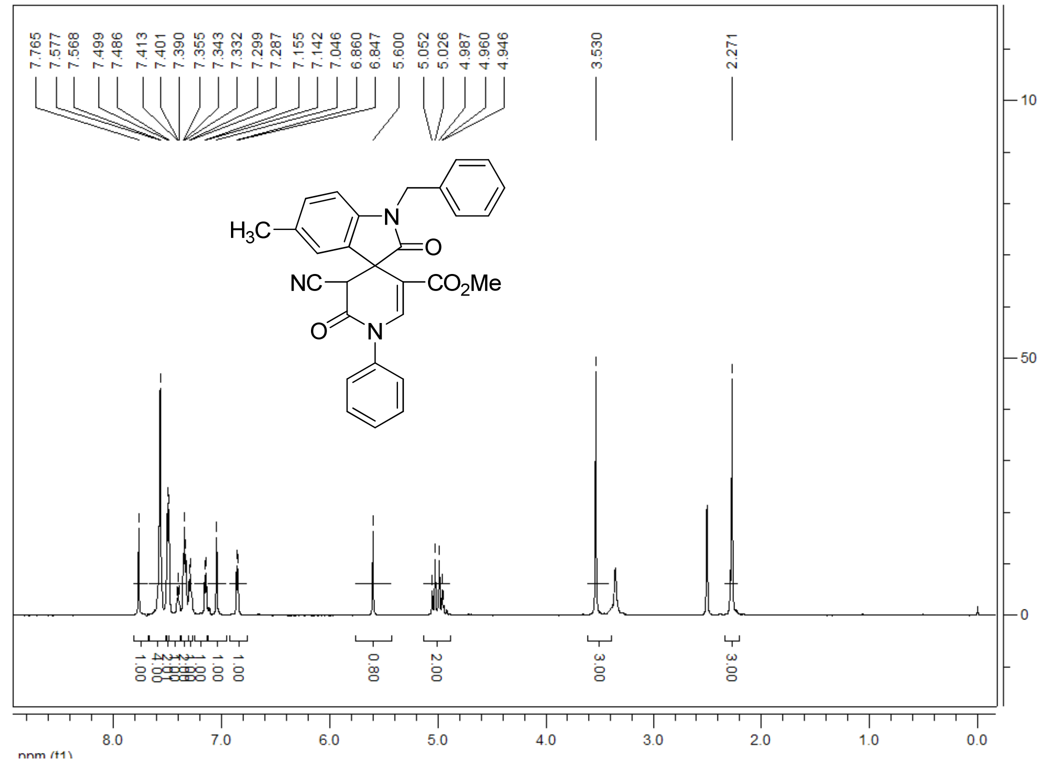

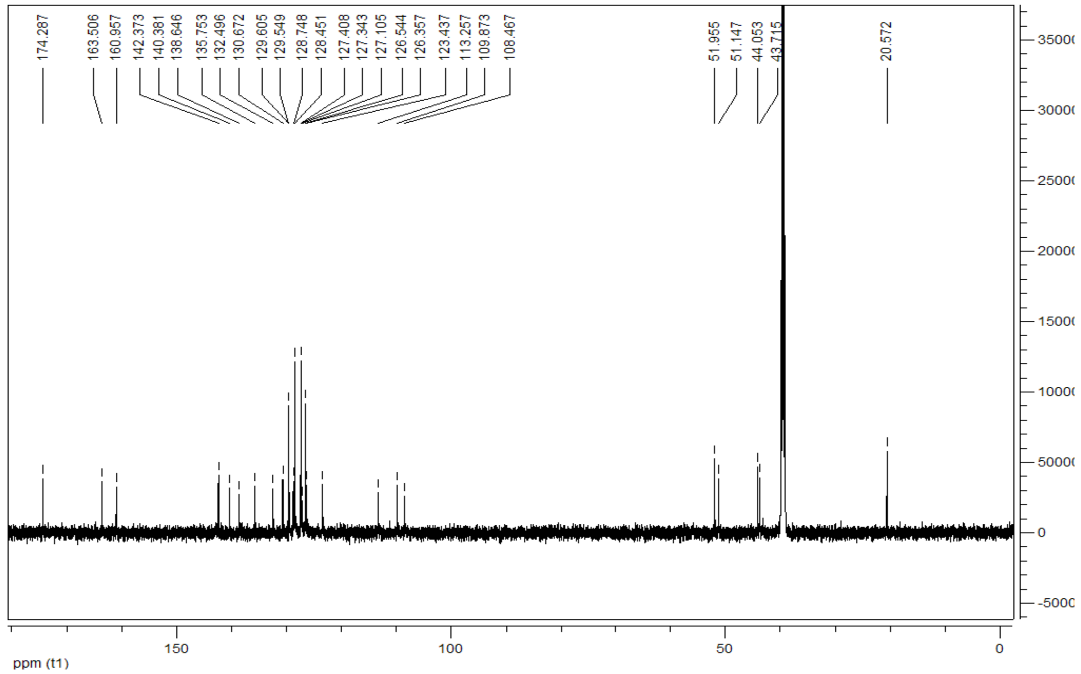

3k: white solid, 68%, m.p. 132–134 ℃; ^{1}H NMR (600 MHz, DMSO-d_6) δ: 10.97 (s, 1H, NH), 7.94 (s, 1H, ArH), 7.46 (d, J = 7.8 Hz, 4H, ArH), 7.40 (d, J = 6.0 Hz, 1H, ArH), 7.24 (t, J = 7.2 Hz, 1H, ArH), 6.91 (d, J = 7.2 Hz, 1H, ArH), 6.76 (t, J = 7.2 Hz, 1H, ArH), 6.47 (d, J = 6.6 Hz, 1H, ArH), 5.32 (s, 1H, CH), 5.00–4.92 (m, 2H, CH_2), 3.56 (s, 3H, CH_3); ^{13}C NMR (150 MHz, DMSO-d_6) δ: 175.6, 163.4, 161.1, 142.2, 141.9, 135.9, 130.1, 128.8, 128.5, 128.4, 128.1, 127.2, 127.0, 122.5, 122.0, 113.4, 110.3, 109.1, 51.8, 51.3, 50.3, 43.5; IR(KBr) υ: 3530, 3421, 3151, 3091, 1717, 1691, 1639, 1474, 1445, 1398, 1367, 1313, 1238, 1187, 1136, 1078, 963, 824, 757, 708 cm^{-1}; MS (m/z): HRMS (ESI) Calcd. for $C_{22}H_{17}N_3NaO_4$ ($[M + Na]^+$): 410.1111, found: 410.1112.

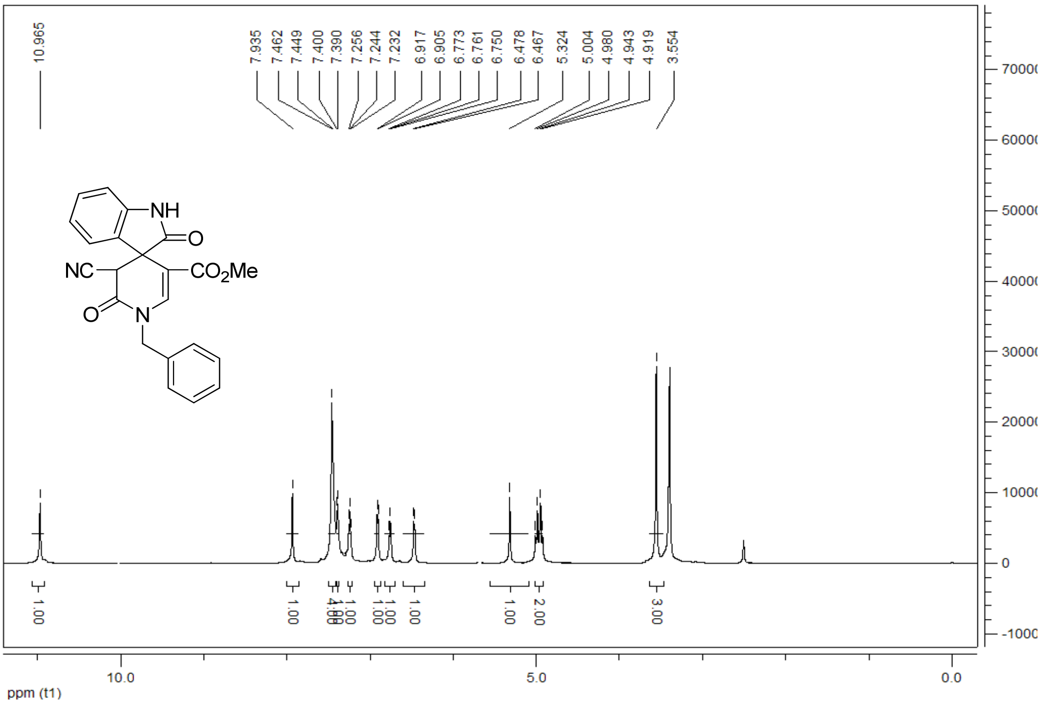

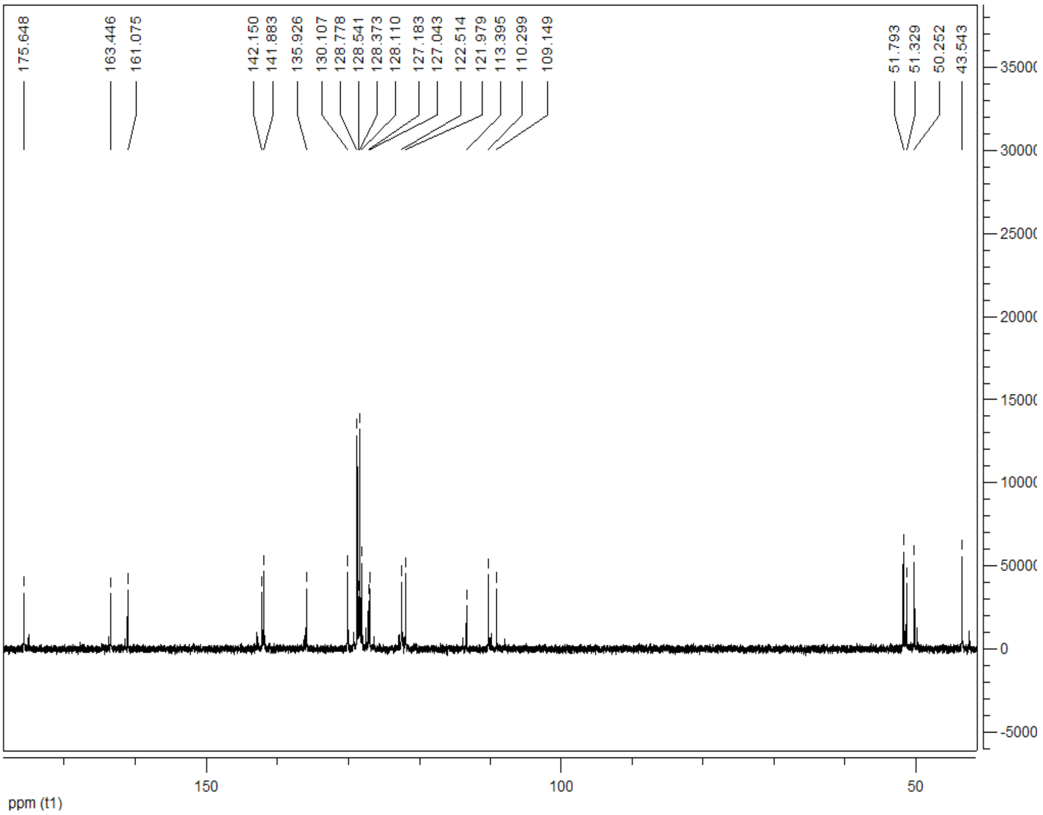

3l: white solid, 45%, m.p. 216–218℃; ^{1}H NMR (600 MHz, DMSO-d_6) δ: 8.06 (s, 1H, ArH), 7.51–7.41 (m, 7H, ArH), 7.31 (s, 2H, ArH), 7.27 (s,1H, ArH), 7.03 (d, J = 5.4 Hz, 1H, ArH), 6.76 (s, 1H, ArH), 6.20 (s, 1H, ArH), 5.43 (s, 1H, CH), 5.07 (d, J = 13.8 Hz, 1H, CH), 4.98 (d, J = 15.0 Hz, 1H, CH), 4.89 (m, 2H, CH_2), 3.53 (s, 3H, CH_3), 1.98 (s, 3H, CH_3); ^{13}C NMR (150 MHz, DMSO-d_6) δ: 174.1, 163.5, 160.9, 142.2, 140.2, 136.0, 135.7, 132.0, 130.4, 128.8, 128.7, 128.6, 128.4, 128.2, 127.4, 127.3, 127.2, 127.0, 126.3, 122.8, 113.5, 109.7, 109.1, 51.9, 50.9, 50.5, 43.6, 43.5, 20.3; IR(KBr) υ: 3404, 2953, 2877, 1704, 1632, 1495, 1438, 1391, 1345, 1297, 1238, 1180, 1154, 1126, 992, 970, 917, 813, 774, 758, 737 cm^{-1}; MS (m/z): HRMS (ESI) Calcd. for $C_{30}H_{24}N_3O_4$ ([M - H]$^-$): 490.1764, found: 490.1763.

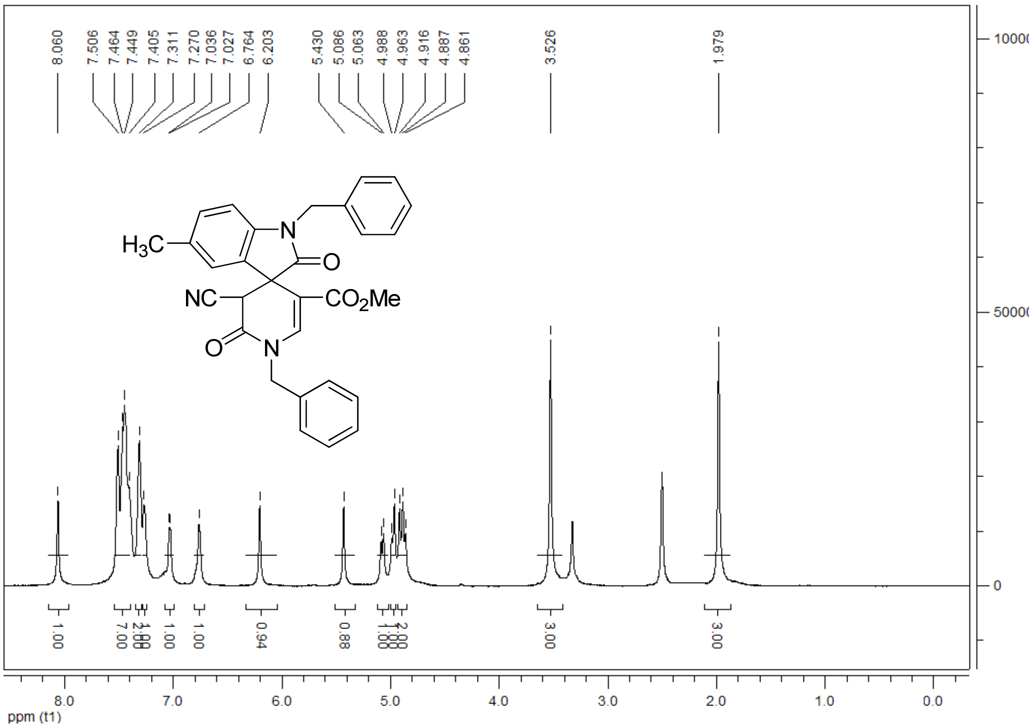

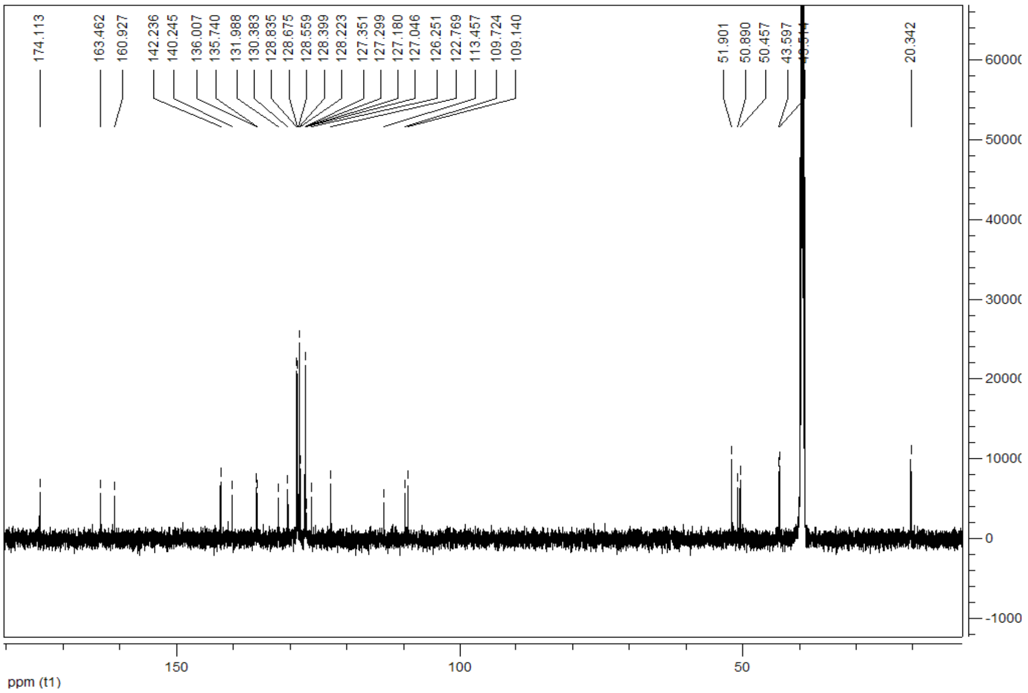

3m: white solid, 63%, m.p. 178–180 ℃; ^{1}H NMR (600 MHz, DMSO-d_6) δ: 7.95 (s, 1H, ArH), 7.47–7.43 (m, 4H, ArH), 7.40 (d, J = 3.6 Hz, 1H, ArH), 7.34 (t, J = 7.8 Hz, 1H, ArH), 7.14 (d, J = 7.8 Hz, 1H, ArH), 6.83 (t, J = 7.2 Hz, 1H, ArH), 6.54 (d, J = 7.2 Hz, 1H, ArH), 5.40 (s, 1H, CH), 5.00–4.94 (m, 2H, CH_2), 3.78–3.75 (m, 1H, CH_2), 3.70–3.66 (m, 1H, CH_2), 3.53 (s, 3H, CH_3), 1.61–1.59 (m, 2H, CH_2), 1.41–1.38 (m, 2H, CH_2), 0.91 (t, J = 6.6 Hz, 3H, CH_3); ^{13}C NMR (150 MHz, DMSO-d_6) δ: 173.9, 163.4, 161.0, 143.0, 142.1, 135.9, 130.2, 128.8, 128.6, 128.4, 128.1, 127.2, 126.4, 122.5, 122.3, 113.2, 109.4, 108.9, 51.8, 50.7, 50.2, 43.5, 29.0, 19.5, 13.6; IR(KBr) υ: 3453, 2959, 2862, 2374, 2025, 1708, 1626, 1485, 1456, 1394, 1356, 1302, 1236, 1184, 1130, 1074, 980, 926, 790, 752 cm^{-1}; MS (m/z): HRMS (ESI) Calcd. for $C_{26}H_{25}N_3NaO_4$ ($[M + Na]^+$): 466.1737, found: 466.1737.

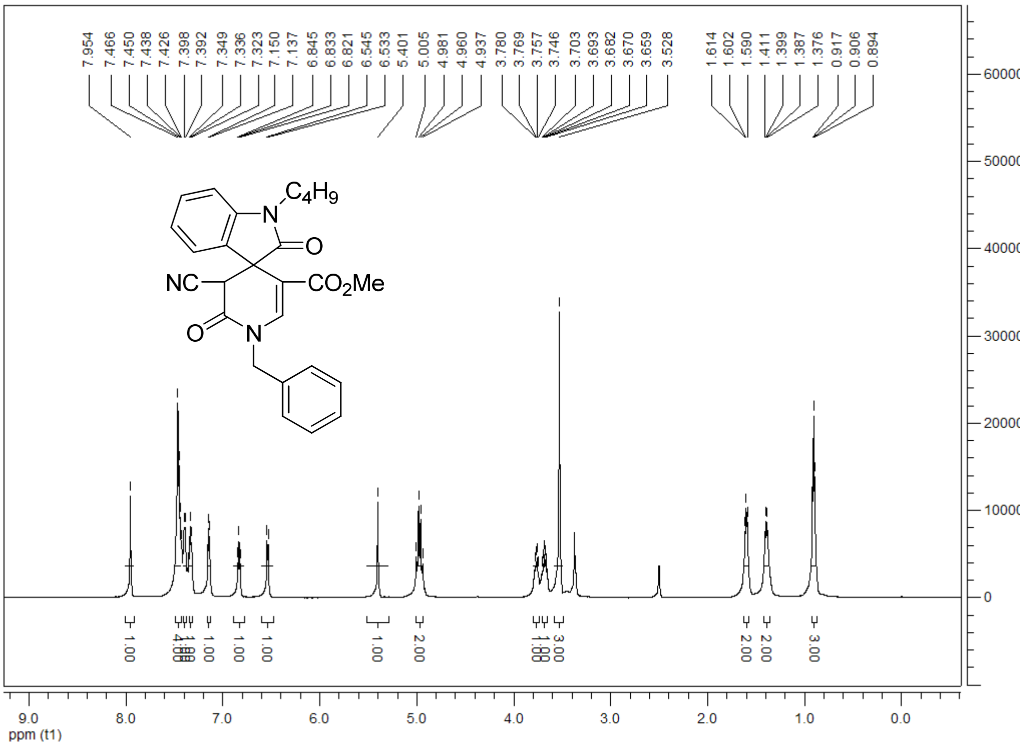

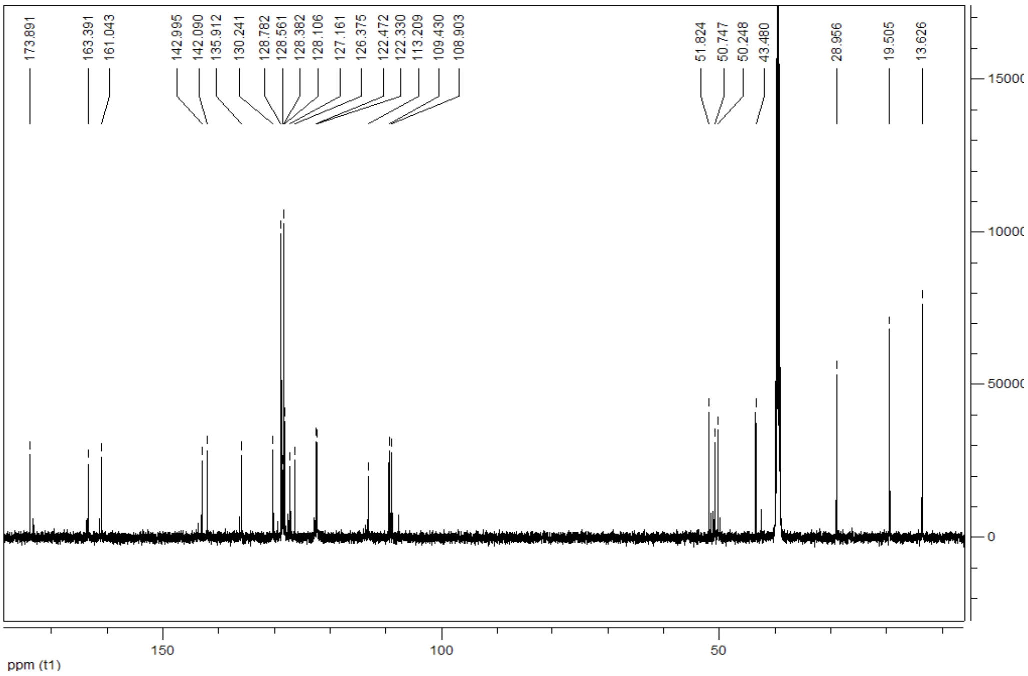

3n: white solid, 48%, m.p. 204–206℃; ^{1}H NMR (600 MHz, DMSO-d_6) δ: 7.90 (s, 1H, ArH), 7.47 (d, J =7.2 Hz, 2H, ArH), 7.37–7.32 (m, 6H, ArH), 7.27 (t, J = 6.6 Hz, 3H, ArH), 6.95 (t, J = 7.2 Hz, 1H, ArH), 6.90 (d, J = 7.8 Hz, 1H, ArH), 6.70 (d, J = 7.2 Hz, 1H, ArH), 5.29 (s, 1H, CH), 5.01 (d, J = 15.8 Hz, 1H, CH), 4.94 (d, J = 15.8 Hz, 1H, CH), 4.09~4.05 (m, 1H, CH), 4.00–3.95 (m, 1H, CH), 3.52 (s, 3H, CH_3), 3.03 (t, J = 7.2 Hz , 2H, CH); ^{13}C NMR (150 MHz, DMSO-d_6) δ: 174.5, 163.6, 160.9, 142.8, 142.6, 137.7, 135.7, 130.1, 128.9, 128.5, 128.4, 127.4, 127.3, 127.1, 126.6, 126.5, 123.1, 122.7, 113.4, 109.8, 107.4, 51.8, 50.7, 48.4, 43.6, 43.4, 33.8; IR(KBr) υ: 3404, 3028, 2952, 2864, 1703, 1646, 1613, 1484, 1455, 1367, 1319, 1227, 1183, 1133, 1024, 914, 747, 702 cm^{-1}; MS (m/z): HRMS (ESI) Calcd. for $C_{30}H_{24}N_3O_4$ ([M - H]$^-$): 490.1772, found: 490.1773.

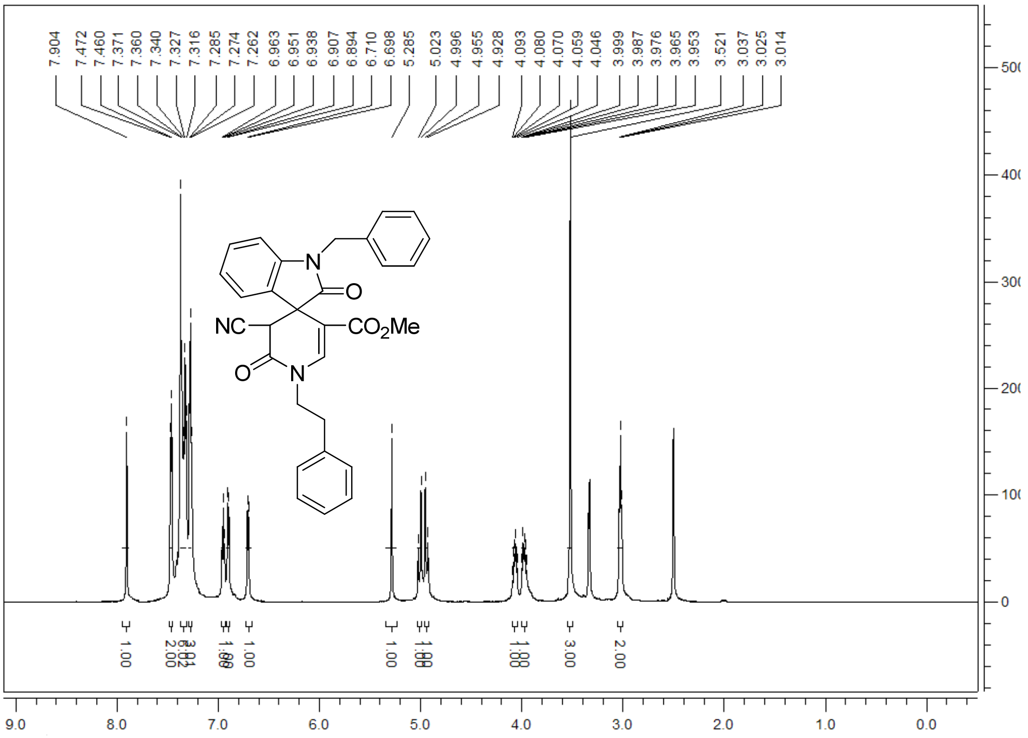

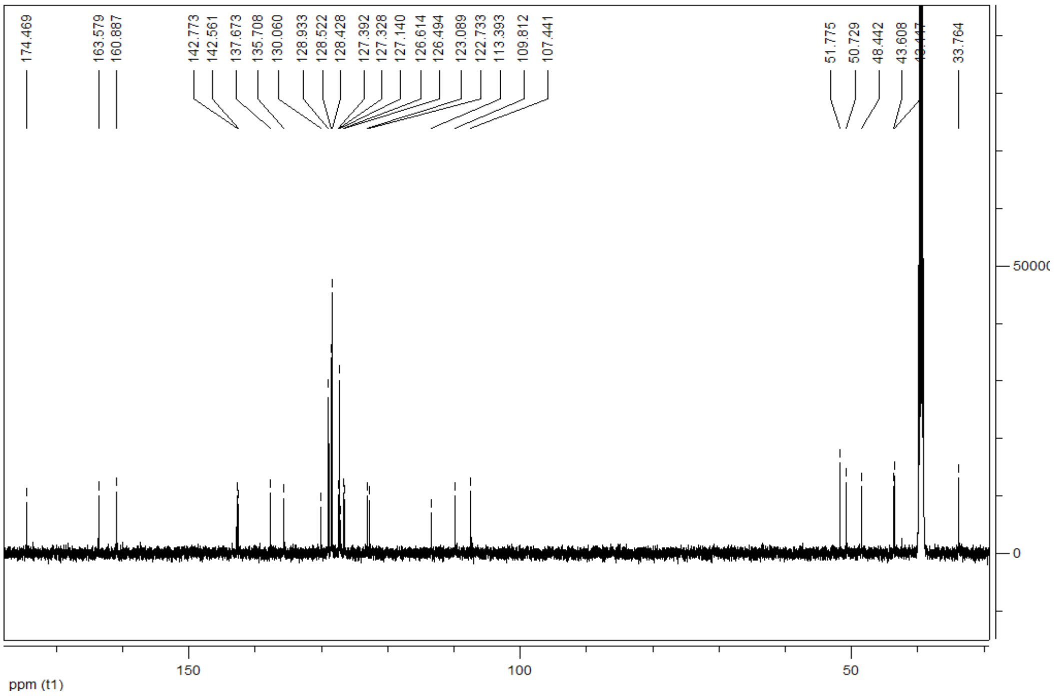

Reference:

1. Sun, J.; Wu, Q.; Zhang, L. J.; Yan, C. G. *Chin. J. Chem.* **2012,** *30,* 1548–1554. doi:10.1002/cjoc.201100657
